# Large-scale phylogenomic analysis suggests three ancient superclades of the WUSCHEL-RELATED HOMEOBOX transcription factor family in plants

**DOI:** 10.1371/journal.pone.0223521

**Published:** 2019-10-11

**Authors:** Cheng-Chiang Wu, Fay-Wei Li, Elena M. Kramer

**Affiliations:** 1 Department of Organismic and Evolutionary Biology, Harvard University, Cambridge, Massachusetts, United States of America; 2 Boyce Thompson Institute, Ithaca, New York, United States of America; 3 Section of Plant Biology, Cornell University, Ithaca, New York, United States of America; Michigan State University, UNITED STATES

## Abstract

The adaptation of plants to land required multiple morphological innovations. Among these include a variety of lateral organs that are initiated from apical meristems, in which the mantainance of undifferentiated stem cells is regulated by the homeodomain WUSCHEL-RELATED (WOX) transcription factors. Expansion of the *WOX* gene family has been associated with whole genome duplication (WGD) events and postulated to have been pivotal to the evolution of morphological complexity in land plants. Previous studies have classified the *WOX* gene family into three superclades (e.g., the ancient clade, the intermediate clade, and the modern clade). In order to improve our understanding of the evolution of the *WOX* gene family, we surveyed the *WOX* gene sequences from 38 genomes and 440 transcriptomes spanning the Viridiplantae and Rhodophyta. The WOX phylogeny inferred from 1039 WOX proteins drawn from 267 species with improved support along the backbone of the phylogeny suggests that the plant-specific WOX family contains three ancient superclades, which we term Type 1 (T1WOX, the WOX10/13/14 clade), Type 2 (T2WOX, the WOX8/9 and WOX11/12 clades), and Type 3 (T3WOX, the WUS, WOX1/6, WOX2, WOX3, WOX4 and WOX5/7 clades). Divergence of the T1WOX and T2WOX superclades may predate the diversification of vascular plants. Synteny analysis suggests contribution of WGD to expansion of the *WOX* family. Promoter analysis finds that the capacity of the *WOX* genes to be regulated by the auxin and cytokinin signaling pathways may be deeply conserved in the Viridiplantae. This study improves our phylogenetic context for elucidating functional evolution of the *WOX* gene family, which has likely contributed to the morphological complexity of land plants.

## Introduction

The radiation of plants in their quest for land was accompanied by morphological innovations, such as 3D growth, roots, leaves, and flowers [1–4]. These morphological novelties are initiated from apical or axillary meristems that contain undifferentiated stem cells [5]. Meristem development is controlled by the WUSCHEL-RELATED HOMEMOBOX (WOX) transcription factors. The WOX proteins share a DNA-biding homeodomain (HD) of 60–66 amino acids [6, 7], while other regions of the *WOX* coding regions are highly divergent in sequence. In vascular plants, the maintenance of the stem cell niche in a shoot apical meristem (SAM) is regulated by the *WUSCHEL* (*WUS*) gene of the *WOX* family and its partners of the WUS-CLAVATA (WUS-CLV) signaling pathway [8–15]. *WUS* also promotes stem cell proliferation in floral meristems, and helps activate the Type 2 MADS-box gene *AGAMOUS* (*AG*), which specifies reproductive floral organ identity and determinate growth of floral meristems, in collaboration with another transcription factor, *LEAFY* (*LFY*) [16–19]. Another *WOX* gene, *WOX5*, maintains the stem cell niche in the root apical meristem (RAM) [12, 20–22]. *WOX5* expression in the quiescent center (QC) of root [23] is regulated by the protein complexes of (1) the double APETALA2 (AP2)-domain transcription factors PLETHORAs (PLTs) [24–26], (2) the GRAS family transcription factor SCARECROW (SCR)[27, 28], and (3) the TEOSINTE-BRANCHED CYCLOIDEA PCNA (TCP) transcription factors [29, 30], which bind to the PLT-binding site in the *WOX5* promoter [31]. Besides *WUS* and *WOX5*, the other 13 members of the *WOX* gene family in Arabidopsis (*Arabidopsis thaliana*), except *WOX10*, have been funcitonally characterized to regulate meristem development in embryos, as well as vegetative and reproductive organs (Table 1).

**Table 1:** Summary of Arabidopsis *WOX* gene expression patterns and functions.

Gene	Expression pattern	Function	Reference
*AtWUS*	Shoot apical meristem; anther; ovule	Stem cell maintenance; leaf, anther and ovule development	[8, 9, 18, 32–34]
*AtWOX1*	Leaf primordium; procambial tissue	Leaf, sepal, and petal development	[34, 35]
*AtWOX2*	Eggs; zygote; apical embryo domain	Embryo patterning	[36]
*AtWOX3*	Leaf primordium; floral primordium; sepal and petal primordia	Leaf, sepal, and petal development	[34, 35, 37]
*AtWOX4*	Inflorescence stem; leaf primordium; flower	Procambial development	[38, 39]
*AtWOX5*	Root apical meristem	Stem cell maintenance	[21]
*AtWOX6*	Seedling; ovule primordium	Ovule development; freezing tolerance	[40, 41]
*AtWOX7*	Root apical meristem	Stem cell maintenance; sugar response	[42]
*AtWOX8*	Zygote; basal embryo domain	Embryo patterning	[36, 43]
*AtWOX9*	Zygote; basal embryo domain	Embryo patterning	[36, 43]
*AtWOX10*	Unknown	Unknown	
*AtWOX11*	Root apical meristem	Root organogenesis	[44]
*AtWOX12*	Root apical meristem	Root organogenesis	[44]
*AtWOX13*	Root; inflorescence	Root development; floral transition	[45]
*AtWOX14*	Root; inflorescence	Root development; floral transition	[45]

Given the pivotal roles of the *WOX* gene family in meristem development, the evolution of the *WOX* gene family has been associated with morphological innovations [12, 13, 46–51]. Previous phylogenetic studies of the *WOX* gene family based on the homeodomain or full-length sequences identified three *WOX* superclades, termed the ancient, intermediate and modern clades [12, 13, 47, 52–55]. Three characteristic peptide motifs in the HD were suggested as signatures of these superclades: NVYNWFQNR of the ancient clade, NVFYWFQNR of the intermediate clade, and NVFYWFQNH of the modern clade [13, 56]. Proteins of the modern clade (WUS, WOX1/6, WOX2, WOX3, WOX4, and WOX5/7 subclades, named after their Arabidopsis members) share a WUS-box motif [7], and the WUS, WOX5 and WOX7 proteins additionally contain an ERF-associated amphiphilic repression (EAR) motif in their carboxy (C)-termini [57, 58]. The EAR motif interacts with the TOPLESS (TPL)/TPL-Related (TPR) co-repressors to repress the transcription of auxin response genes [59]. The intermediate clade includes the WOX8/9 and WOX11/12 subclades, which share the VFIN WOX8 MOG and LQxG WOX8 MOG motifs in their C-termini, while the WOX10/13/14 proteins of the ancient clade contain the WOX13 MOG motif of 39 amino acids upstream of the HD [45]. However, these phylogenetic analyses have been restricted by (1) the nature of the *WOX* sequences, (2) limited available sampling spanning the Viridiplantae, and (3) a lack of robust sequence alignment method; meaning that the origin and relationship among clades and subclades remain unclear. For instance, the “ancient” clade has been frequently inferred as paraphyletic and lacking support [12, 13, 52, 53, 55]. In addition, a polytomy has been commonly reconstructed along the backbone of the modern clade [12, 13, 47, 49, 52, 53, 55, 60]. For example, a recent phylogenetic reconstruction based on an alignment of 350 WOX proteins compiled with MUSCLE [61] and manual adjustment showed that the braches leading to all three superclades, as well as to all clades in the modern clade, have bootstrap support (BS) below 50 [53]. Consequently, the interpretation of experimental data on the functional divergence of the *WOX* genes is challenging [13, 15, 62].

The *WOX* function is regulated in part by the auxin and cytokinin signaling pathways [63]. The AUXIN RESPONSE FACTOR (ARF) transcription factors of the canonical TRANSPORT INHIBITOR-RESPONSE 1 (TIR1)-AUXIN/INDOLE-3-ACETIC ACID (AUX/IAA)-ARF pathway [36] activate or repress the *WOX* genes by binding to Auxin Response Elements (AuxREs) [64–67] in the *WOX* promoters [44, 68–72]. On the other hand, cytokinin activates *WUS* expression through direct binding of the type-B ARABIDOPSIS RESPONSE REGULATORs (ARR-Bs) to the B-ARR motif [73–75] in the *WUS* promoter [76–79]. ARR10, which is one of the ARR-Bs, also binds to the B-ARR motif in the promoters of *WOX1* and *WOX12* [77]. However, it is unclear how evolutionarily conserved the regulation of the *WOX* function by these two major phytohormone signaling pathways is.

In order to explore the origin and evolution of the *WOX* gene family, we developed a robust bioinformatics pipeline to compile the most comprehensive sampling of the WOX protein sequences to date for phylogenetic reconstruction without manual adjustment, including 38 genomes and 440 transcriptomes covering most extant Viridiplantae (i.e., chlorophytes, charophytes, bryophytes, lycophytes, ferns, gymnosperms, and angiosperms) orders and Rhodophyta (i.e., red algae). Conserved protein motifs of the WOX clades, as well as AuxREs and B-ARR motifs in target *WOX* promoters, were identified. The reconstructed WOX phylogeny inferred three ancient superclades of this pivotal family of transcription factors, and provides the phylogenetic context for research in its genetic and biochemical evolution, which underpins the evolution of morphological complexity in plants.

## Materials and methods

### Phylogenetic reconstruction

Sequences of 15 Arabidopsis WOX proteins were used as queries to BLAST against the coding sequences (CDSs) of 360 transcriptomes from the 1KP database by using the Python pipeline BlueDevil with E-value cutoff of 1e-5 [80], 29 published genomes of land plants from Phytozome 10 and CoGe [81, 82], and the fern genomes of *Azolla filiculoides* and *Salvinia cucullata* [83] by using the BLAST+ package with tBLASTn algorithm and E-value cutoff of 1e-5 [84]. Another 79 transcriptomes of red and green algae from 1KP and 7 genomes (*Chlamydomonas reinhardtii*, *Coccomyxa subellipsoidea*, *Klebsormidium nitens*, *Micromonas pusilla CCMP1545*, *Micromonas sp*. *RCC299*, *Ostreococcus lucimarinus* and *Volvox carteri*) from Phytozome 10 and *Klebsormidium flaccidum* genome project[85] were also BLASTed against for *WOX* homologs. The retrieved sequences and an additional six *WOX* coding sequences from the *Gunnera manicata* transcriptome (Chiu and Elhai, unpublished) were translated into protein sequences. 1098 WOX protein sequences with lengths between 120 and 971 amino acids were aligned using PASTA [86] and then filtered by the two sequential criteria: (1) aligned columns with more than 50% missing data were removed, and (2) sequences filtered from (1) with less than half of the total alignment length were removed. This procedure of alignment and filtering was taken six times until no sequences were filtered out. Phylogenetic reconstruction of the compiled protein alignment, WOXaa (S1 File), of 1039 sequences with 145 sites in length (S1 Table) was performed by RAxML 8.2.4 [87] under the JTT model with gamma-distribution of rate variation among sites, which was selected by ProtTest 3.4.2 [88], with 1,000 BS replicates on the CIPRES Science Gateway [89]. Chlorophyte WOX proteins from *Bathycoccus prasinos*, *Ostreococcus lucimarinus*, *Micromonas pusilla CCMP1545* and *Micromonas sp*. *RCC299* were used as outgroup for rooting the WOX phylogeny. In order to test whether the reconstructed topology would be consistent between analysis of the WOXaa dataset and analysis of WOX sequences retrieved only from candidate genomes (i.e., not from transcriptomes), we prepared another dataset with 446 WOX proteins from candidate genomes. This smaller dataset was aligned and filtered following the aforementioned criteria to generate an alignment (WOXaa_g) of 442 sequences with 150 sites in length (S2 File) for phylogenetic reconstruction. The phylogenetic reconstruction based on WOXaa_g was conducted following the aforementioned approach.

### Search of WOX motifs

Motifs shared by clades of the WOX proteins were discovered by using MEME 4.11.2 [90] with E-value cutoff of 1e-5 and minimum length of five amino acids on the Odyssey cluster supported by the FAS Division of Science, Research Computing Group at Harvard University.

### Synteny analysis

In order to examine whether clades with weak phylogenetic resolution were derived from genome duplication, we performed synteny analysis by using the SynFind program [91] with default setting on CoGe [81, 82]. A syntenic score is defined as the number of homologous genes shared within the vicinity of a total of 41 genes (the anchor gene and its 20 upstream and 20 downstream). A retrieved genomic region is determined syntenic with a syntenic score of at least 4. A syntenic proxy is referred if the gene in the query is lost in the syntenic region.

### Search of AuxRE and B-ARR-6-BA motifs, and the *ARF* and *ARR-B* genes

Genomic sequences 1.5kb upstream of the transcription start sites (TSS) of all *WOX* genes from *O*. *lucimarinus*, *M*. *pusilla CCMP1545*, *Micromonas sp*. *RCC299*, *M*. *polymorpha*, *P*. *patens*, *S*. *Moellendorffii*, *A*. *trichopoda*, *A*. *coerulea*, and Arabidopsis were retrieved from Phytozome 12.1 for prediction of AuxRE and B-ARR-6-BA motifs by PlantPAN 2.0[92]. Overlapping sites of each motif were counted only once. To investigate the presence of the *ARF* and *ARR-B* genes, all Arabidopsis ARF and ARR-B protein sequences (S2 Table) were used as queries to BLAST against a CDS database of eight genomes (e.g., *A*. *thaliana*, *Solanum lycopersicum*, *Aquilegia coerulea*, *Amborella trichopoda*, *Selaginella moellendorffii*, *Marchantia polymorpha*, *Physcomitrella patens*, and *M*. *pusilla*) from Phytozome 12.1 by using the BLAST+ package with tBLASTn algorithm. The retrived CDS sequences were translated into protein sequences using EMBOSS Transeq (https://www.ebi.ac.uk/Tools/st/emboss_transeq/). The protein sequences were run through InterPro 71.0 (https://www.ebi.ac.uk/interpro/) for search of conserved domains as verification.

## Results

### Major clades of the *WOX* gene family are ancient

**Fig 1:**
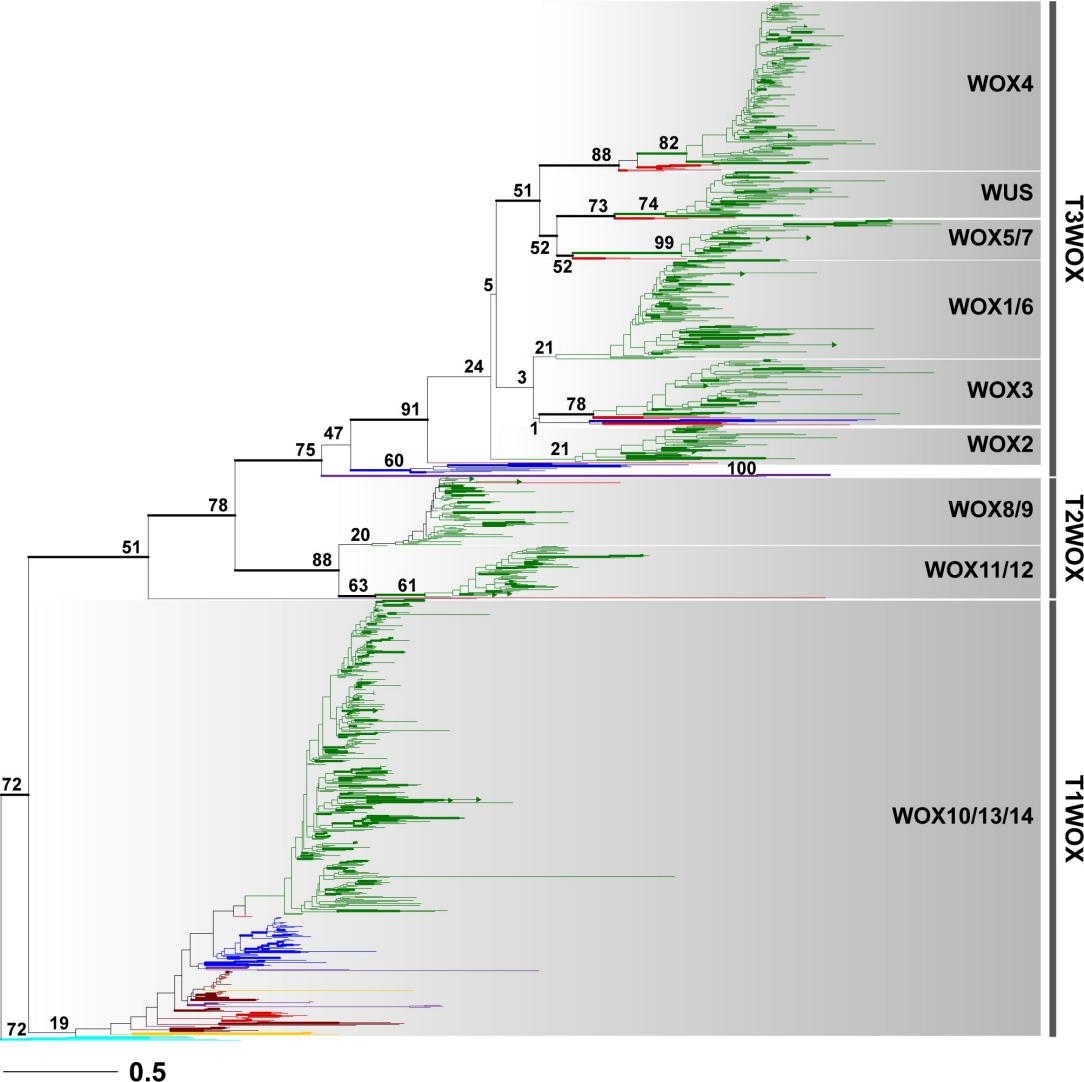
Maximum-likelihood (ML) phylogeny of the WOX protein family. The ML phylogeny was reconstructed by RAxML 8.2.4 with 1,000 bootstrap replicates. Thicker branch width indicates BS support equal or greater than 50. BS values are shown at key branches. Branches are colored according to taxonomic affiliation: green for angiosperms; red, gymnosperms; blue, ferns; purple, lycophytes; brown, bryophytes; yellow, charophytes; cyan, chlorophytes. Clades are shaded in a gradient of gray. Major superclades are marked with vertical lines. The scale is amino acid substitution rate of 0.5.

In order to elucidate the origin and deep divergence of major WOX clades, we compiled an alignment (WOXaa) of 1039 WOX proteins from 267 species covering all divisions of the Viridiplantae after trimming columns of low occupancy and short sequences. The alignment has a length of 145 amino acids with 21.01% of characters as gaps. No *WOX* gene was found in any of the 26 rhodophyte transcriptomes sampled by 1KP. Among the 45 transcriptomes and 3 genomes of sampled chlorophytes, the WOX proteins were retrieved from 9 species (*Bathycoccus prasinos*, *Codium fragile*, *M*. *pusilla CCMP1545*, *M*. *RCC299*, *Ostreococcus lucimarinus*, *Picocystis salinarum*, *Scherffelia dubia*, *Scourfieldia sp*. and *Trebouxia arboricola*). Rooted with a sampling of chlorophyte WOX sequences, the Maximum-likelihood (ML) phylogeny inferred from WOXaa is divided into three superclades, which we term Type 1 (T1WOX), Type 2 (T2WOX) and Type 3 (T3WOX). A sequence from *Selaginella moellendorffii* 417553 is sister to the T2WOX + T3WOX clade (Fig 1). The *T1WOX* superclade comprises of the WOX10/13/14 proteins from all divisions of the Viridiplantae. The T1WOX superclade has low support (BS 19), which is consistent with previous studies [12, 52–54, 60]. The well-supported T2WOX superclade includes the WOX8/9 and WOX 11/12 clades, which were previously referred to as the intermediate clade. The T2WOX superclade is sister to the T3WOX superclade with BS support of 78. The T3WOX superclade (BS 75) contains the WUS, WOX5/7, WOX3, WOX1/6, WOX4, and WOX2 clades, as well as some lycophyte, fern and gymnosperm WOX proteins that are sister to the aforementioned angiosperm clades.

In order to test the topological consistency of the inferred WOX phylogeny, we applied the same alignment and reconstruction approach to a smaller dataset WOXaa_g in which the WOX proteins were drawn from candidate genomes only. The phylogeny inferred from WOXaa_g recovers three superclades and conserved topology among the T3WOX clades (S1 Fig). However, there are several major differences. For instance, the WOX8/9 proteins, as well as several fern T3WOX proteins, are paraphyletic to the WOX11/12 clade in the WOXaa_g phylogeny. In addition, there is no lycophyte WOX sequence nested within the T3WOX superclade. Generally BS supports at the backbone of the WOXaa_g phylogeny are lower than those of the WOXaa phylogeny.

**Fig 2:**
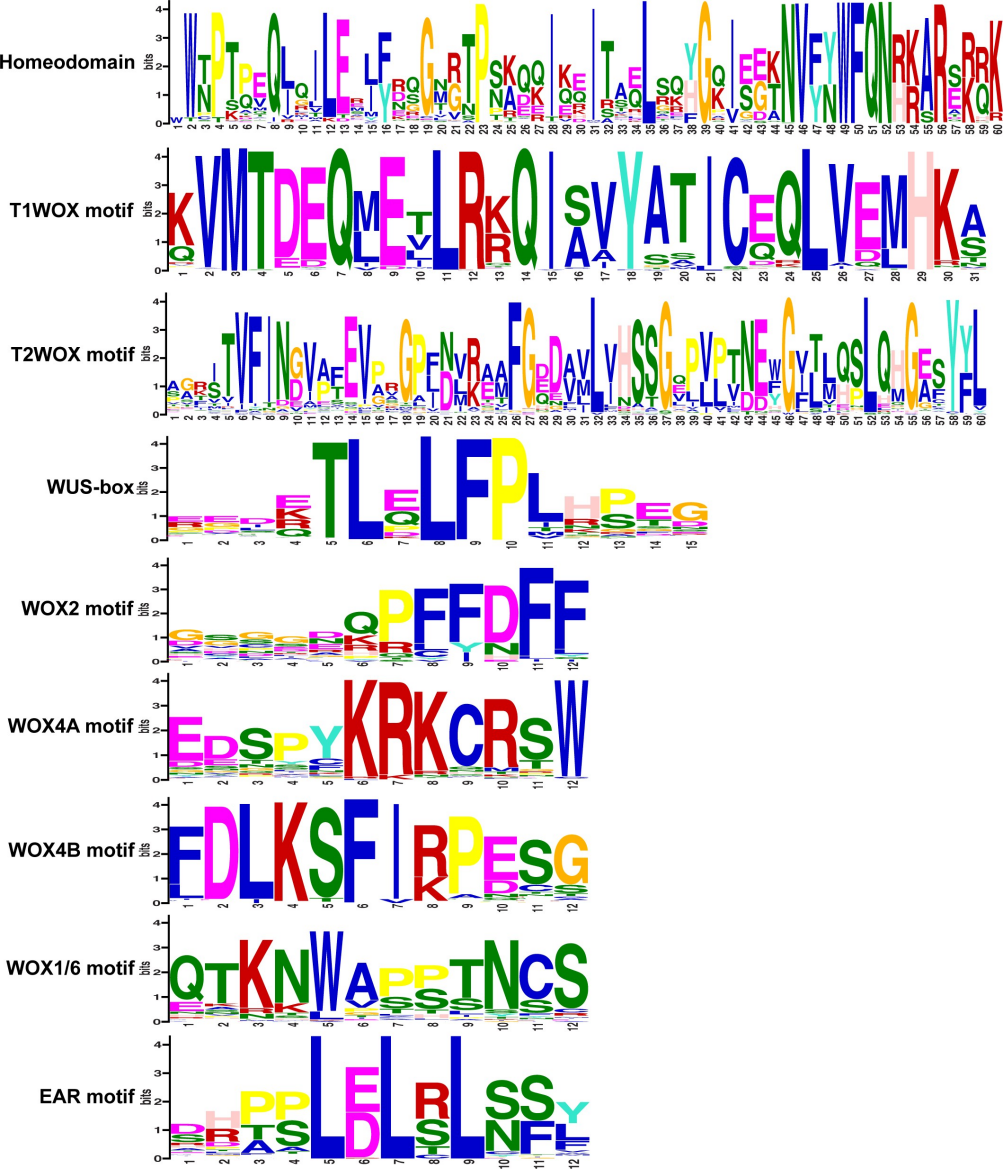
Conserved motifs in the WOX proteins. Amino acid motifs identified using MEME, with E-value cutoff smaller than 1e-5. The relative font size for each residue indicates sequence conservation, with a larger font representing higher conservation.

In the N-terminal domain of the T1WOX proteins, the T1WOX motif, which was previously referred to as WOX13 MOG [45], is highly conserved but is absent from the other types of WOX proteins (Fig 2). SynFind analysis (S3 Table) shows synteny among *AtWOX10*, *AtWOX13*, and *AtWOX14*.

The monophyly of the T2WOX superclade, which comprises the WOX8/9 and WOX11/12 clades, is recovered with BS support of 88 (S2 Fig). Downstream of the HD, the T2WOX proteins share the superclade-specific 60-amino-acid T2WOX motif (Fig 2), which comprises motifs previously named VFIN WOX8 MOG and LQxG WOX8 MOG [45]. This superclade comprises only proteins from seed plants. This result differs from previous studies in which some lycophyte and fern WOX homologs were clustered with the WOX8/9 and WOX11/12 clades [13, 15, 47, 52–54, 93]. However, those lycophyte and fern WOX proteins, including CrWOXA and CrWOXB of *Ceratopteris richardii*, do not contain the T2WOX motif, consistent with our result (see also below). These non-seed-plant WOX proteins contain the NVFYWFQNR motif in the HD, however, the NVFYWFQNR motif is also present is several T3WOX proteins (e.g., GbWOX3A of *Ginkgo biloba*, GgWOX2A and GgWOX2B of *Gnetum gnemon*, Migut.N02641 of *Mimulus gutatus*, cassava4.1 021403m of *Manihot esculenta*). On the other hand, we found no syntenic region of the T2WOX genes in the genomes of *A*. *filiculoides* and *S*. *moellendorffii*. These results suggest that the *T2WOX* genes may be lost in lycophytes and ferns. Synteny analysis also finds synteny between *AtWOX8* and *AtWOX9*, as well as that between *AtWOX11* and *AtWOX12*.

The moderately supported (BS 75) T3WOX proteins constitute a monophyletic superclade of (1) a well-supported (BS 100) clade of four lycophyte T3WOX proteins, (2) a weakly supported (BS 60) clade of 10 fern T3WOX proteins, and (3) a strongly-supported (BS 91) clade that includes major T3WOX clades of seed plant proteins and three fern homologs (S3 Fig). The phylogeny confirms that the T3WOX at least predates vascular plants (i.e., lycophytes, ferns, and seed plants). The T3WOX proteins generally possess a common sequence signature termed the WUS-box (Fig 2). However, the lycophyte T3WOX proteins, the weakly supported fern T3WOX clade members, AtWOX7, and XTZP-0091187 of *Araucaria rulei* lack this motif. The WOX2 clade has low support (BS 21), but shares the WOX2 motif in the C-terminal (Fig 2). The angiosperm WOX3 proteins form a monophyletic group with a WOX3 homolog from *G*. *gnemon* and two *G*. *biloba* WOX3 members with moderate BS support of 78 (S4 Fig). It is worth noting that GgWOX2A and GgWOX2B of *G*. *gnemon*, which were previously nested within the WOX2 clade [12, 13], are in the WOX3 clade, although with low support (BS 1). Neither GgWOX2A or GgWOX2B contains the WOX2 motif. *CrWUL* of *C*. *richardii* has previously been reconstructed as falling among polytomous lineages of the modern clade [13, 15, 49, 54, 55], along with two Azolla T3WOX proteins (Azfi_s0343.g065738 and Azfi_s0051.g031311), are also clustered with GgWOX2A and GgWOX2B. Despite little BS support for the WOX1/6 monophyletic clade (S5 Fig), most WOX1/6 proteins maintain the conserved WOX1/6 motif between the HD and the WUS box (Fig 2). Consistent with previous literature [12, 22, 49], no *WOX1/6* gene was found in the monocot genomes or transcriptomes sampled in this study. No syntelog of *AtWOX1* or *AtWOX6* was discovered by SynFind in the monocot genomes of *Anana comosus*, *Musa acuminata*, *Oryza sativa*, *Phalaenopsis equestris*, *Phoenix dactylifera*, *Triticum aestivum* and *Zea mays*. However, one genomic region (pos 29212359 on contig CM000126) sytenic to *AtWOX6* was found in rice. These lines of evidence suggest that the MRCA of monocots may have had a *WOX6* coding sequence but lost it before the divergence of extant monocot orders. Within the weakly supported the WOX5/7 clade (BS 52; S6 Fig), the monophyly of the angiosperm WOX5/7 proteins was recovered with strong support (BS 99). As sister to the WOX5/7 clade, the WUS proteins from seed plants constitute a monophyletic lineage with moderate support (BS 73). Among the seed plant WUS proteins, the angiosperm members comprise a monophyletic group with also moderate support (BS 74). The EAR motif [57] is recovered in the C-termini of all members of both WOX5/7 and WUS clades, except in AtWOX7 [47]. The EAR motif is encoded as L[DE]LRLS in the WOX5/7 clade members, while in the WUS clade proteins it is L[DE]L[ST]LN. The WOX4 clade has modest support (BS 88; S7 Fig). The gymnosperm WOX proteins are paraphyletic with the angiosperm WOX4 proteins nested in it (BS 82). Among the angiosperm WOX4 proteins, monocot members form a monophyletic clade, which is sister to all the other angiosperm WOX4 homologs. Most WOX4 proteins share the conserved WOX4 motif, which is upstream of the HD (Fig 2).

### Auxin Response Elements and B-ARR-6-BA motifs in the *WOX* promoters are deeply conserved in plants

**Fig 3:**
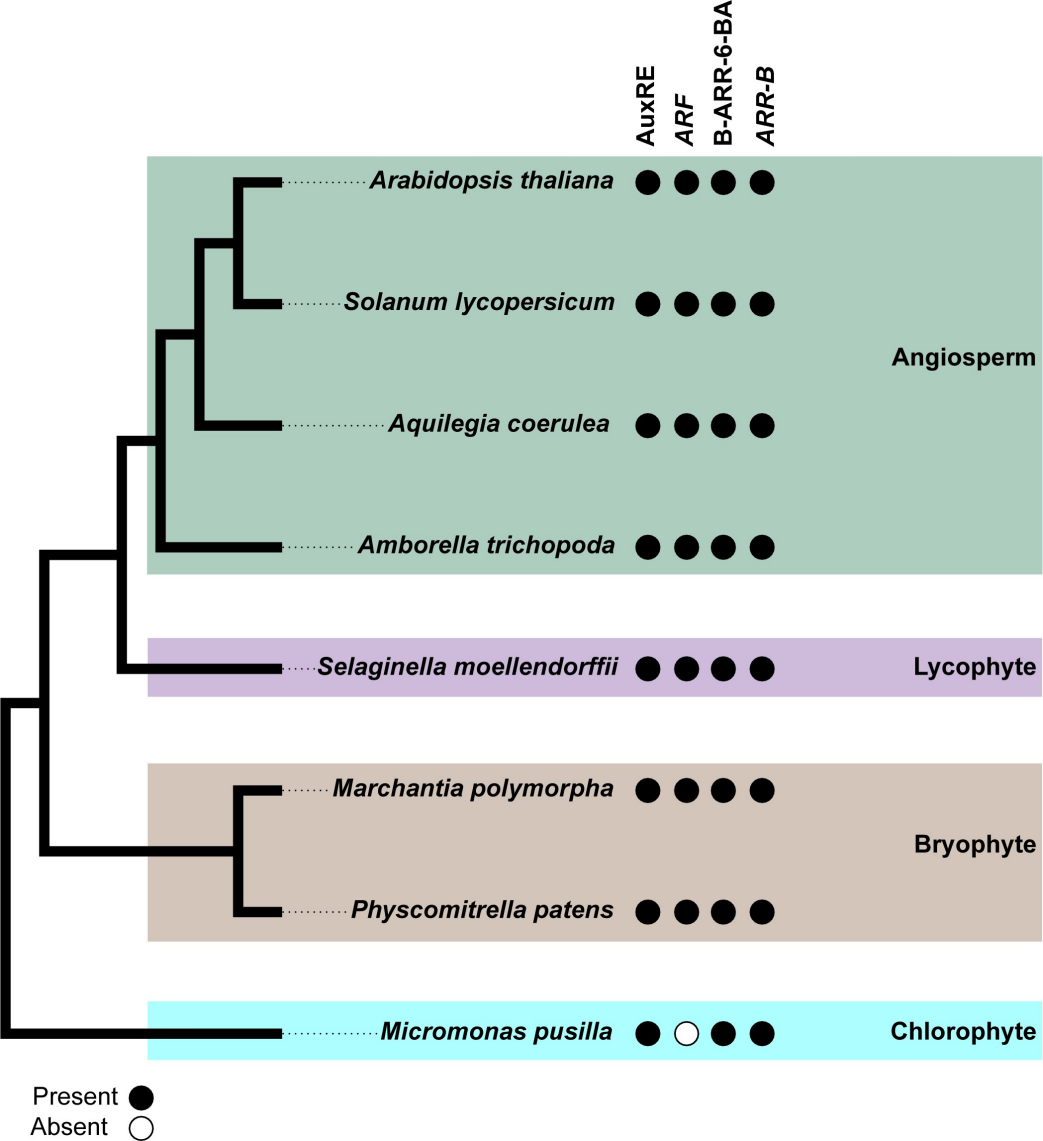
Conservation of the *WOX* regulation by the *ARF* and *ARR-B* genes in plants. Sequences 1.5kp upstream of the *WOX* genes in representative taxa were scanned using PlantPAN 2.0 for the presence of the AuxRE and B-ARR-6-BA motifs. Coding sequences of Arabidopsis *ARF* and *ARR-B* genes were used as queries to BLAST against genomes of representative taxa for the presence of their homologous genes. Closed circles depict the presence of the AuxRE or B-ARR-6-BA motif in the 1.5kp upstream of the *WOX* genes or that of the *ARF* or *ARR-B* genes in the representative genomes. Branches are boxed according to taxonomic affiliation: green for angiosperms; purple, lycophytes; brown, bryophytes; cyan, chlorophytes.

In order to elucidate how ancient the regulation of *WOX* genes by auxin and cytokinin may be in land plants, 1.5kb of sequence upstream of the transcriptional start sites of select *WOX* loci were obtained from Phytozome v12 for identification of known AuxRE and B-ARR-6-BA sequences (S4 Table). AuxREs and B-ARR-6-BA were found in the *WOX* promoters of all selected taxa. We also identified *ARR-B* homologs in all selected plant genomes but *ARF* homologs were restricted to land plant genomes (Fig 3).

## Discussion

### Advantages and remaining challenges of the current pipeline

To better understand the evolution of the developmentally critical *WOX* gene family, a large number of sequences spanning the majority of plant orders was employed for phylogenetic reconstruction and comparative analyses. Previous phylogenetic analyses of the *WOX* gene family were often inferred from datasets using only the HD, as sequences outside the HD are highly divergent, or using full-length sequences but with manual adjustment. Without efficient automatized alignment using robust software, it was difficult for the limited characters in those datasets to provide high phylogenetic resolution. By using PASTA, which performs better than other alignment softwares [94], the alignment strategy adopted in this study allowed the inclusion of additional 85 amino acids outside of the HD and improved the accuracy of the WOX protein phylogeny. Nevertheless, addressing the following challenges would likely contribute to finer resolution within the T1WOX and T3WOX superclades. First, most sequenced transcriptomes of the 1KP project were extracted from shoots or leaves, so the *WOX* genes expressed in other organs could have been missed. Second, transcripts from transcriptomes are often fragmentary. Third, the stringent filtering strategy for our alignment may have removed diagnostic characters for specific protein lineages. Development of an alignment pipelines that can better handle fragmentary sequences, as well as the addition of more sequenced genomes, will improve the robustness of phylogenetic inference.

### All WOX superclades are ancient

The WOX phylogeny inferred here reveals deep duplications that gave rise to three distinct superclades. The absence of the *WOX* genes in Rhodophyta supports the hypothesis that the *WOX* gene family is Viridiplantae-specific [95]. A gene duplication prior to the common ancestor of the Viridiplantae may have contributed to the establishment of two clades of chlorophytic WOX proteins: the T1WOX superclade, and the ancestor of the T2WOX + T3WOX superclades. No *T2WOX* or *T3WOX* loci are found in the genomes or transcriptomes of the sampled bryophytes, nor was their syntelog or syntenic proxy in the genomes of *Marchantia* or *Physcomitrella*. Syntenic analysis with sequenced genomes of hornworts could elucidate whether the MRCA of all non-vascular land plants lost the ancestor of the T2WOX + *T3WOX* genes. Most importantly, the *T1WOX* and *T2WOX* + *T3WOX* gene lineages are equally ancient and it is not evolutionarily accurate to consider the *T1WOX* lineage more “ancient” than the other two, or the functions of its members to be necessarily more ancestral. For this reason, we have used a different nomenclature than previous publications.

### Whole-genome duplications may contribute to divergence and expansion of the WOX proteins

**Fig 4:**
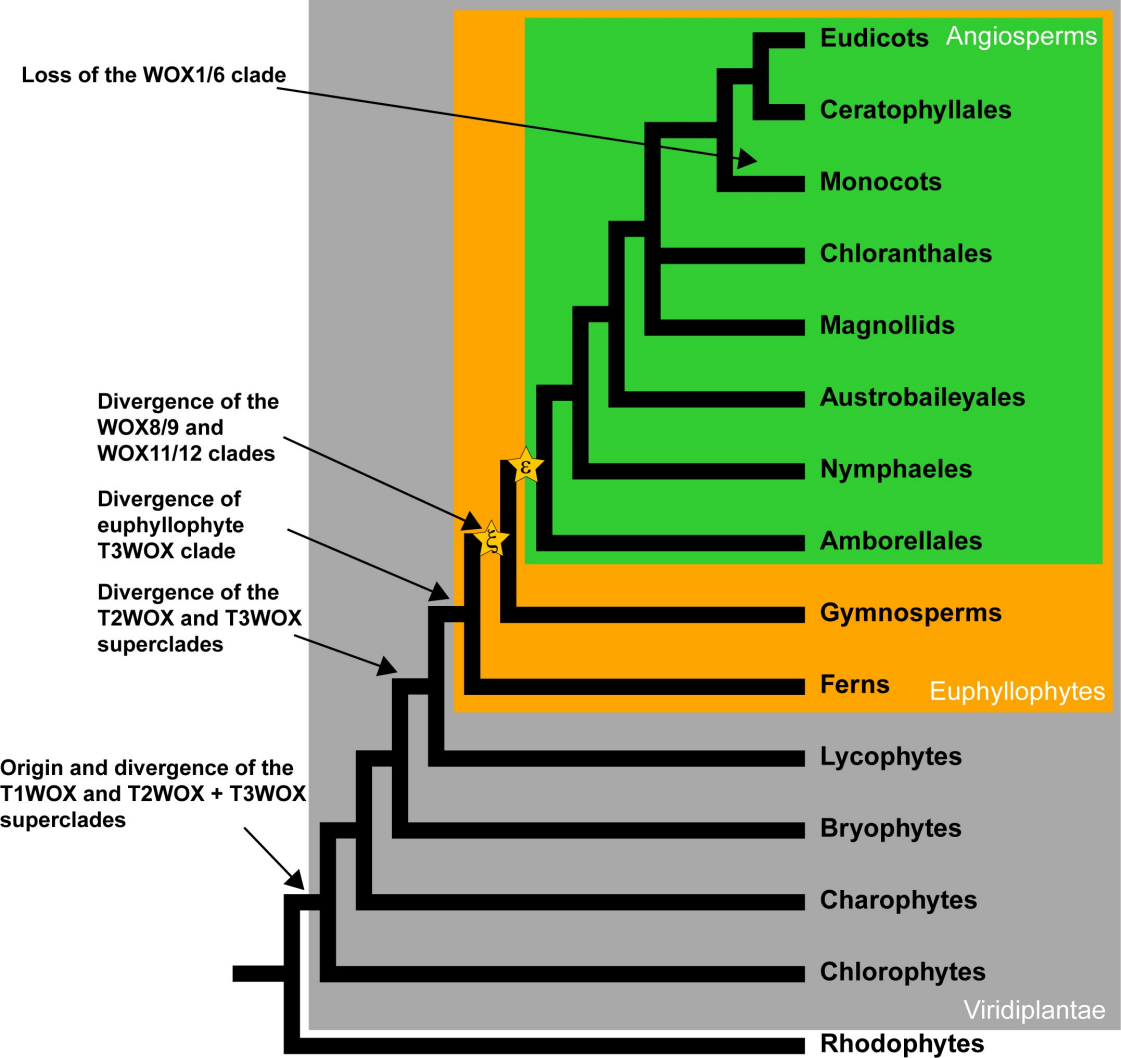
Major diversification events of the *WOX* family in plants. The simplified land plant phylogeny is plotted according to Ruhfel et al. [102] and Chase et al. [103]. The ε andζWGD events are marked in yellow stars. Angiosperms are boxed in green, euphyllophytes in orange, and the Viridiplantae in grey.

The WOX phylogeny inferred here reveal several gene duplications coinciding ancient WGD events (Fig 4). For instance, theζWGD event, which occurred before the divergence of seed plants [96, 97], appears to correspond with the gene duplication and subsequent diversification of the WOX8/9 and WOX 11/12 clades. The respective synteny between *AtWOX8* and *AtWOX9*, as well as *AtWOX11* and *AtWOX12* suggests gene duplication of these loci by another WGD. The WOX phylogeny also presents a major radiation of the T3WOX clades at the base of euphyllophytes (i.e., ferns and seed plants). Polyploidy is prevalent in extant lycophytes and ferns, including a paleopolyploidization in the most basal euphyllophytic lineage to Equisetidae (approximately 92.42 MYA) and a WGD near the base of the Polypodiidae (approximately 178 MYA) [98, 99]. However, no WGD has been discovered in the stem group of vascular plants to date [100]. In order to decipher whether WGD contributes to the divergence of the WOX clades at the base of vascular plants, additional sequenced genomes from lycophytes (e.g., Lycopodiaceae and Isoetaceae) and eusporangiate ferns (e.g., Equisetidae, Ophioglossidae, Marattiidae, etc.) are necessary [101].

### Divergence of the WUS and WOX5/7 clades may predate the emergence of euphyllophytes

In this study, our result recovers the sister relationship between the WUS and WOX5/7 clades and suggests that the divergence of the WUS and WOX5/7 clades may predate the emergence of euphyllophytes. We also show strong support for the monophyly of the angiosperm WOX5/7 proteins, better support for the monophyletic group of angiosperm WUS proteins than previous studies [13, 15, 55], and moderate support for the monophyly of the seed plant WUS proteins, which is lower than that of some previous studies inferred from smaller WOX datasets [12, 54, 60]. The WUS and WOX5/7 clades are the best-studied clades among the WOX clades. *AtWUS* mediates the stem-cell niche in the organizing center (OC) of the SAM through the WUS-CLAVATA3 (CLV3) feedback circuit, which requires the intercellular movement of AtWUS [9–11, 104]. Similarly, AtWOX5 regulates the stem-cell niche of the QC in the RAM via a feedback loop with auxin-related response factors [105]. AtWUS and AtWOX5 function non-cell-autonomously and are biochemically interchangeable for stem cell maintenance of the SAM and RAM [21]. AtWUS and AtWOX5 interact with the BREAST CANCER ASSOCIATED RING 1 (BARD1)/REPRESSOR OF WUSCHEL1 (ROW1) and HAIRY MERISTEM (HAM) proteins in the SAM and RAM, respectively, to regulate the stem-cell niche [106–109].

The evolutionary trajectory underlying the functional divergence of the WUS and WOX5/7 clades remains unknown. Previous phylogenetic studies suggested that the WUS and WOX5/7 clades diverged prior the crown group of seed plants and after emergence of euphyllophytes [12, 13, 15, 47, 49, 53–55, 93]. Gene expression patterns of the *WUS* and *WOX5*/*7* genes have been characterized in a limited number of euphyllophyte taxa. Expression of *Ginkgo GbWUS*, *Gnetum GgWUS*, *Pinus PsWOX5* and *Picea PaWOX5* were detected in both shoot and root, while *GbWUS* was also expressed during reproductive organ development [12, 52]. In *Pinus pinaster*, *PpWUS* expression was detected in the shoot only, while high *PpWOX5* expression was observed in the root with low expression levels in the shoot [55]. In the basal angiosperm *Nymphea*, *NjWUS* was also shown to be expressed in the shoot, but not in the root. Heterologous expression of *GgWUS* driven by the promoter of *AtWUS* in Arabidopsis increased stem cell population in the SAM, inflorescence meristem, and floral meristem [12]. Employing a similar transgenic approach, another study showed that GbWUS, PaWUS, PsWUS, PaWOX5, and PsWOX5 can all complement AtWUS and AtWOX5 function in Arabidopsis, including the capability to regulate stem-cell maintenance and to move from cell to cell [15]. In the same analysis, *CrWUL* of *C*. *richardii*, which was thought to be sister to the WUS and WOX5/7 clades with gene expression in root tips and gametophytes but not in the shoot apical cell [13], could not rescue the *AtWUS* or *AtWOX5* mutants when driven by the *AtWUS* or *AtWOX5* promoter. However, when driven by *AtCLV* promoter, *CrWUL* was shown to have the capability to maintain stem-cell niche in SAM, demonstrating a lack of intercellular mobility in Arabidopsis. One possible explanation for this result is that CrWUL may not have the capacity of intercellular movement in its endogenous context. Alternatively, it is possible that the inteceullar mobility of CrWUL was prohibited in Arabidopsis because of a mismatch in the protein transport machinery.

The genomic context and gene function may have diversified since the MRCA of these taxa, and thus heterologous expression of a gene may not reflect actual gene function in the endogenous genomic context [110]. Ancestors of Arabidopsis and *Ceratopteris* diverged approximately 411 million years ago (MYA), while that of Arabidopsis and gymnosperms (e.g., *Ginkgo*, *Gnetum*, and *Pinus*) diverged approximately 330 MYA, and that of Arabidopsis and *Nymphea* diverged approximately 139 MYA [111, 112]. These lines of evidence, as well as the WOX phylogeny inferred here, suggests that the biochemical capacity to maintain stem-cell niche may be synapomorphic for the crown group of euphyllophytes and that the subfunctionalization of either the *AtWUS* homologs in shoots and flowers or the *AtWOX5/7* homologs in roots may have occurred after the duplication in the lineage leading to seed plants.

In addition, AtWOX11 and AtWOX12 proteins directly bind to the promoters of *AtWOX5* and *AtWOX7* to activate their expression [113], but the evolutionary conservation of the regulation of the T3WOX genes by the T2WOX genes remains elusive. Functional analyses of the T3WOX genes in ferns and lycophytes, as well as *Selaginella* SmWOXII, which is positioned as sister to all the other T3WOX clades are critical to understanding (1) whether the cell-to-cell mobility occurs in the fern lineage, (2) the origin and functional divergence of the WUS and WOX5/7 clades, and (3) evolution of the regulation of the T3WOX genes by the T2WOX genes.

### Regulation of the *WOX* genes by auxin and cytokinin signaling pathways may exist early in plant evolution

Auxin and cytokinin are prominent phytohormones that regulate various plant developmental processes [114]. In particular, the auxin and cytokinin signaling pathways control meristem development, in which the *WOX* genes also play critical roles [115–117]. Our survey of the *WOX* gene promoters from representative plants reveals the prevalence of the AuxREs across the Viridiplantae. This result is consistent with previous studies [118–121]. Although AUX/IAA family members are absent from green algae [122] and *ARF* genes are not found in chlorophytes [121], our result suggests that the capability of *WOX* genes to be regulated by auxin signaling pathway was established early in Viridiplantae, although most components of the pathway did not evolve until the emergence of the land plants [121–123]. Similarly, the presence of both the *ARR-B* genes and the B-ARR-6-BA motif in the *WOX* promoters across plant lineages is consistent with deep conservation of *WOX* regulation by the cytokinin signaling pathway. Alternatively, it could be possible that the early-divering plant lineages may use another B-ARR motif or AuxRE that is different from what is included in the PlantPAN matrix based on angiosperm collections. We can not rule out the possibility that the detection of these binding sites is not functionally relevant and just due to the likelihood of finding a given short sequence in a large DNA string. Genetic analysis is necessary to confirm whether the ARF and ARR-B proteins actually regulate the *WOX* genes in nonvascular land plants.

The *WOX* genes are indeed both up- and down-stream of the auxin and cytokinine pathways in meristem development [63]. For instance, WUS protects apical stem cells from differentiation by restricting the auxin signaling pathway via regulation of histone de-acetylation [124]. In addition, *WUS* directly represses the type-A *ARR5*, *ARR6*, *ARR7*, and *ARR15* genes, which function in the negative feedback loop of cytokinin signaling [125]. However, functional analysis is necessary to determine whether the other *WOX* genes reciprocally regulate the auxin or cytokinin signaling pathways.

## Conclusions

In conclusion, our phylogenetic reconstruction based on 1039 protein sequences from 267 species across the Viridiplantae suggests three ancient WOX superclades: T1WOX, T2WOX, and T3WOX. Divergence of the T1WOX and T2WOX superclades may predate diversification of vascular plants. Our analysis of the *WOX* promoters also suggests that the capacity of the WOX genes to be regulated by the auxin and cytokinin signaling pathways could be deeply conserved in the Viridiplantae. As expansion of the *WOX* gene family and the gene families involved in the auxin and cytokinin signaling pathways have been correlated with morphological innovations during plant radiation [12, 46, 47, 50, 54, 120], robust phylogenies of the *WOX* genes and the auxin and cytokinin pathway components may provide insight for experimental design to decipher how and when the these genes were recruited into the gene regulatory network underlying developmental and morphological complexity during plant evolution.

## Supporting information

S1 Fig Maximum-likelihood (ML) phylogeny of the WOX protein family based on WOXaa_g. The ML phylogeny was reconstructed by RAxML 8.2.4 with 1,000 bootstrap replicates. Quadruple branch width indicates BS support equal or greater than 50. BS values are shown at key branches. Branches are colored according to taxonomic affiliation: green for angiosperms; red, gymnosperms; blue, ferns; purple, lycophytes; brown, bryophytes; yellow, charophytes; cyan, chlorophytes. Clades are shaded in a gradient of gray. Major superclades are marked with vertical lines. The scale is amino acid substitution rate of 0.5.
(TIF)

S2 Fig Maximum likelihood phylogeny of the WOX protein family. **Portion of tree showing the T2WOX proteins.** The ML phylogeny is reconstructed by RAxML 8.2.4 with 1,000 bootstrap replicates. Internal nodes with bootstrap values equal to and more than 50 are marked. Arabidopsis T2WOX proteins are indicated by arrowheads. Branches are colored according to taxonomic affiliation: green for angiosperms and red for gymnosperms. The scale is an amino acid substitution rate of 0.5.
(TIF)

S3 Fig Maximum likelihood phylogeny of the WOX protein family. **Portion of tree showing the basal T3WOX proteins.** The ML phylogeny is reconstructed by RAxML 8.2.4 with 1,000 bootstrap replicates. Internal nodes with bootstrap values equal to and more than 50 are marked. Arabidopsis WOX2 is indicated by an arrowhead. Branches are colored according to taxonomic affiliation: green for angiosperms and red for gymnosperms. The scale is an amino acid substitution rate of 0.5.
(TIF)

S4 Fig Maximum likelihood phylogeny of the WOX protein family. **Portion of tree showing the WOX3 proteins.** The ML phylogeny is reconstructed by RAxML 8.2.4 with 1,000 bootstrap replicates. Internal nodes with bootstrap values equal to and more than 50 are marked. Arabidopsis WOX4 is indicated by an arrowhead. Branches are colored according to taxonomic affiliation: green for angiosperms and red for gymnosperms. The scale is an amino acid substitution rate of 0.5.
(TIF)

S5 Fig Maximum likelihood phylogeny of the WOX protein family. **Portion of tree showing the WOX1/6 proteins.** The ML phylogeny is reconstructed by RAxML 8.2.4 with 1,000 bootstrap replicates. Internal nodes with bootstrap values equal to and more than 50 are marked. Arabidopsis WOX1 and WOX6 are indicated by arrowheads. Branches are colored according to taxonomic affiliation: green for angiosperms and red for gymnosperms. The scale is an amino acid substitution rate of 0.5.
(TIF)

S6 Fig Maximum likelihood phylogeny of the WOX protein family. **Portion of tree showing the WUS and WOX5/7 proteins.** The ML phylogeny is reconstructed by RAxML 8.2.4 with 1,000 bootstrap replicates. Internal nodes with bootstrap values equal to and more than 50 are marked. Arabidopsis WOX proteins are indicated by arrowheads. Branches are colored according to taxonomic affiliation: blue for ferns, green for angiosperms and red for gymnosperms. The scale is an amino acid substitution rate of 0.5.
(TIF)

S7 Fig Maximum likelihood phylogeny of the WOX protein family. **Portion of tree showing the WOX4 proteins.** The ML phylogeny is reconstructed by RAxML 8.2.4 with 1,000 bootstrap replicates. Internal nodes with bootstrap values equal to and more than 50 are marked. Arabidopsis WOX proteins are indicated by arrowheads. Branches are colored according to taxonomic affiliation: blue for ferns, green for angiosperms and red for gymnosperms. The scale is an amino acid substitution rate of 0.5.
(TIF)

S1 Table Proteins included in the dataset WOXaa. (DOCX)

S2 Table Arabidopsis *ARF* and *B-ARR* genes used as queries for BLAST search. (DOCX)

S3 Table Synteny analysis of Arabidopsis *WOX* genes. (XLSX)

S4 Table Predicted Auxin Response Elements and B-ARR-6-BA motif in promoters of plant *WOX* genes. (DOCX)

S1 File WOXaa. (PHY)

S2 File WOXaa_g. (PHY)
