# Supplementary figures and images for: Large-scale phylogenomic analysis suggests three ancient superclades of the WUSCHEL-RELATED HOMEOBOX transcription factor family in plants
Source: PLoS One. 2019 Oct 11;14(10):e0223521. doi: 10.1371/journal.pone.0223521 (PMC6788696; doi:10.1371/journal.pone.0223521)

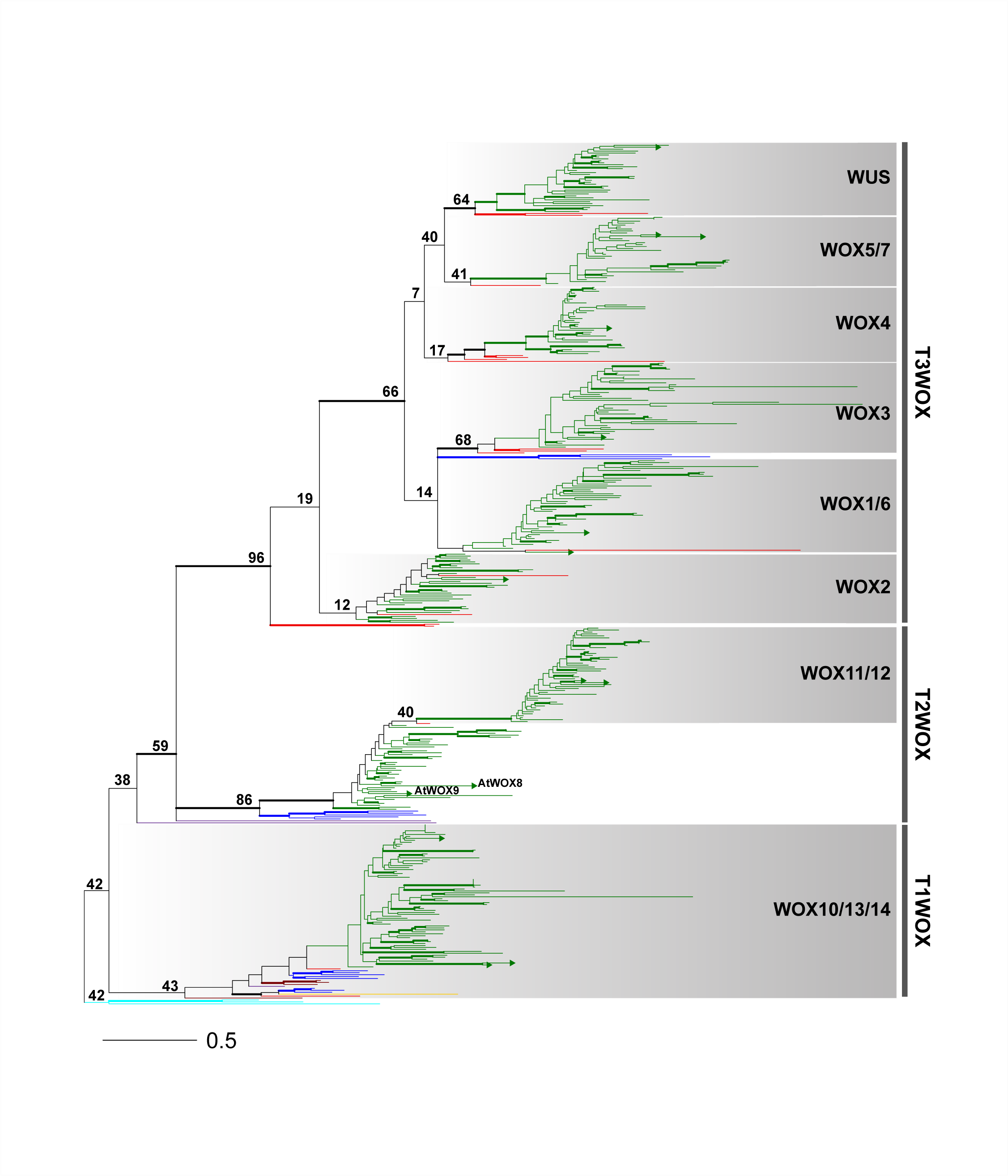

Supplement: S1 Fig — The ML phylogeny was reconstructed by RAxML 8.2.4 with 1,000 bootstrap replicates. Quadruple branch width indicates BS support equal or greater than 50. BS values are shown at key branches. Branches are colored according to taxonomic affiliation: green for angiosperms; red, gymnosperms; blue, ferns; purple, lycophytes; brown, bryophytes; yellow, charophytes; cyan, chlorophytes. Clades are shaded in a gradient of gray. Major superclades are marked with vertical lines. The scale is amino acid substitution rate of 0.5. (TIF) [file pone.0223521.s001.tif]

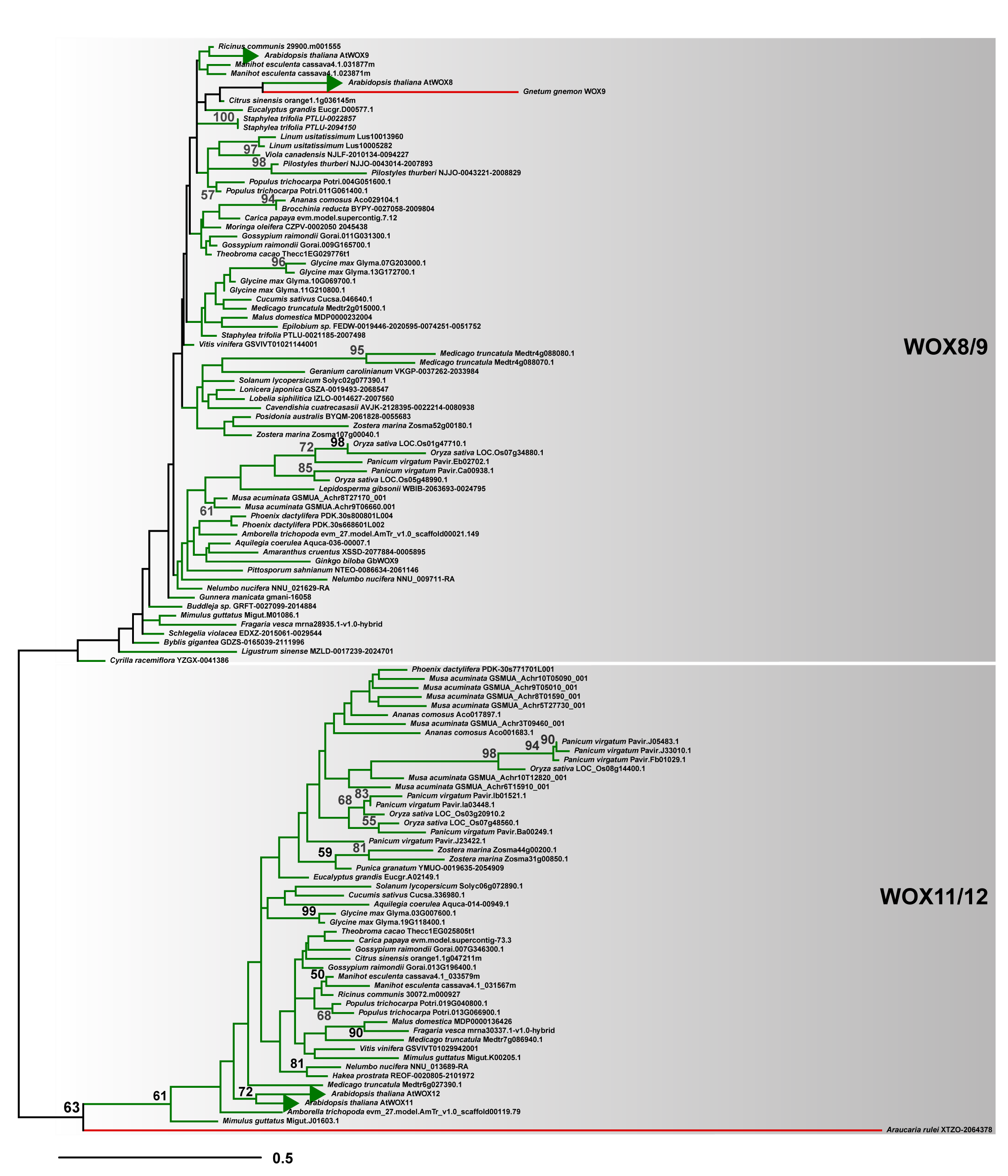

Supplement: S2 Fig — Portion of tree showing the T2WOX proteins. The ML phylogeny is reconstructed by RAxML 8.2.4 with 1,000 bootstrap replicates. Internal nodes with bootstrap values equal to and more than 50 are marked. Arabidopsis T2WOX proteins are indicated by arrowheads. Branches are colored according to taxonomic affiliation: green for angiosperms and red for gymnosperms. The scale is an amino acid substitution rate of 0.5. (TIF) [file pone.0223521.s002.tif]

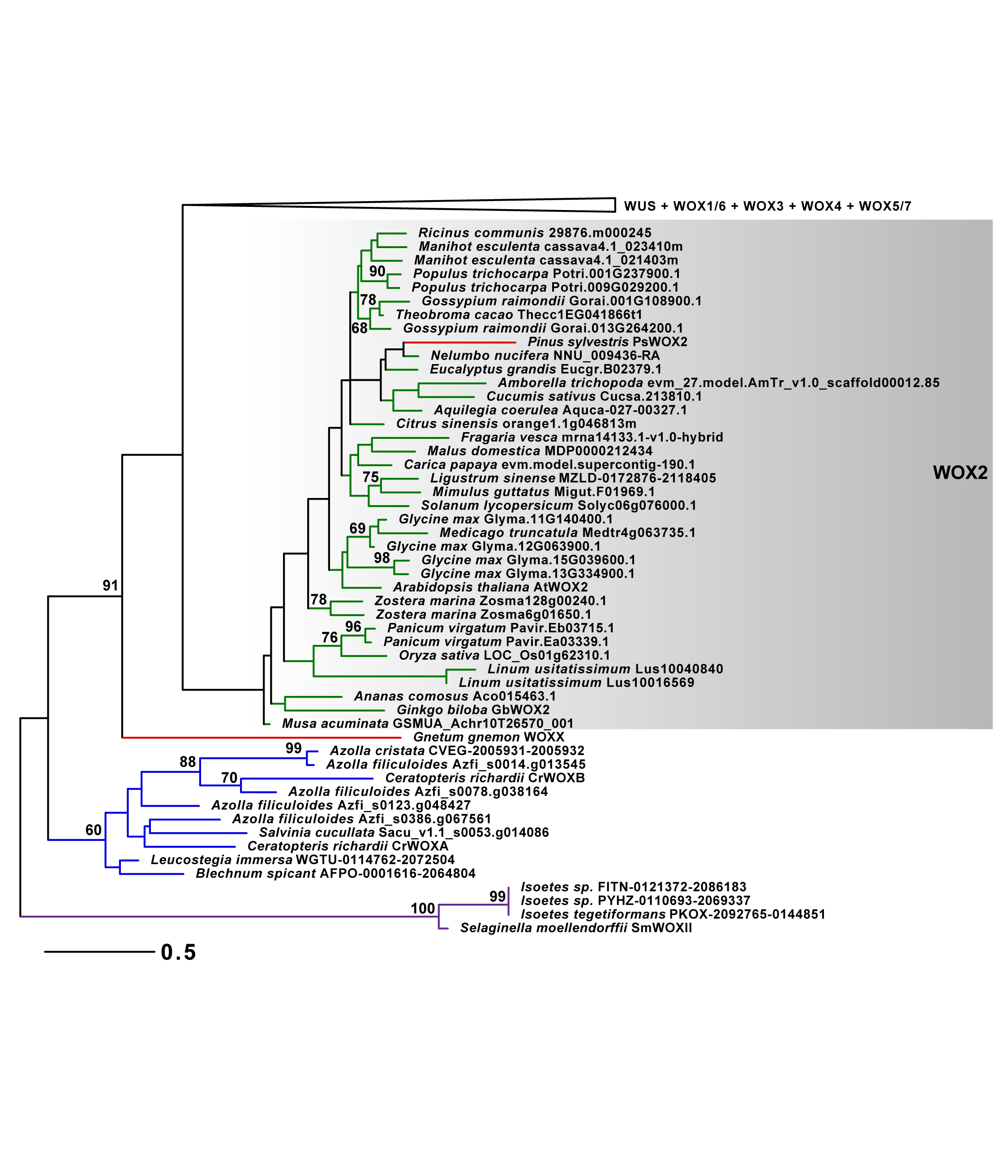

Supplement: S3 Fig — Portion of tree showing the basal T3WOX proteins. The ML phylogeny is reconstructed by RAxML 8.2.4 with 1,000 bootstrap replicates. Internal nodes with bootstrap values equal to and more than 50 are marked. Arabidopsis WOX2 is indicated by an arrowhead. Branches are colored according to taxonomic affiliation: green for angiosperms and red for gymnosperms. The scale is an amino acid substitution rate of 0.5. (TIF) [file pone.0223521.s003.tif]

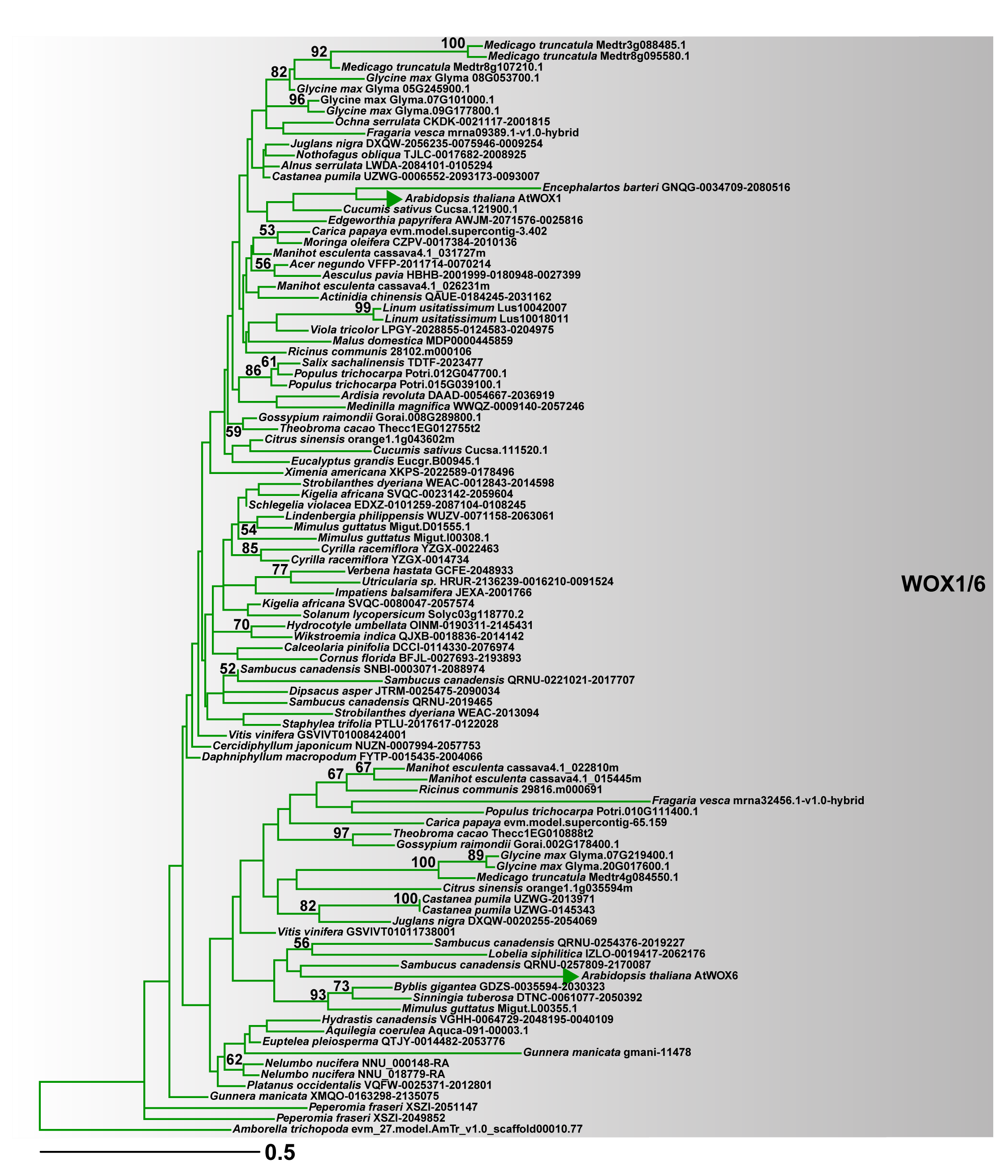

Supplement: S4 Fig — Portion of tree showing the WOX3 proteins. The ML phylogeny is reconstructed by RAxML 8.2.4 with 1,000 bootstrap replicates. Internal nodes with bootstrap values equal to and more than 50 are marked. Arabidopsis WOX4 is indicated by an arrowhead. Branches are colored according to taxonomic affiliation: green for angiosperms and red for gymnosperms. The scale is an amino acid substitution rate of 0.5. (TIF) [file pone.0223521.s004.tif]

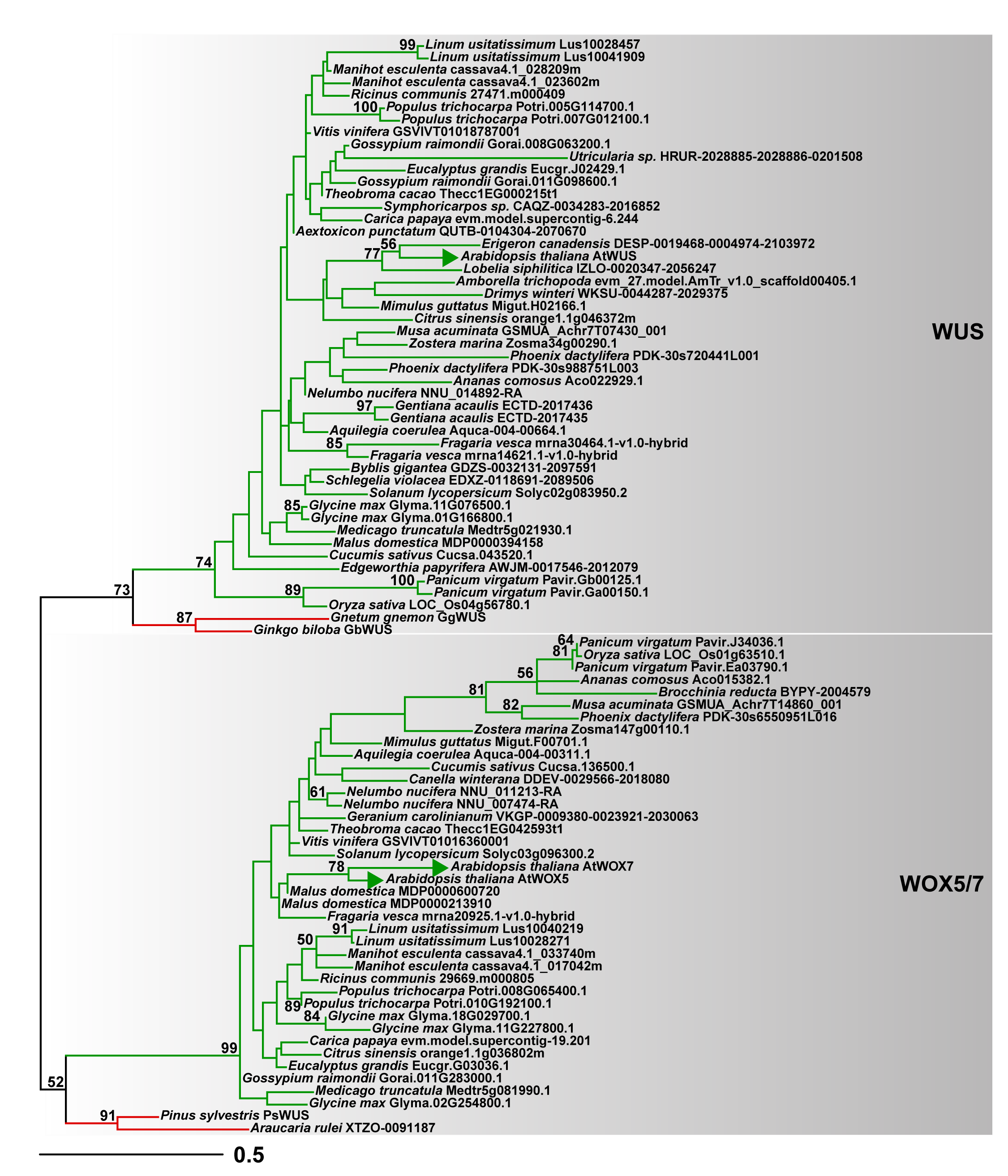

Supplement: S6 Fig — Portion of tree showing the WUS and WOX5/7 proteins. The ML phylogeny is reconstructed by RAxML 8.2.4 with 1,000 bootstrap replicates. Internal nodes with bootstrap values equal to and more than 50 are marked. Arabidopsis WOX proteins are indicated by arrowheads. Branches are colored according to taxonomic affiliation: blue for ferns, green for angiosperms and red for gymnosperms. The scale is an amino acid substitution rate of 0.5. (TIF) [file pone.0223521.s006.tif]

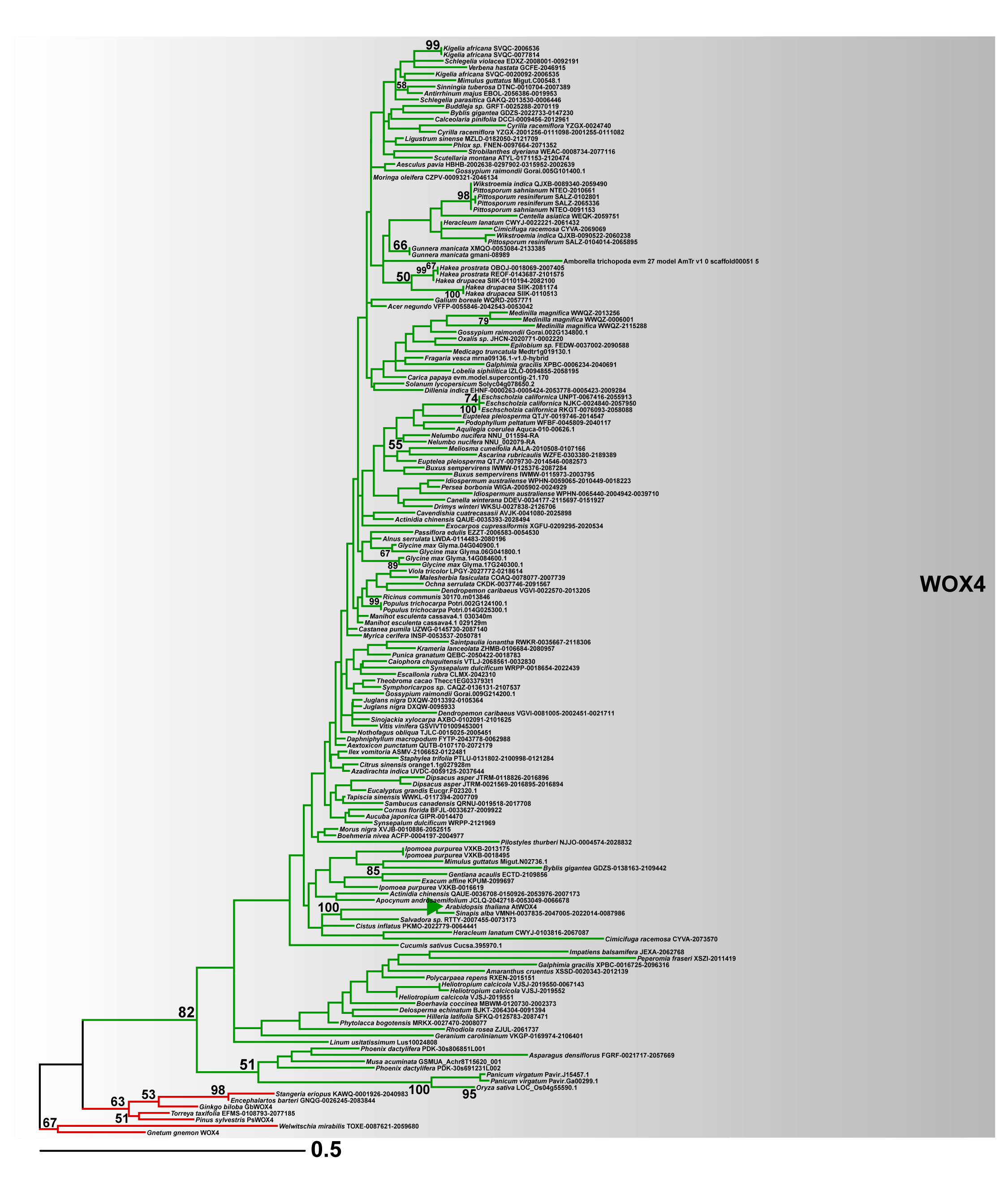

Supplement: S7 Fig — Portion of tree showing the WOX4 proteins. The ML phylogeny is reconstructed by RAxML 8.2.4 with 1,000 bootstrap replicates. Internal nodes with bootstrap values equal to and more than 50 are marked. Arabidopsis WOX proteins are indicated by arrowheads. Branches are colored according to taxonomic affiliation: blue for ferns, green for angiosperms and red for gymnosperms. The scale is an amino acid substitution rate of 0.5. (TIF) [file pone.0223521.s007.tif]
