# Supplementary material for: Large-scale phylogenomic analysis suggests three ancient superclades of the WUSCHEL-RELATED HOMEOBOX transcription factor family in plants
Source: PLoS One. 2019 Oct 11;14(10):e0223521. doi: 10.1371/journal.pone.0223521 (PMC6788696; doi:10.1371/journal.pone.0223521)
Supplement: S1 Table — (DOCX) [file pone.0223521.s008.docx]

**S1 Table. Proteins included in the dataset WOXaa.**

| Order | Family | Species | Protein | Source |
| --- | --- | --- | --- | --- |
| **Chlorophyta** |  |  |  |  |
| Mamiellales | Mamiellaceae | *Bathycoccus prasinos* | MCPK-0004814 | 1KP |
| Mamiellales | Mamiellaceae | *Ostreococcus lucimarinus* | OSTLU 27102 | Phytozome |
| Mamiellales | Mamiellaceae | *Micromonas pusilla* *CCMP1545* | MICPUCDRAFT_48125 | Phytozome |
| Mamiellales | Mamiellaceae | *Micromonas* *sp*. *RCC299* | MICPUN59370 | Phytozome |
| Pseudoscourfieldiales | Pycnococcaceae | *Nephroselmis olivacea* | MMKU-0055197-2046416-0059806 | 1KP |
| Ulotrichales | Ulotrichaceae | *Interfilum paradoxum* | FPCO-0043839-2029477-0039543 | 1KP |
| **Coleochaetophyta** |  |  |  |  |
| Coleochaetales | Coleochaetaceae | *Coleochaete irregularis* | QPDY-0041643-2005558-0042035 | 1KP |
| **Charophyta** |  |  |  |  |
| Klebsormidiales | Klebsormidiaceae | *Klebsormidium nitens* | GAQ80365 | *Klebsormidium nitens* genome project |
| Klebsormidiales | Klebsormidiaceae | *Klebsormidium subtile* | FQLP-2028874 | 1KP |
| Zygnematales | Mesotaeniaceae | *Netrium digitus* | FFGR-2013013 | 1KP |
| **Anthocerotophyta** |  |  |  |  |
| Dendrocerotales | Dendrocerotaceae | *Megaceros flagellaris* | UCRN-2001490-0053583-0010688-2001488-2001487 | 1KP |
| Dendrocerotales | Dendrocerotaceae | *Megaceros flagellaris* | UCRN-2012046-0084942 | 1KP |
| Dendrocerotales | Dendrocerotaceae | *Nothoceros aenigmaticus* | DXOU-2003567 | 1KP |
| Notothyladales | Notothyladaceae | *Phaeoceros carolinianus* | WCZB-0209918-2009602-2009603 | 1KP |
| Notothyladales | Notothyladaceae | *Phaeoceros carolinianus* | WCZB-0213315-2116929 | 1KP |
| **Bryophyta** |  |  |  |  |
| Andreaeales | Andreaeaceae | *Andreaea rupestris* | WOGB-0140762-2095435 | 1KP |
| Bryales | Aulacomniaceae | *Aulacomnium heterostichum* | WNGH-0135763-2013485-0139367 | 1KP |
| Buxbaumiales | Buxbaumiaceae | *Buxbaumia aphylla* | HRWG-0105517-2013944 | 1KP |
| Diphysiales | Diphysciaceae | *Diphyscium foliosum* | AWOI-0001959-2069721 | 1KP |
| Diphysiales | Diphysciaceae | *Diphyscium foliosum* | AWOI-0103272-2013964 | 1KP |
| Dicranales | Ditrichaceae | *Ceratodon purpureus* | FFPD-2003633-0046026 | 1KP |

**S1 Table.** (Continued)

| Order | Family | Species | Protein | Source |
| --- | --- | --- | --- | --- |
| Dicranales | Ditrichaceae | *Ceratodon purpureus* | FFPD-2003634-0046043 | 1KP |
| Dicranales | Leucobryaceae | *Leucobryum glaucum* | RGKI-0078917-2000574 | 1KP |
| Encalyptales | Encalyptaceae | *Encalypta streptocarpa* | KEFD-2060667-0078805 | 1KP |
| Funariales | Funariaceae | *Physcomitrella patens* | Phpat.009G053100.1 | Phytozome |
| Funariales | Funariaceae | *Physcomitrella patens* | Phpat.015G073700.1 | Phytozome |
| Funariales | Funariaceae | *Physcomitrella patens* | Phpat.026G031100.1 | Phytozome |
| Grimmiales | Scouleriaceae | *Scouleria aquatica* | BPSG-0019362-2088071 | 1KP |
| Grimmiales | Scouleriaceae | *Scouleria aquatica* | BPSG-2089442-0135393 | 1KP |
| Hedwigiales | Hedwigiaceae | *Hedwigia ciliata* | YWNF-0075529-2049816 | 1KP |
| Hypnales | Neckeraceae | *Neckera douglasii* | TMAJ-0290659-2019973 | 1KP |
| Hypnales | Thuidiaceae | *Anomodon attenuatus* | QMWB-2011221-0075309-2011222 | 1KP |
| Polytrichales | Polytrichaceae | *Atrichum angustatum* | ZTHV-0026492-2080744 | 1KP |
| Polytrichales | Polytrichaceae | *Atrichum angustatum* | ZTHV-0117900-2082872 | 1KP |
| Polytrichales | Polytrichaceae | *Atrichum angustatum* | ZTHV-0119666-2003285 | 1KP |
| Polytrichales | Polytrichaceae | *Polytrichum commune* | SZYG-0048654-2009900 | 1KP |
| Sphagnales | Sphagnaceae | *Sphagnum lescurii* | GOWD-1019526 | 1KP |
| Sphagnales | Sphagnaceae | *Sphagnum lescurii* | GOWD-1029695 | 1KP |
| Sphagnales | Sphagnaceae | *Sphagnum lescurii* | GOWD-1035332-2018894 | 1KP |
| Sphagnales | Sphagnaceae | *Sphagnum lescurii* | GOWD-1228193-2083093 | 1KP |
| Sphagnales | Sphagnaceae | *Sphagnum palustre* | RCBT-0286582-2007045-0153212 | 1KP |
| Sphagnales | Sphagnaceae | *Sphagnum palustre* | RCBT-2007046 | 1KP |
| Sphagnales | Sphagnaceae | *Sphagnum palustre* | RCBT-2027136-0298376 | 1KP |
| Sphagnales | Sphagnaceae | *Sphagnum palustre* | RCBT-2035662-0046765 | 1KP |
| Tetraphidales | Tetraphidaceae | *Tetraphis pellucida* | HVBQ-0170637-2126716 | 1KP |
| Tetraphidales | Tetraphidaceae | *Tetraphis pellucida* | HVBQ-0177106-2130119 | 1KP |
| **Marchantiophyta** |  |  |  |  |
| Jungermanniales | Plagiochilaceae | *Plagiochila asplenioides* | NWQC-2017476-0181727-0220504 | 1KP |
| Jungermanniales | Porellaceae | *Porella pinnata* | UUHD-2014718-0220256 | 1KP |

**S1 Table.** (Continued)

| Order | Family | Species | Protein | Source |
| --- | --- | --- | --- | --- |
| Jungermanniales | Ptilidiaceae | *Ptilidium pulcherrimum* | HPXA-2019172-0179776 | 1KP |
| Jungermanniales | Radulaceae | *Radula lindenbergiana* | BNCU-0129332-2087086 | 1KP |
| Jungermanniales | Scapaniaceae | *Barbilophozia barbata* | OFTV-0009581-2014361-2014360 | 1KP |
| Marchantiales | Marchantiaceae | *Marchantia polymorpha* | JPYU-2008315-0046859 | 1KP |
| Marchantiales | Marchantiaceae | *Marchantia polymorpha* | Mapoly0014s0060.2 | Phytozome |
| **Lycopodiopphyta** |  |  |  |  |
| Isoetales | Isoetaceae | *Isoetes* sp. | FITN-0029705-2086852 | 1KP |
| Isoetales | Isoetaceae | *Isoetes* sp. | FITN-0077962-2087669 | 1KP |
| Isoetales | Isoetaceae | *Isoetes* sp. | FITN-0121372-2086183 | 1KP |
| Isoetales | Isoetaceae | *Isoetes* sp. | FITN-0124355-2089379 | 1KP |
| Isoetales | Isoetaceae | *Isoetes* sp. | PYHZ-0110693-2069337 | 1KP |
| Isoetales | Isoetaceae | *Isoetes* sp. | PYHZ-0114592-2073427 | 1KP |
| Isoetales | Isoetaceae | *Isoetes* sp. | PYHZ-2010651-0078682 | 1KP |
| Isoetales | Isoetaceae | *Isoetes* sp. | PYHZ-2012649-0092746 | 1KP |
| Isoetales | Isoetaceae | *Isoetes tegetiformans* | PKOX-0008832-2097834 | 1KP |
| Isoetales | Isoetaceae | *Isoetes tegetiformans* | PKOX-0157499-2011854 | 1KP |
| Isoetales | Isoetaceae | *Isoetes tegetiformans* | PKOX-0158509-2012248 | 1KP |
| Isoetales | Isoetaceae | *Isoetes tegetiformans* | PKOX-2092765-0144851 | 1KP |
| Lycopodiales | Huperziaceae | *Huperzia squarrosa* | GAON-2054303-0019576 | 1KP |
| Lycopodiales | Huperziaceae | *Phylloglossum drummondii* | ZZEI-0035580-2015876 | 1KP |
| Lycopodiales | Huperziaceae | *Phylloglossum drummondii* | ZZEI-0157178-2123380 | 1KP |
| Lycopodiales | Lycopodiaceae | *Diphasiastrum digitatum* | WAFT-0026455-2065089 | 1KP |
| Selaginellales | Selaginellaceae | *Selaginella moellendorffii* | 417553 | Phytozome |
| Selaginellales | Selaginellaceae | *Selaginella moellendorffii* | 4561 | Phytozome |
| Selaginellales | Selaginellaceae | *Selaginella moellendorffii* | SmWOXII | GenBank |
| **Pteridophyta** |  |  |  |  |
| Dennstaedtiales | Dennstaedtiaceae | *Dennstaedtia davallioides* | MTGC-0018210-2008838 | 1KP |
| Dennstaedtiales | Dennstaedtiaceae | *Dennstaedtia davallioides* | MTGC-0054078-2041800 | 1KP |

**S1 Table.** (Continued)

| Order | Family | Species | Protein | Source |
| --- | --- | --- | --- | --- |
| Equisetales | Equisetaceae | *Equisetum diffusum* | CAPN-1064030 | 1KP |
| Equisetales | Equisetaceae | *Equisetum diffusum* | CAPN-2003425-1002577 | 1KP |
| Marattiales | Marattiaceae | *Angiopteris evecta* | NHCM-0006759-2007737 | 1KP |
| Polypodiales | Athyriaceae | *Athyrium filix-femina* | URCP-0010247-2012726 | 1KP |
| Polypodiales | Athyriaceae | *Deparia lobato-crenata* | FCHS-2061721-0024022 | 1KP |
| Polypodiales | Blechnaceae | *Blechnum spican* | AFPO-0001616-2064804 | 1KP |
| Polypodiales | Blechnaceae | *Blechnum spican* | AFPO-0108035-2071039 | 1KP |
| Polypodiales | Blechnaceae | *Blechnum spican* | AFPO-0109261-2072799 | 1KP |
| Polypodiales | Davalliaceae | *Leucostegia immersa* | WGTU-0114762-2072504 | 1KP |
| Polypodiales | Davalliaceae | *Leucostegia immersa* | WGTU-0115094-2074441 | 1KP |
| Polypodiales | Davalliaceae | *Leucostegia immersa* | WGTU-2074056-0115915 | 1KP |
| Polypodiales | Dryopteridaceae | *Cystopteris reevesiana* | RICC-0083154-2013324 | 1KP |
| Polypodiales | Dryopteridaceae | *Cystopteris utahensis* | HNDZ-2017383-0166030 | 1KP |
| Polypodiales | Dryopteridaceae | *Cystopteris utahensis* | HNDZ-2128328-0092550 | 1KP |
| Polypodiales | Dryopteridaceae | *Homalosorus pycnocarpos* | OCZL-0073099-2057736 | 1KP |
| Polypodiales | Dryopteridaceae | *Polystichum acrostichoides* | FQGQ-0002555-2007393 | 1KP |
| Polypodiales | Dryopteridaceae | *Polystichum acrostichoides* | FQGQ-2073297-0021129 | 1KP |
| Polypodiales | Lindsaeaceae | *Lindsaea linearis* | NOKI-0144212-2018564 | 1KP |
| Polypodiales | Lindsaeaceae | *Lindsaea linearis* | NOKI-0144677-2095631 | 1KP |
| Polypodiales | Nephrolepidaceae | *Nephrolepis exaltata* | NWWI-2020716-0150453-0162207-2020715 | 1KP |
| Polypodiales | Nephrolepidaceae | *Nephrolepis exaltata* | NWWI-2023299-0170781-2023298 | 1KP |
| Polypodiales | Polypodiaceae | *Polypodium amorphum* | YLJA-0105236-2014791 | 1KP |
| Polypodiales | Pteridaceae | *Ceratopteris richardii* | CrWUL | GenBank |
| Polypodiales | Pteridaceae | *Ceratopteris richardii* | CrWOX13A | GenBank |
| Polypodiales | Pteridaceae | *Ceratopteris richardii* | CrWOX13B | GenBank |
| Polypodiales | Pteridaceae | *Ceratopteris richardii* | CrWOXA | GenBank |
| Polypodiales | Pteridaceae | *Ceratopteris richardii* | CrWOXB | GenBank |

**S1 Table.** (Continued)

| Order | Family | Species | Protein | Source |
| --- | --- | --- | --- | --- |
| Polypodiales | Pteridaceae | *Cheilanthes eatonii* | GSXD-0091255-2060862 | 1KP |
| Polypodiales | Pteridaceae | *Cheilanthes eatonii* | GSXD-2013887-0006340 | 1KP |
| Polypodiales | Pteridaceae | *Cryptogramma acrostichoides* | WQML-0116973-2068365 | 1KP |
| Polypodiales | Pteridaceae | *Cryptogramma acrostichoides* | WQML-2061044-0100065 | 1KP |
| Psilotales | Psilotaceae | *Psilotum nudum* | QVMR-0021398 | 1KP |
| Pteridales | Pteridaceae | *Pityrogramma trifoliata* | UJTT-0125040-2090070 | 1KP |
| Pteridales | Pteridaceae | *Pityrogramma trifoliata* | UJTT-2086832 | 1KP |
| Salviniales | Salviniaceae | *Azolla* cristata | CVEG-2005931-2005932 | 1KP |
| Salviniales | Salviniaceae | *Azolla* cristata | CVEG-2014330-0170869 | 1KP |
| Salviniales | Salviniaceae | *Azolla filiculoides* | Azfi.s0014.g013545 | Fernbase |
| Salviniales | Salviniaceae | *Azolla filiculoides* | Azfi.s0051.g031311 | Fernbase |
| Salviniales | Salviniaceae | *Azolla filiculoides* | Azfi.s0078.g038164 | Fernbase |
| Salviniales | Salviniaceae | *Azolla filiculoides* | Azfi.s0123.g048427 | Fernbase |
| Salviniales | Salviniaceae | *Azolla filiculoides* | Azfi.s0251.g060288 | Fernbase |
| Salviniales | Salviniaceae | *Azolla filiculoides* | Azfi.s0288.g063190 | Fernbase |
| Salviniales | Salviniaceae | *Azolla filiculoides* | Azfi.s0343.g065738 | Fernbase |
| Salviniales | Salviniaceae | *Azolla filiculoides* | Azfi.s0386.g067561 | Fernbase |
| Salviniales | Salviniaceae | *Salvinia cucullata* | Sacu_v1.1_s0032.g010780 | Fernbase |
| Salviniales | Salviniaceae | *Salvinia cucullata* | Sacu_v1.1_s0036.g011598 | Fernbase |
| Salviniales | Salviniaceae | *Salvinia cucullata* | Sacu_v1.1_s0053.g14086 | Fernbase |
| Salviniales | Salviniaceae | *Salvinia cucullata* | Sacu_v1.1_s0087.g018454 | Fernbase |
| Salviniales | Salviniaceae | *Salvinia cucullata* | Sacu_v1.1_s0125.g021660 | Fernbase |
| Schizaeales | Lygodiaceae | *Lygodium japonicum* | PBUU-0104684-2076157 | 1KP |
| Ophioglossales | Ophioglossaceae | *Ophioglossum petiolatum* | WTJG-2011041 | 1KP |
| Ophioglossales | Ophioglossaceae | *Ophioglossum petiolatum* | WTJG-2015450 | 1KP |
| Ophioglossales | Ophioglossaceae | *Ophioglossum petiolatum* | WTJG-2015451 | 1KP |
| Osmundales | Osmundaceae | *Osmunda* sp. | UOMY-0008032-2004816 | 1KP |
| Osmundales | Osmundaceae | *Osmunda* sp. | UOMY-0130637-2077588 | 1KP |
| Osmundales | Osmundaceae | *Osmunda* sp. | UOMY-2079629-0130527 | 1KP |

**S1 Table.** (Continued)

| Order | Family | Species | Protein | Source |
| --- | --- | --- | --- | --- |
| Salviniales | Marsileaceae | *Pilularia globulifera* | KIIX-0137778-2085500 | 1KP |
| Salviniales | Marsileaceae | *Pilularia globulifera* | KIIX-2088043-0006942 | 1KP |
| **Cycadophyta** |  |  |  |  |
| Cycadales | Stangeriaceae | *Stangeria eriopus* | KAWQ-0001926-2040983 | 1KP |
| Cycadales | Stangeriaceae | *Stangeria eriopus* | KAWQ-2050073-0066567 | 1KP |
| Cycadales | Zamiaceae | *Dioon edule* | WLIC-0001953-2000271 | 1KP |
| Cycadales | Zamiaceae | *Encephalartos barteri* | GNQG-0002109-2005713 | 1KP |
| Cycadales | Zamiaceae | *Encephalartos barteri* | GNQG-0026245-2083844 | 1KP |
| Cycadales | Zamiaceae | *Encephalartos barteri* | GNQG-0034709-2080516 | 1KP |
| **Ginkgophyta** |  |  |  |  |
| Ginkgoales | Ginkgoaceae | *Ginkgo biloba* | GbWUS | GenBank |
| Ginkgoales | Ginkgoaceae | *Ginkgo biloba* | GbWOX2 | GenBank |
| Ginkgoales | Ginkgoaceae | *Ginkgo biloba* | GbWOX3A | GenBank |
| Ginkgoales | Ginkgoaceae | *Ginkgo biloba* | GbWOX3B | GenBank |
| Ginkgoales | Ginkgoaceae | *Ginkgo biloba* | GbWOX4 | GenBank |
| Ginkgoales | Ginkgoaceae | *Ginkgo biloba* | GbWOX9 | GenBank |
| Ginkgoales | Ginkgoaceae | *Ginkgo biloba* | GbWOX13 | GenBank |
| **Gnetophyta** |  |  |  |  |
| Gnetales | Gnetaceae | *Gnetum gnemon* | GgWUS | GenBank |
| Gnetales | Gnetaceae | *Gnetum gnemon* | GgWOX2A | GenBank |
| Gnetales | Gnetaceae | *Gnetum gnemon* | GgWOX2B | GenBank |
| Gnetales | Gnetaceae | *Gnetum gnemon* | GgWOX3 | GenBank |
| Gnetales | Gnetaceae | *Gnetum gnemon* | GgWOX4 | GenBank |
| Gnetales | Gnetaceae | *Gnetum gnemon* | GgWOX9 | GenBank |
| Gnetales | Gnetaceae | *Gnetum gnemon* | GgWOX13 | GenBank |
| Gnetales | Gnetaceae | *Gnetum gnemon* | GgWOXX | GenBank |
| Gnetales | Gnetaceae | *Gnetum gnemon* | GgWOXY | GenBank |
| Welwitschiales | Welwitschiaceae | *Welwitschia mirabilis* | TOXE-0087621-2059680 | 1KP |
| Welwitschiales | Welwitschiaceae | *Welwitschia mirabilis* | TOXE-2002028-0088417 | 1KP |
| **Pinophyta** |  |  |  |  |
| Pinales | Araucariaceae | *Araucaria rulei* | XTZO-0011053 | 1KP |
| Pinales | Araucariaceae | *Araucaria rulei* | XTZO-0091187 | 1KP |
| Pinales | Araucariaceae | *Araucaria rulei* | XTZO-0099042-2064458 | 1KP |
| Pinales | Araucariaceae | *Araucaria rulei* | XTZO-2064378 | 1KP |

**S1 Table.** (Continued)

| Order | Family | Species | Protein | Source |
| --- | --- | --- | --- | --- |
| Pinales | Cupressaceae | *Cunninghamia lanceolata* | ESYX-2015559 | 1KP |
| Pinales | Pinaceae | *Pinus sylvestris* | PsWOX2 | GenBank |
| Pinales | Pinaceae | *Pinus sylvestris* | PsWOX4 | GenBank |
| Pinales | Pinaceae | *Pinus sylvestris* | PsWOX5 | GenBank |
| Pinales | Podocarpaceae | *Saxegothaea conspicua* | QCGM-2074411-0104552 | 1KP |
| Pinales | Taxaceae | *Torreya taxifolia* | EFMS-0106008-2016046 | 1KP |
| Pinales | Taxaceae | *Torreya taxifolia* | EFMS-0108793-2077185 | 1KP |
| Pinales | Taxaceae | *Torreya taxifolia* | EFMS-2010250-0011904 | 1KP |
| **Magnoliophyta** |  |  |  |  |
| Alismatales | Posidoniaceae | *Posidonia australis* | BYQM-0012187-2058209 | 1KP |
| Alismatales | Posidoniaceae | *Posidonia australis* | BYQM-0043609-2007149-2007150-0088522 | 1KP |
| Alismatales | Posidoniaceae | *Posidonia australis* | BYQM-2061828-0055683 | 1KP |
| Alismatales | Zosteraceae | *Zostera marina* | Zosma103g00490.1 | Phytozome |
| Alismatales | Zosteraceae | *Zostera marina* | Zosma107g00040.1 | Phytozome |
| Alismatales | Zosteraceae | *Zostera marina* | Zosma114g00510.1 | Phytozome |
| Alismatales | Zosteraceae | *Zostera marina* | Zosma128g00240.1 | Phytozome |
| Alismatales | Zosteraceae | *Zostera marina* | Zosma147g00110.1 | Phytozome |
| Alismatales | Zosteraceae | *Zostera marina* | Zosma31g00850.1 | Phytozome |
| Alismatales | Zosteraceae | *Zostera marina* | Zosma34g00290.1 | Phytozome |
| Alismatales | Zosteraceae | *Zostera marina* | Zosma376g00140.1 | Phytozome |
| Alismatales | Zosteraceae | *Zostera marina* | Zosma44g00200.1 | Phytozome |
| Alismatales | Zosteraceae | *Zostera marina* | Zosma52g00180.1 | Phytozome |
| Alismatales | Zosteraceae | *Zostera marina* | Zosma537g00050.1 | Phytozome |
| Alismatales | Zosteraceae | *Zostera marina* | Zosma53g00970.1 | Phytozome |
| Alismatales | Zosteraceae | *Zostera marina* | Zosma6g01650.1 | Phytozome |
| Amborellales | Amborellaceae | *Amborella trichopoda* | evm_27.model.AmTr_v1.0_scaffold00010.77 | Amborella Genome Database |
| Amborellales | Amborellaceae | *Amborella trichopoda* | evm_27.model.AmTr_v1.0_scaffold00012.85 | Amborella Genome Database |
| Amborellales | Amborellaceae | *Amborella trichopoda* | evm_27.model.AmTr_v1.0_scaffold00021.149 | Amborella Genome Database |
| Amborellales | Amborellaceae | *Amborella trichopoda* | evm_27.model.AmTr_v1.0_scaffold00023.118 | Amborella Genome Database |

**S1 Table.** (Continued)

| Order | Family | Species | Protein | Source |
| --- | --- | --- | --- | --- |
| Amborellales | Amborellaceae | *Amborella trichopoda* | evm_27.model.AmTr_v1.0_scaffold00044.164 | Amborella Genome Database |
| Amborellales | Amborellaceae | *Amborella trichopoda* | evm_27.model.AmTr_v1.0_scaffold00051.5 | Amborella Genome Database |
| Amborellales | Amborellaceae | *Amborella trichopoda* | evm_27.model.AmTr_v1.0-scaffold00119.79 | Amborella Genome Database |
| Amborellales | Amborellaceae | *Amborella trichopoda* | evm_27.model.AmTr_v1.0_scaffold00405.1 | Amborella Genome Database |
| Apiales | Apiaceae | *Angelica archangelica* | TQKZ-0018646-2014914 | 1KP |
| Apiales | Apiaceae | *Angelica archangelica* | TQKZ-0093516-2059493 | 1KP |
| Apiales | Apiaceae | *Centella asiatica* | WEQK-2011756 | 1KP |
| Apiales | Apiaceae | *Centella asiatica* | WEQK-2059751 | 1KP |
| Apiales | Apiaceae | *Centella asiatica* | WEQK-2064831 | 1KP |
| Apiales | Apiaceae | *Heracleum lanatum* | CWYJ-0021459-2010758 | 1KP |
| Apiales | Apiaceae | *Heracleum lanatum* | CWYJ-0022221-2061432 | 1KP |
| Apiales | Apiaceae | *Heracleum lanatum* | CWYJ-0103816-2067087 | 1KP |
| Apiales | Apiaceae | *Heracleum lanatum* | CWYJ-2002939-0102612 | 1KP |
| Apiales | Araliaceae | *Hydrocotyle umbellata* | OINM-0190311-2145431 | 1KP |
| Apiales | Araliaceae | *Hydrocotyle umbellata* | OINM-2019034 | 1KP |
| Apiales | Griseliniaceae | *Griselinia littoralis* | MVSE-0012250-2005227-0169539-0038371-2005225-2005226 | 1KP |
| Apiales | Griseliniaceae | *Griselinia littoralis* | MVSE-0055462-2193860-2244533 | 1KP |
| Apiales | Pittosporaceae | *Pittosporum resiniferum* | SALZ-0102801 | 1KP |
| Apiales | Pittosporaceae | *Pittosporum resiniferum* | SALZ-0104014-2065895 | 1KP |
| Apiales | Pittosporaceae | *Pittosporum resiniferum* | SALZ-0105928-2010031 | 1KP |
| Apiales | Pittosporaceae | *Pittosporum resiniferum* | SALZ-2065336 | 1KP |
| Apiales | Pittosporaceae | *Pittosporum sahnianum* | NTEO-0086634-2061146 | 1KP |
| Apiales | Pittosporaceae | *Pittosporum sahnianum* | NTEO-0091153 | 1KP |
| Apiales | Pittosporaceae | *Pittosporum sahnianum* | NTEO-2010661 | 1KP |

**S1 Table.** (Continued)

| Order | Family | Species | Protein | Source |
| --- | --- | --- | --- | --- |
| Apiales | Pittosporaceae | *Pittosporum sahnianum* | NTEO-2066335-0091785 | 1KP |
| Aquifoliales | Aquifoliaceae | *Ilex vomitoria* | ASMV-0115184-2016620-0128798 | 1KP |
| Aquifoliales | Aquifoliaceae | *Ilex vomitoria* | ASMV-2020437-0135628 | 1KP |
| Aquifoliales | Aquifoliaceae | *Ilex vomitoria* | ASMV-2106652-0122481 | 1KP |
| Aquifoliales | Helwingiaceae | *Helwingia japonica* | QACK-2077166-0085175-0105245 | 1KP |
| Arecales | Arecaceae | *Phoenix dactylifer* | Phoenix-dactylifera-PDK-30s6550951L016 | CoGe |
| Arecales | Arecaceae | *Phoenix dactylifer* | Phoenix-dactylifera-PDK-30s668601L002 | CoGe |
| Arecales | Arecaceae | *Phoenix dactylifer* | Phoenix-dactylifera-PDK-30s691231L002 | CoGe |
| Arecales | Arecaceae | *Phoenix dactylifer* | Phoenix-dactylifera-PDK-30s713601L001 | CoGe |
| Arecales | Arecaceae | *Phoenix dactylifer* | Phoenix-dactylifera-PDK-30s720441L001 | CoGe |
| Arecales | Arecaceae | *Phoenix dactylifer* | Phoenix-dactylifera-PDK-30s771701L001 | CoGe |
| Arecales | Arecaceae | *Phoenix dactylifer* | Phoenix-dactylifera-PDK-30s800801L004 | CoGe |
| Arecales | Arecaceae | *Phoenix dactylifer* | Phoenix-dactylifera-PDK-30s806851L001 | CoGe |
| Arecales | Arecaceae | *Phoenix dactylifer* | Phoenix-dactylifera-PDK-30s945591L001 | CoGe |
| Arecales | Arecaceae | *Phoenix dactylifer* | Phoenix-dactylifera-PDK-30s988751L003 | CoGe |
| Asparagales | Amaryllidaceae | *Agapanthus africanus* | PRFO-0165768-2026043 | 1KP |
| Asparagales | Amaryllidaceae | *Agapanthus africanus* | PRFO-2028252-0156456 | 1KP |
| Asparagales | Amaryllidaceae | *Narcissus viridiflorus* | XEUV-0003320-2125672 | 1KP |
| Asparagales | Amaryllidaceae | *Narcissus viridiflorus* | XEUV-0040289-2126473 | 1KP |
| Asparagales | Amaryllidaceae | *Narcissus viridiflorus* | XEUV-0197476-2128941 | 1KP |
| Asparagales | Asparagaceae | *Agave tequilana* | KXSK-2035281 | 1KP |
| Asparagales | Asparagaceae | *Agave tequilana* | KXSK-2035282 | 1KP |
| Asparagales | Asparagaceae | *Asparagus densiflorus* | FGRF-0021717-2057669 | 1KP |
| Asparagales | Asparagaceae | *Asparagus densiflorus* | FGRF-2052852-0076424 | 1KP |
| Asparagales | Asparagaceae | *Nolina atopocarpa* | HOKG-2104732-0033847-2026324-0161321 | 1KP |

**S1 Table.** (Continued)

| Order | Family | Species | Protein | Source |
| --- | --- | --- | --- | --- |
| Asparagales | Asphodelaceae | *Johnsonia pubescens* | WTDE-0162387-2029351-0041859-0129060 | 1KP |
| Asparagales | Boryaceae | *Borya sphaerocephala* | EMJJ-0023663-2022832 | 1KP |
| Asparagales | Boryaceae | *Borya sphaerocephala* | EMJJ-2023309-0040550 | 1KP |
| Asterales | Asteraceae | *Erigeron canadensis* | DESP-0019468-0004974-2103972 | 1KP |
| Asterales | Asteraceae | *Erigeron canadensis* | DESP-2023225-0031570 | 1KP |
| Asterales | Campanulaceae | *Lobelia siphilitica* | IZLO-0012733-2011212 | 1KP |
| Asterales | Campanulaceae | *Lobelia siphilitica* | IZLO-0014627-2007560 | 1KP |
| Asterales | Campanulaceae | *Lobelia siphilitica* | IZLO-0019247-2061371 | 1KP |
| Asterales | Campanulaceae | *Lobelia siphilitica* | IZLO-0019417-2062176 | 1KP |
| Asterales | Campanulaceae | *Lobelia siphilitica* | IZLO-0020347-2056247 | 1KP |
| Asterales | Campanulaceae | *Lobelia siphilitica* | IZLO-0094855-2058195 | 1KP |
| Asterales | Goodeniaceae | *Scaevola sp.* | HUQC-0023556-2071489-0057680-0094343 | 1KP |
| Berberidopsidales | Aextoxicaceae | *Aextoxicon punctatum* | QUTB-0024939-2014474 | 1KP |
| Berberidopsidales | Aextoxicaceae | *Aextoxicon punctatum* | QUTB-0104304-2070670 | 1KP |
| Berberidopsidales | Aextoxicaceae | *Aextoxicon punctatum* | QUTB-0107170-2072179 | 1KP |
| Berberidopsidales | Aextoxicaceae | *Aextoxicon punctatum* | QUTB-2001029-0106585 | 1KP |
| Berberidopsidales | Aextoxicaceae | *Aextoxicon punctatum* | QUTB-2014473 | 1KP |
| Boraginales | Boraginaceae | *Heliotropium calcicola* | VJSJ-2013549-0022735-2013551-2013550-0004311 | 1KP |
| Boraginales | Boraginaceae | *Heliotropium calcicola* | VJSJ-2019550-0067143 | 1KP |
| Boraginales | Boraginaceae | *Heliotropium calcicola* | VJSJ-2019551 | 1KP |
| Boraginales | Boraginaceae | *Heliotropium calcicola* | VJSJ-2019552 | 1KP |
| Boraginales | Boraginaceae | *Phacelia campanularia* | YQIJ-0009590-2043426 | 1KP |
| Brassicales | Brassicaceae | *Arabidopsis thaliana* | AtWUS | TAIR |
| Brassicales | Brassicaceae | *Arabidopsis thaliana* | AtWOX1 | TAIR |
| Brassicales | Brassicaceae | *Arabidopsis thaliana* | AtWOX2 | TAIR |
| Brassicales | Brassicaceae | *Arabidopsis thaliana* | AtWOX3 | TAIR |

**S1 Table.** (Continued)

| Order | Family | Species | Protein | Source |
| --- | --- | --- | --- | --- |
| Brassicales | Brassicaceae | *Arabidopsis thaliana* | AtWOX4 | TAIR |
| Brassicales | Brassicaceae | *Arabidopsis thaliana* | AtWOX5 | TAIR |
| Brassicales | Brassicaceae | *Arabidopsis thaliana* | AtWOX6 | TAIR |
| Brassicales | Brassicaceae | *Arabidopsis thaliana* | AtWOX7 | TAIR |
| Brassicales | Brassicaceae | *Arabidopsis thaliana* | AtWOX8 | TAIR |
| Brassicales | Brassicaceae | *Arabidopsis thaliana* | AtWOX9 | TAIR |
| Brassicales | Brassicaceae | *Arabidopsis thaliana* | AtWOX10 | TAIR |
| Brassicales | Brassicaceae | *Arabidopsis thaliana* | AtWOX11 | TAIR |
| Brassicales | Brassicaceae | *Arabidopsis thaliana* | AtWOX12 | TAIR |
| Brassicales | Brassicaceae | *Arabidopsis thaliana* | AtWOX13 | TAIR |
| Brassicales | Brassicaceae | *Arabidopsis thaliana* | AtWOX14 | TAIR |
| Brassicales | Brassicaceae | *Sinapis alba* | VMNH-0037835-2047005-2022014-0087986 | 1KP |
| Brassicales | Brassicaceae | *Sinapis alba* | VMNH-2004470-0011379-0011378-0026103-2043466 | 1KP |
| Brassicales | Caricaceae | *Carica papaya* | evm.model.supercontig-139.11 | Phytozome |
| Brassicales | Caricaceae | *Carica papaya* | evm-model-supercontig-19.201 | Phytozome |
| Brassicales | Caricaceae | *Carica papaya* | evm.model.supercontig -190.1 | Phytozome |
| Brassicales | Caricaceae | *Carica papaya* | evm.model.supercontig -21.170 | Phytozome |
| Brassicales | Caricaceae | *Carica papaya* | evm.model.supercontig -26.173 | Phytozome |
| Brassicales | Caricaceae | *Carica papaya* | evm.model.supercontig -3.402 | Phytozome |
| Brassicales | Caricaceae | *Carica papaya* | evm.model.supercontig -6.244 | Phytozome |
| Brassicales | Caricaceae | *Carica papaya* | evm.model.supercontig -6.368 | Phytozome |
| Brassicales | Caricaceae | *Carica papaya* | evm.model.supercontig -65.159 | Phytozome |
| Brassicales | Caricaceae | *Carica papaya* | evm.model.supercontig -7.12 | Phytozome |
| Brassicales | Caricaceae | *Carica papaya* | evm.model.supercontig -73.3 | Phytozome |
| Brassicales | Moringaceae | *Moringa oleifera* | CZPV-0002050-2045438 | 1KP |

**S1 Table.** (Continued)

| Order | Family | Species | Protein | Source |
| --- | --- | --- | --- | --- |
| Brassicales | Moringaceae | *Moringa oleifera* | CZPV-0009321-2046134 | 1KP |
| Brassicales | Moringaceae | *Moringa oleifera* | CZPV-0017384-2010136 | 1KP |
| Brassicales | Moringaceae | *Moringa oleifera* | CZPV-0018524-2047105 | 1KP |
| Brassicales | Moringaceae | *Moringa oleifera* | CZPV-0058878-2048720 | 1KP |
| Brassicales | Salvadoraceae | *Salvadora sp.* | RTTY-2007455-0073173 | 1KP |
| Brassicales | Salvadoraceae | *Salvadora sp.* | RTTY-2015596-0053901-0055029-0083989 | 1KP |
| Buxales | Buxaceae | *Buxus sempervirens* | IWMW-0115973-2003795 | 1KP |
| Buxales | Buxaceae | *Buxus sempervirens* | IWMW-0125376-2087284 | 1KP |
| Canellales | Canellaceae | *Canella winterana* | DDEV-0029566-2018080 | 1KP |
| Canellales | Canellaceae | *Canella winterana* | DDEV-0034177-2115697-0151927 | 1KP |
| Canellales | Canellaceae | *Canella winterana* | DDEV-2013055-0022145-0022027 | 1KP |
| Canellales | Winteraceae | *Drimys winteri* | WKSU-0004462-2035412-2081530 | 1KP |
| Canellales | Winteraceae | *Drimys winteri* | WKSU-0027838-2126706 | 1KP |
| Canellales | Winteraceae | *Drimys winteri* | WKSU-0044287-2029375 | 1KP |
| Caryophyllales | Aizoaceae | *Delosperma echinatum* | BJKT-0010281-2066518 | 1KP |
| Caryophyllales | Aizoaceae | *Delosperma echinatum* | BJKT-2064304-0091394 | 1KP |
| Caryophyllales | Amaranthaceae | *Amaranthus cruentus* | XSSD-0020343-2012139 | 1KP |
| Caryophyllales | Amaranthaceae | *Amaranthus cruentus* | XSSD-0129333-2016403-2016401 | 1KP |
| Caryophyllales | Amaranthaceae | *Amaranthus cruentus* | XSSD-2077884-0005895 | 1KP |
| Caryophyllales | Cactaceae | *Lophophora williamsii* | CPKP-2008161 | 1KP |
| Caryophyllales | Cactaceae | *Lophophora williamsii* | CPKP-2098735 | 1KP |
| Caryophyllales | Caryophyllaceae | *Polycarpaea repens* | RXEN-2015151 | 1KP |
| Caryophyllales | Caryophyllaceae | *Polycarpaea repens* | RXEN-2015865 | 1KP |
| Caryophyllales | Caryophyllaceae | *Silene latifolia* | FZQN-0000377-2011724 | 1KP |
| Caryophyllales | Nyctaginaceae | *Boerhavia coccinea* | MBWM-0118544-2003669 | 1KP |
| Caryophyllales | Nyctaginaceae | *Boerhavia coccinea* | MBWM-0120730-2002373 | 1KP |
| Caryophyllales | Phytolaccaceae | *Hilleria latifolia* | SFKQ-0125783-2087471 | 1KP |

**S1 Table.** (Continued)

| Order | Family | Species | Protein | Source |
| --- | --- | --- | --- | --- |
| Caryophyllales | Phytolaccaceae | *Hilleria latifolia* | SFKQ-2005754 | 1KP |
| Caryophyllales | Phytolaccaceae | *Phytolacca bogotensis* | MRKX-0009173 | 1KP |
| Caryophyllales | Phytolaccaceae | *Phytolacca bogotensis* | MRKX-0027470-2008077 | 1KP |
| Caryophyllales | Phytolaccaceae | *Phytolacca bogotensis* | MRKX-0034157-2019429 | 1KP |
| Caryophyllales | Portulaceae | *Portulaca oleracea* | CZJT-2023305-0140064 | 1KP |
| Celastrales | Celastraceae | *Crossopetalum rhacoma* | IHCQ-0098379-2020522-0110891 | 1KP |
| Chloranthales | Chloranthaceae | *Ascarina rubricaulis* | WZFE-0303380-2189389 | 1KP |
| Chloranthales | Chloranthaceae | *Ascarina rubricaulis* | WZFE-2000595-0115034-0243109 | 1KP |
| Chloranthales | Chloranthaceae | *Sarcandra glabra* | OSHQ-0009284-2047644 | 1KP |
| Cornales | Cornaceae | *Cornus florida* | BFJL-0027693-2193893 | 1KP |
| Cornales | Cornaceae | *Cornus florida* | BFJL-0033627-2009922 | 1KP |
| Cornales | Cornaceae | *Cornus florida* | BFJL-0297929-2193671 | 1KP |
| Cornales | Cornaceae | *Cornus florida* | BFJL-0298861-2025545 | 1KP |
| Cornales | Loasaceae | *Caiophora chuquitensis* | VTLJ-2013756-0029237 | 1KP |
| Cornales | Loasaceae | *Caiophora chuquitensis* | VTLJ-2068561-0032830 | 1KP |
| Crossosomatales | Staphyleaceae | *Staphylea trifolia* | PTLU-0021185-2007498 | 1KP |
| Crossosomatales | Staphyleaceae | *Staphylea trifolia* | PTLU-0022857 | 1KP |
| Crossosomatales | Staphyleaceae | *Staphylea trifolia* | PTLU-0131320-2098950 | 1KP |
| Crossosomatales | Staphyleaceae | *Staphylea trifolia* | PTLU-0131802-2100998-0121284 | 1KP |
| Crossosomatales | Staphyleaceae | *Staphylea trifolia* | PTLU-2013721-0082914 | 1KP |
| Crossosomatales | Staphyleaceae | *Staphylea trifolia* | PTLU-2013723-0017590-2013720 | 1KP |
| Crossosomatales | Staphyleaceae | *Staphylea trifolia* | PTLU-2017617-0122028 | 1KP |
| Crossosomatales | Staphyleaceae | *Staphylea trifolia* | PTLU-2094150 | 1KP |
| Cucurbitales | Cucurbitaceae | *Cucumis sativus* | Cucsa.043520.1 | Phytozome |
| Cucurbitales | Cucurbitaceae | *Cucumis sativus* | Cucsa.046640.1 | Phytozome |
| Cucurbitales | Cucurbitaceae | *Cucumis sativus* | Cucsa.111520.1 | Phytozome |
| Cucurbitales | Cucurbitaceae | *Cucumis sativus* | Cucsa.121900.1 | Phytozome |
| Cucurbitales | Cucurbitaceae | *Cucumis sativus* | Cucsa.136500.1 | Phytozome |
| Cucurbitales | Cucurbitaceae | *Cucumis sativus* | Cucsa.213810.1 | Phytozome |
| Cucurbitales | Cucurbitaceae | *Cucumis sativus* | Cucsa.272210.1 | Phytozome |
| Cucurbitales | Cucurbitaceae | *Cucumis sativus* | Cucsa.323040.1 | Phytozome |
| Cucurbitales | Cucurbitaceae | *Cucumis sativus* | Cucsa.336980.1 | Phytozome |
| Cucurbitales | Cucurbitaceae | *Cucumis sativus* | Cucsa.379650.1 | Phytozome |
| Cucurbitales | Cucurbitaceae | *Cucumis sativus* | Cucsa.395970.1 | Phytozome |

**S1 Table.** (Continued)

| Order | Family | Species | Protein | Source |
| --- | --- | --- | --- | --- |
| Cucurbitales | Apodanthaceae | *Pilostyles thurberi* | NJJO-0004574-2028832 | 1KP |
| Cucurbitales | Apodanthaceae | *Pilostyles thurberi* | NJJO-0010717-2006475-2006476 | 1KP |
| Cucurbitales | Apodanthaceae | *Pilostyles thurberi* | NJJO-0011497-2029905 | 1KP |
| Cucurbitales | Apodanthaceae | *Pilostyles thurberi* | NJJO-0043014-2007893 | 1KP |
| Cucurbitales | Apodanthaceae | *Pilostyles thurberi* | NJJO-0043221-2008829 | 1KP |
| Dilleniales | Dilleniaceae | *Dillenia indica* | EHNF-0000263-0005424-2053778-0005423-2009284 | 1KP |
| Dilleniales | Dilleniaceae | *Dillenia indica* | EHNF-0027644-2086410 | 1KP |
| Dilleniales | Dilleniaceae | *Dillenia indica* | EHNF-2016980 | 1KP |
| Dilleniales | Dilleniaceae | *Dillenia indica* | EHNF-2020763-0000857-0083750-2020764-2016981 | 1KP |
| Dioscoreales | Dioscoreaceae | *Dioscorea villosa* | OCWZ-2049453 | 1KP |
| Dipsacales | Adoxaceae | *Sambucus canadensis* | QRNU-0019518-2017708 | 1KP |
| Dipsacales | Adoxaceae | *Sambucus canadensis* | QRNU-0174730-2019788-0152325 | 1KP |
| Dipsacales | Adoxaceae | *Sambucus canadensis* | QRNU-0221021-2017707 | 1KP |
| Dipsacales | Adoxaceae | *Sambucus canadensis* | QRNU-0253378-2019540 | 1KP |
| Dipsacales | Adoxaceae | *Sambucus canadensis* | QRNU-0254376-2019227 | 1KP |
| Dipsacales | Adoxaceae | *Sambucus canadensis* | QRNU-0257809-2170087 | 1KP |
| Dipsacales | Adoxaceae | *Sambucus canadensis* | QRNU-2016093-0229174 | 1KP |
| Dipsacales | Adoxaceae | *Sambucus canadensis* | QRNU-2019465 | 1KP |
| Dipsacales | Adoxaceae | *Sambucus canadensis* | SNBI-0003071-2088974 | 1KP |
| Dipsacales | Caprifoliaceae | *Lonicera japonica* | GSZA-0019493-2068547 | 1KP |
| Dipsacales | Caprifoliaceae | *Lonicera japonica* | GSZA-0019683-2006765 | 1KP |
| Dipsacales | Caprifoliaceae | *Lonicera japonica* | GSZA-0083428-2006763-0094258 | 1KP |
| Dipsacales | Caprifoliaceae | *Lonicera japonica* | GSZA-2006764 | 1KP |
| Dipsacales | Caprifoliaceae | *Symphoricarpos* sp. | CAQZ-0034283-2016852 | 1KP |
| Dipsacales | Caprifoliaceae | *Symphoricarpos* sp. | CAQZ-0132766 | 1KP |
| Dipsacales | Caprifoliaceae | *Symphoricarpos* sp. | CAQZ-0136131-2107537 | 1KP |
| Dipsacales | Caprifoliaceae | *Symphoricarpos* sp. | CAQZ-0139186 | 1KP |

**S1 Table.** (Continued)

| Order | Family | Species | Protein | Source |
| --- | --- | --- | --- | --- |
| Dipsacales | Caprifoliaceae | *Symphoricarpos* sp. | CAQZ-2012851 | 1KP |
| Dipsacales | Caprifoliaceae | *Symphoricarpos* sp. | CAQZ-2012852 | 1KP |
| Dipsacales | Dipsacaceae | *Dipsacus asper* | JTRM-0021076-2019408 | 1KP |
| Dipsacales | Dipsacaceae | *Dipsacus asper* | JTRM-0021569-2016895-2016894 | 1KP |
| Dipsacales | Dipsacaceae | *Dipsacus asper* | JTRM-0025475-2090034 | 1KP |
| Dipsacales | Dipsacaceae | *Dipsacus asper* | JTRM-0118826-2016896 | 1KP |
| Dipsacales | Dipsacaceae | *Dipsacus asper* | JTRM-0124296-2089762 | 1KP |
| Dipsacales | Dipsacaceae | *Dipsacus asper* | JTRM-2090964-0130239 | 1KP |
| Ericales | Actinidiaceae | *Actinidia chinensis* | QAUE-0035393-2028494 | 1KP |
| Ericales | Actinidiaceae | *Actinidia chinensis* | QAUE-0036708-0150926-2053976-2007173 | 1KP |
| Ericales | Actinidiaceae | *Actinidia chinensis* | QAUE-0184245-2031162 | 1KP |
| Ericales | Balsaminaceae | *Impatiens balsamifera* | JEXA-2001766 | 1KP |
| Ericales | Balsaminaceae | *Impatiens balsamifera* | JEXA-2062504 | 1KP |
| Ericales | Balsaminaceae | *Impatiens balsamifera* | JEXA-2062768 | 1KP |
| Ericales | Balsaminaceae | *Impatiens balsamifera* | JEXA-2064682 | 1KP |
| Ericales | Cyrillaceae | *Cyrilla racemiflora* | YZGX-0014734 | 1KP |
| Ericales | Cyrillaceae | *Cyrilla racemiflora* | YZGX-0022463 | 1KP |
| Ericales | Cyrillaceae | *Cyrilla racemiflora* | YZGX-0024740 | 1KP |
| Ericales | Cyrillaceae | *Cyrilla racemiflora* | YZGX-0026432 | 1KP |
| Ericales | Cyrillaceae | *Cyrilla racemiflora* | YZGX-0041386 | 1KP |
| Ericales | Cyrillaceae | *Cyrilla racemiflora* | YZGX-0163162 | 1KP |
| Ericales | Cyrillaceae | *Cyrilla racemiflora* | YZGX-0163708 | 1KP |
| Ericales | Cyrillaceae | *Cyrilla racemiflora* | YZGX-2001256-0111098-2001255-0111082 | 1KP |
| Ericales | Ericaceae | *Cavendishia cuatrecasasii* | AVJK-0041080-2025898 | 1KP |
| Ericales | Ericaceae | *Cavendishia cuatrecasasii* | AVJK-2019647-0107293-0178011 | 1KP |

**S1 Table.** (Continued)

| Order | Family | Species | Protein | Source |
| --- | --- | --- | --- | --- |
| Ericales | Ericaceae | *Cavendishia cuatrecasasii* | AVJK-2124286-0159189 | 1KP |
| Ericales | Ericaceae | *Cavendishia cuatrecasasii* | AVJK-2128395-0022214-0080938 | 1KP |
| Ericales | Fouquieriaceae | *Fouquieria macdougalii* | YSRZ-0129273-2085063 | 1KP |
| Ericales | Fouquieriaceae | *Fouquieria macdougalii* | YSRZ-0135290-2089449 | 1KP |
| Ericales | Marcgraviaceae | *Souroubea exauriculata* | BNTL-0002120-2020621-2020620-2020619-2187-2078748 | 1KP |
| Ericales | Polemoniaceae | *Phlox* sp. | FNEN-0097664-2071352 | 1KP |
| Ericales | Polemoniaceae | *Phlox* sp. | FNEN-0100184-2005515-2005513 | 1KP |
| Ericales | Sapotaceae | *Synsepalum dulcificum* | WRPP-0018654-2022439 | 1KP |
| Ericales | Sapotaceae | *Synsepalum dulcificum* | WRPP-0173907 | 1KP |
| Ericales | Sapotaceae | *Synsepalum dulcificum* | WRPP-0193665 | 1KP |
| Ericales | Sapotaceae | *Synsepalum dulcificum* | WRPP-0196279-2130384 | 1KP |
| Ericales | Sapotaceae | *Synsepalum dulcificum* | WRPP-2010874 | 1KP |
| Ericales | Sapotaceae | *Synsepalum dulcificum* | WRPP-2022723 | 1KP |
| Ericales | Sapotaceae | *Synsepalum dulcificum* | WRPP-2114300-0164999 | 1KP |
| Ericales | Sapotaceae | *Synsepalum dulcificum* | WRPP-2121969 | 1KP |
| Ericales | Styraceae | *Sinojackia xylocarpa* | AXBO-0094732-2103721 | 1KP |
| Ericales | Styraceae | *Sinojackia xylocarpa* | AXBO-0102091-2101625 | 1KP |
| Ericales | Styraceae | *Sinojackia xylocarpa* | AXBO-2016612-0045981 | 1KP |
| Escalloniales | Escalloniaceae | *Escallonia rubra* | CLMX-2008796 | 1KP |
| Escalloniales | Escalloniaceae | *Escallonia rubra* | CLMX-2042310 | 1KP |
| Escalloniales | Escalloniaceae | *Escallonia rubra* | CLMX-2044326 | 1KP |
| Fabales | Fabaceae | *Glycine max* | Glyma.01G166800.1 | Phytozome |
| Fabales | Fabaceae | *Glycine max* | Glyma.02G093900.1 | Phytozome |
| Fabales | Fabaceae | *Glycine max* | Glyma.02G254800.1 | Phytozome |
| Fabales | Fabaceae | *Glycine max* | Glyma.03G007600.1 | Phytozome |
| Fabales | Fabaceae | *Glycine max* | Glyma.04G016700.1 | Phytozome |
| Fabales | Fabaceae | *Glycine max* | Glyma.04G040900.1 | Phytozome |
| Fabales | Fabaceae | *Glycine max* | Glyma.05G245900.1 | Phytozome |
| Fabales | Fabaceae | *Glycine max* | Glyma.06G016800.1 | Phytozome |

**S1 Table.** (Continued)

| Order | Family | Species | Protein | Source |
| --- | --- | --- | --- | --- |
| Fabales | Fabaceae | *Glycine max* | Glyma.06G041800.1 | Phytozome |
| Fabales | Fabaceae | *Glycine max* | Glyma.07G101000.1 | Phytozome |
| Fabales | Fabaceae | *Glycine max* | Glyma.07G131000.1 | Phytozome |
| Fabales | Fabaceae | *Glycine max* | Glyma.07G203000.1 | Phytozome |
| Fabales | Fabaceae | *Glycine max* | Glyma.07G219400.1 | Phytozome |
| Fabales | Fabaceae | *Glycine max* | Glyma.08G053700.1 | Phytozome |
| Fabales | Fabaceae | *Glycine max* | Glyma.09G177800.1 | Phytozome |
| Fabales | Fabaceae | *Glycine max* | Glyma.10G069700.1 | Phytozome |
| Fabales | Fabaceae | *Glycine max* | Glyma.10G289900.1 | Phytozome |
| Fabales | Fabaceae | *Glycine max* | Glyma.11G076500.1 | Phytozome |
| Fabales | Fabaceae | *Glycine max* | Glyma.11G140400.1 | Phytozome |
| Fabales | Fabaceae | *Glycine max* | Glyma.11G210800.1 | Phytozome |
| Fabales | Fabaceae | *Glycine max* | Glyma.11G227800.1 | Phytozome |
| Fabales | Fabaceae | *Glycine max* | Glyma.12G063900.1 | Phytozome |
| Fabales | Fabaceae | *Glycine max* | Glyma.13G172700.1 | Phytozome |
| Fabales | Fabaceae | *Glycine max* | Glyma.13G334900.1 | Phytozome |
| Fabales | Fabaceae | *Glycine max* | Glyma.14G084600.1 | Phytozome |
| Fabales | Fabaceae | *Glycine max* | Glyma.15G039600.1 | Phytozome |
| Fabales | Fabaceae | *Glycine max* | Glyma.17G240300.1 | Phytozome |
| Fabales | Fabaceae | *Glycine max* | Glyma.18G029700.1 | Phytozome |
| Fabales | Fabaceae | *Glycine max* | Glyma.18G179300.1 | Phytozome |
| Fabales | Fabaceae | *Glycine max* | Glyma.18G288000.1 | Phytozome |
| Fabales | Fabaceae | *Glycine max* | Glyma.19G118400.1 | Phytozome |
| Fabales | Fabaceae | *Glycine max* | Glyma.20G017600.1 | Phytozome |
| Fabales | Fabaceae | *Glycine max* | Glyma.20G099400.1 | Phytozome |
| Fabales | Fabaceae | *Medicago truncatula* | Medtr1g019130.1 | Phytozome |
| Fabales | Fabaceae | *Medicago truncatula* | Medtr1g115315.1 | Phytozome |
| Fabales | Fabaceae | *Medicago truncatula* | Medtr2g015000.1 | Phytozome |
| Fabales | Fabaceae | *Medicago truncatula* | Medtr3g088485.1 | Phytozome |
| Fabales | Fabaceae | *Medicago truncatula* | Medtr3g115620.1 | Phytozome |
| Fabales | Fabaceae | *Medicago truncatula* | Medtr4g063735.1 | Phytozome |
| Fabales | Fabaceae | *Medicago truncatula* | Medtr4g084550.1 | Phytozome |
| Fabales | Fabaceae | *Medicago truncatula* | Medtr4g088070.1 | Phytozome |
| Fabales | Fabaceae | *Medicago truncatula* | Medtr4g088080.1 | Phytozome |

**S1 Table.** (Continued)

| Order | Family | Species | Protein | Source |
| --- | --- | --- | --- | --- |
| Fabales | Fabaceae | *Medicago truncatula* | Medtr5g021930.1 | Phytozome |
| Fabales | Fabaceae | *Medicago truncatula* | Medtr5g081990.1 | Phytozome |
| Fabales | Fabaceae | *Medicago truncatula* | Medtr6g027390.1 | Phytozome |
| Fabales | Fabaceae | *Medicago truncatula* | Medtr7g023810.1 | Phytozome |
| Fabales | Fabaceae | *Medicago truncatula* | Medtr7g060630.1 | Phytozome |
| Fabales | Fabaceae | *Medicago truncatula* | Medtr7g086940.1 | Phytozome |
| Fabales | Fabaceae | *Medicago truncatula* | Medtr8g095580.1 | Phytozome |
| Fabales | Fabaceae | *Medicago truncatula* | Medtr8g107210.1 | Phytozome |
| Fagales | Betulaceae | *Alnus serrulata* | LWDA-0016171-2000709 | 1KP |
| Fagales | Betulaceae | *Alnus serrulata* | LWDA-0114483-2080196 | 1KP |
| Fagales | Betulaceae | *Alnus serrulata* | LWDA-2084101-0105294 | 1KP |
| Fagales | Fagaceae | *Castanea pumila* | UZWG-0006552-2093173-0093007 | 1KP |
| Fagales | Fagaceae | *Castanea pumila* | UZWG-0145343 | 1KP |
| Fagales | Fagaceae | *Castanea pumila* | UZWG-0145730-2087140 | 1KP |
| Fagales | Fagaceae | *Castanea pumila* | UZWG-2011881-0144176 | 1KP |
| Fagales | Fagaceae | *Castanea pumila* | UZWG-2013971 | 1KP |
| Fagales | Juglandaceae | *Juglans nigra* | DXQW-0006692-2006221 | 1KP |
| Fagales | Juglandaceae | *Juglans nigra* | DXQW-0013709-2050903 | 1KP |
| Fagales | Juglandaceae | *Juglans nigra* | DXQW-0020255-2054069 | 1KP |
| Fagales | Juglandaceae | *Juglans nigra* | DXQW-0028353-2006334-0055582 | 1KP |
| Fagales | Juglandaceae | *Juglans nigra* | DXQW-0095933 | 1KP |
| Fagales | Juglandaceae | *Juglans nigra* | DXQW-2013392-0105364 | 1KP |
| Fagales | Juglandaceae | *Juglans nigra* | DXQW-2056235-0075946-0009254 | 1KP |
| Fagales | Myricaceae | *Myrica cerifera* | INSP-0005681-2009214 | 1KP |
| Fagales | Myricaceae | *Myrica cerifera* | INSP-0053537-2050781 | 1KP |
| Fagales | Nothofagaceae | *Nothofagus obliqua* | TJLC-0015025-2005451 | 1KP |
| Fagales | Nothofagaceae | *Nothofagus obliqua* | TJLC-0017682-2008925 | 1KP |

**S1 Table.** (Continued)

| Order | Family | Species | Protein | Source |
| --- | --- | --- | --- | --- |
| Fagales | Nothofagaceae | *Nothofagus obliqua* | TJLC-0018561-2009815 | 1KP |
| Fagales | Nothofagaceae | *Nothofagus obliqua* | TJLC-0080383-2014061 | 1KP |
| Garryales | Garryaceae | *Aucuba japonica* | GIPR-0014470 | 1KP |
| Garryales | Garryaceae | *Aucuba japonica* | GIPR-0073112-2002250 | 1KP |
| Garryales | Garryaceae | *Aucuba japonica* | GIPR-2002249 | 1KP |
| Gentianales | Apocynaceae | *Apocynum androsaemifolium* | JCLQ-2007614-0069530 | 1KP |
| Gentianales | Apocynaceae | *Apocynum androsaemifolium* | JCLQ-2042718-0053049-0066678 | 1KP |
| Gentianales | Apocynaceae | *Rauvolfia tetraphylla* | QEHE-0138438-2091441 | 1KP |
| Gentianales | Gentianaceae | *Exacum affine* | KPUM-2014490 | 1KP |
| Gentianales | Gentianaceae | *Exacum affine* | KPUM-2099697 | 1KP |
| Gentianales | Gentianaceae | *Gentiana acaulis* | ECTD-2016009 | 1KP |
| Gentianales | Gentianaceae | *Gentiana acaulis* | ECTD-2017435 | 1KP |
| Gentianales | Gentianaceae | *Gentiana acaulis* | ECTD-2017436 | 1KP |
| Gentianales | Gentianaceae | *Gentiana acaulis* | ECTD-2109856 | 1KP |
| Gentianales | Rubiaceae | *Galium boreale* | WQRD-2013906 | 1KP |
| Gentianales | Rubiaceae | *Galium boreale* | WQRD-2057771 | 1KP |
| Geraniales | Geraniaceae | *Geranium carolinianum* | VKGP-0009380-0023921-2030063 | 1KP |
| Geraniales | Geraniaceae | *Geranium carolinianum* | VKGP-0037262-2033984 | 1KP |
| Geraniales | Geraniaceae | *Geranium carolinianum* | VKGP-0150387-2020066 | 1KP |
| Geraniales | Geraniaceae | *Geranium carolinianum* | VKGP-0169974-2106401 | 1KP |
| Geraniales | Geraniaceae | *Geranium carolinianum* | VKGP-0173806-2087025 | 1KP |
| Geraniales | Geraniaceae | *Geranium carolinianum* | VKGP-2008083-0168075-2008082 | 1KP |
| Geraniales | Geraniaceae | *Geranium carolinianum* | VKGP-2020065 | 1KP |
| Geraniales | Geraniaceae | *Geranium carolinianum* | VKGP-2020067 | 1KP |
| Lamiales | Gesneriaceae | *Saintpaulia ionantha* | RWKR-0028203-2014369 | 1KP |
| Lamiales | Gesneriaceae | *Saintpaulia ionantha* | RWKR-0035667-2118306 | 1KP |
| Lamiales | Gesneriaceae | *Saintpaulia ionantha* | RWKR-2014370 | 1KP |
| Gunnerales | Gunneraceae | *Gunnera manicata* | gmani-08989 | Chiu and Elhai |
| Gunnerales | Gunneraceae | *Gunnera manicata* | gmani-11478 | Chiu and Elhai |

**S1 Table.** (Continued)

| Order | Family | Species | Protein | Source |
| --- | --- | --- | --- | --- |
| Gunnerales | Gunneraceae | *Gunnera manicata* | gmani-16058 | Chiu and Elhai |
| Gunnerales | Gunneraceae | *Gunnera manicata* | XMQO-0053084-2133385 | 1KP |
| Gunnerales | Gunneraceae | *Gunnera manicata* | XMQO-0163298-2135075 | 1KP |
| Huerteales | Tapisciaceae | *Tapiscia sinensis* | WWKL-0103271-2012599-0111732 | 1KP |
| Huerteales | Tapisciaceae | *Tapiscia sinensis* | WWKL-0117394-2007709 | 1KP |
| Lamiales | Acanthaceae | *Strobilanthes dyeriana* | WEAC-0008734-2077116 | 1KP |
| Lamiales | Acanthaceae | *Strobilanthes dyeriana* | WEAC-0012843-2014598 | 1KP |
| Lamiales | Acanthaceae | *Strobilanthes dyeriana* | WEAC-0127163-2077849 | 1KP |
| Lamiales | Acanthaceae | *Strobilanthes dyeriana* | WEAC-2013094 | 1KP |
| Lamiales | Bignoniaceae | *Kigelia africana* | SVQC-0020092-2006535 | 1KP |
| Lamiales | Bignoniaceae | *Kigelia africana* | SVQC-0023142-2059604 | 1KP |
| Lamiales | Bignoniaceae | *Kigelia africana* | SVQC-0077814 | 1KP |
| Lamiales | Bignoniaceae | *Kigelia africana* | SVQC-0079131-2059607 | 1KP |
| Lamiales | Bignoniaceae | *Kigelia africana* | SVQC-0080047-2057574 | 1KP |
| Lamiales | Bignoniaceae | *Kigelia africana* | SVQC-2006536 | 1KP |
| Lamiales | Buddlejaceae | Buddleja sp. | GRFT-0025288-2070119 | 1KP |
| Lamiales | Buddlejaceae | *Buddleja* sp. | GRFT-0025994-2071524 | 1KP |
| Lamiales | Buddlejaceae | *Buddleja* sp. | GRFT-0027099-2014884 | 1KP |
| Lamiales | Byblidaceae | *Byblis gigantea* | GDZS-0020835-2010410 | 1KP |
| Lamiales | Byblidaceae | *Byblis gigantea* | GDZS-0032131-2097591 | 1KP |
| Lamiales | Byblidaceae | *Byblis gigantea* | GDZS-0035594-2030323 | 1KP |
| Lamiales | Byblidaceae | *Byblis gigantea* | GDZS-0138163-2109442 | 1KP |
| Lamiales | Byblidaceae | *Byblis gigantea* | GDZS-0165039-2111996 | 1KP |
| Lamiales | Byblidaceae | *Byblis gigantea* | GDZS-2022733-0147230 | 1KP |
| Lamiales | Byblidaceae | *Byblis gigantea* | GDZS-2025274-0133865-2025275-0082325-0165992 | 1KP |
| Lamiales | Calceolariaceae | *Calceolaria pinifolia* | DCCI-0009456-2012961 | 1KP |

**S1 Table.** (Continued)

| Order | Family | Species | Protein | Source |
| --- | --- | --- | --- | --- |
| Lamiales | Calceolariaceae | *Calceolaria pinifolia* | DCCI-0112071 | 1KP |
| Lamiales | Calceolariaceae | *Calceolaria pinifolia* | DCCI-0114330-2076974 | 1KP |
| Lamiales | Calceolariaceae | *Calceolaria pinifolia* | DCCI-2014049 | 1KP |
| Lamiales | Calceolariaceae | *Calceolaria pinifolia* | DCCI-2019284-0054843-0112425 | 1KP |
| Lamiales | Gesneriaceae | *Sinningia tuberosa* | DTNC-0010704-2007389 | 1KP |
| Lamiales | Gesneriaceae | *Sinningia tuberosa* | DTNC-0020179-2054257 | 1KP |
| Lamiales | Gesneriaceae | *Sinningia tuberosa* | DTNC-0061077-2050392 | 1KP |
| Lamiales | Lamiaceae | *Scutellaria montana* | ATYL-0171153-2120474 | 1KP |
| Lamiales | Lentibulariaceae | *Utricularia* sp. | HRUR-2028885-2028886-0201508 | 1KP |
| Lamiales | Lentibulariaceae | *Utricularia* sp. | HRUR-2136239-0016210-0091524 | 1KP |
| Lamiales | Oleaceae | *Forestiera segregata* | UEEN-0210688-2030697-0200055 | 1KP |
| Lamiales | Oleaceae | *Ligustrum sinense* | MZLD-0017239-2024701 | 1KP |
| Lamiales | Oleaceae | *Ligustrum sinense* | MZLD-0039569-2027538 | 1KP |
| Lamiales | Oleaceae | *Ligustrum sinense* | MZLD-0172876-2118405 | 1KP |
| Lamiales | Oleaceae | *Ligustrum sinense* | MZLD-0182050-2121709 | 1KP |
| Lamiales | Orobanchaceae | *Lindenbergia philippensis* | WUZV-0071158-2063061 | 1KP |
| Lamiales | Orobanchaceae | *Lindenbergia philippensis* | WUZV-2063264-0019871 | 1KP |
| Lamiales | Orobanchaceae | *Epifagus virginiana* | XMOG-0054357-2059542-0044951 | 1KP |
| Lamiales | Paulowniaceae | *Paulownia fargesii* | UMUL-2000866 | 1KP |
| Lamiales | Phrymaceae | *Mimulus guttatus* | Migut.C00548.1 | Phytozome |
| Lamiales | Phrymaceae | *Mimulus guttatus* | Migut.D01555.1 | Phytozome |
| Lamiales | Phrymaceae | *Mimulus guttatus* | Migut.D02160.1 | Phytozome |
| Lamiales | Phrymaceae | *Mimulus guttatus* | Migut.F00701.1 | Phytozome |
| Lamiales | Phrymaceae | *Mimulus guttatus* | Migut.F01969.1 | Phytozome |
| Lamiales | Phrymaceae | *Mimulus guttatus* | Migut.H02166.1 | Phytozome |
| Lamiales | Phrymaceae | *Mimulus guttatus* | Migut.H02206.1 | Phytozome |
| Lamiales | Phrymaceae | *Mimulus guttatus* | Migut.I00308.1 | Phytozome |
| Lamiales | Phrymaceae | *Mimulus guttatus* | Migut.J01603.1 | Phytozome |
| Lamiales | Phrymaceae | *Mimulus guttatus* | Migut.K00205.1 | Phytozome |
| Lamiales | Phrymaceae | *Mimulus guttatus* | Migut.L00355.1 | Phytozome |

**S1 Table.** (Continued)

| Order | Family | Species | Protein | Source |
| --- | --- | --- | --- | --- |
| Lamiales | Phrymaceae | *Mimulus guttatus* | Migut.M01086.1 | Phytozome |
| Lamiales | Phrymaceae | *Mimulus guttatus* | Migut.N02641.1 | Phytozome |
| Lamiales | Phrymaceae | *Mimulus guttatus* | Migut.N02736.1 | Phytozome |
| Lamiales | Plantaginaceae | *Antirrhinum majus* | EBOL-2056386-0019953 | 1KP |
| Lamiales | Plantaginaceae | *Antirrhinum majus* | EBOL-2057286-0072311 | 1KP |
| Lamiales | Plantaginaceae | *Antirrhinum majus* | TPUT-0068754-2008094-2008093 | 1KP |
| Lamiales | Schlegeliaceae | *Schlegelia parasitica* | CWLL-0008866-2027078 | 1KP |
| Lamiales | Schlegeliaceae | *Schlegelia parasitica* | CWLL-0219826-2013063 | 1KP |
| Lamiales | Schlegeliaceae | *Schlegelia parasitica* | CWLL-2027077-0026794 | 1KP |
| Lamiales | Schlegeliaceae | *Schlegelia parasitica* | GAKQ-0092627-2014301-0073443 | 1KP |
| Lamiales | Schlegeliaceae | *Schlegelia parasitica* | GAKQ-2013530-0006446 | 1KP |
| Lamiales | Schlegeliaceae | *Schlegelia violacea* | EDXZ-0031388-2090703 | 1KP |
| Lamiales | Schlegeliaceae | *Schlegelia violacea* | EDXZ-0032271-2013851 | 1KP |
| Lamiales | Schlegeliaceae | *Schlegelia violacea* | EDXZ-0101259-2087104-0108245 | 1KP |
| Lamiales | Schlegeliaceae | *Schlegelia violacea* | EDXZ-0116329-2085479 | 1KP |
| Lamiales | Schlegeliaceae | *Schlegelia violacea* | EDXZ-0118691-2089506 | 1KP |
| Lamiales | Schlegeliaceae | *Schlegelia violacea* | EDXZ-2008001-0092191 | 1KP |
| Lamiales | Schlegeliaceae | *Schlegelia violacea* | EDXZ-2015061-0029544 | 1KP |
| Lamiales | Verbenaceae | *Verbena hastata* | GCFE-2046915 | 1KP |
| Lamiales | Verbenaceae | *Verbena hastata* | GCFE-2047773 | 1KP |
| Lamiales | Verbenaceae | *Verbena hastata* | GCFE-2048933 | 1KP |
| Lamiales | Verbenaceae | *Verbena hastata* | GCFE-2053289 | 1KP |
| Laurales | Calycanthaceae | *Idiospermum australiense* | WPHN-0012900-2013160 | 1KP |
| Laurales | Calycanthaceae | *Idiospermum australiense* | WPHN-0024968-2013179 | 1KP |
| Laurales | Calycanthaceae | *Idiospermum australiense* | WPHN-0059065-2010449-0018223 | 1KP |
| Laurales | Calycanthaceae | *Idiospermum australiense* | WPHN-0065440-2004942-0039710 | 1KP |
| Laurales | Lauraceae | *Persea borbonia* | WIGA-2003749 | 1KP |
| Laurales | Lauraceae | *Persea borbonia* | WIGA-2005902-0024929 | 1KP |

**S1 Table.** (Continued)

| Order | Family | Species | Protein | Source |
| --- | --- | --- | --- | --- |
| Liliales | Melanthiaceae | *Helonias bullata* | OOSO-0026917-2005260 | 1KP |
| Liliales | Melanthiaceae | *Helonias bullata* | OOSO-0128575-2082621 | 1KP |
| Magnoliales | Eupomatiaceae | *Eupomatia bennettii* | DHPO-0013484-2075678 | 1KP |
| Malpighiales | Euphorbiaceae | *Manihot esculenta* | cassava4.1_014347m | Phytozome |
| Malpighiales | Euphorbiaceae | *Manihot esculenta* | Cassava4.1_015445m | Phytozome |
| Malpighiales | Euphorbiaceae | *Manihot esculenta* | cassava4.1_016031m | Phytozome |
| Malpighiales | Euphorbiaceae | *Manihot esculenta* | cassava4.1_017042m | Phytozome |
| Malpighiales | Euphorbiaceae | *Manihot esculenta* | cassava4.1_021403m | Phytozome |
| Malpighiales | Euphorbiaceae | *Manihot esculenta* | cassava4.1_022810m | Phytozome |
| Malpighiales | Euphorbiaceae | *Manihot esculenta* | cassava4.1_023410m | Phytozome |
| Malpighiales | Euphorbiaceae | *Manihot esculenta* | cassava4.1_023602m | Phytozome |
| Malpighiales | Euphorbiaceae | *Manihot esculenta* | cassava4.1_023871m | Phytozome |
| Malpighiales | Euphorbiaceae | *Manihot esculenta* | cassava4.1_026231m | Phytozome |
| Malpighiales | Euphorbiaceae | *Manihot esculenta* | cassava4.1_028209m | Phytozome |
| Malpighiales | Euphorbiaceae | *Manihot esculenta* | cassava4.1_029129m | Phytozome |
| Malpighiales | Euphorbiaceae | *Manihot esculenta* | cassava4.1_030340m | Phytozome |
| Malpighiales | Euphorbiaceae | *Manihot esculenta* | cassava4.1_031567m | Phytozome |
| Malpighiales | Euphorbiaceae | *Manihot esculenta* | cassava4.1_031727m | Phytozome |
| Malpighiales | Euphorbiaceae | *Manihot esculenta* | cassava4.1_031877m | Phytozome |
| Malpighiales | Euphorbiaceae | *Manihot esculenta* | cassava4.1_033579m | Phytozome |
| Malpighiales | Euphorbiaceae | *Manihot esculenta* | cassava4.1_033740m | Phytozome |
| Malpighiales | Euphorbiaceace | *Ricinus communis* | 27471.m000409 | Phytozome |
| Malpighiales | Euphorbiaceace | *Ricinus communis* | 28102.m000106 | Phytozome |
| Malpighiales | Euphorbiaceace | *Ricinus communis* | 29669.m000805 | Phytozome |
| Malpighiales | Euphorbiaceace | *Ricinus communis* | 29816.m000691 | Phytozome |
| Malpighiales | Euphorbiaceace | *Ricinus communis* | 29830.m001452 | Phytozome |
| Malpighiales | Euphorbiaceace | *Ricinus communis* | 29876.m000245 | Phytozome |
| Malpighiales | Euphorbiaceace | *Ricinus communis* | 29900.m001555 | Phytozome |
| Malpighiales | Euphorbiaceace | *Ricinus communis* | 30039.m000243 | Phytozome |
| Malpighiales | Euphorbiaceace | *Ricinus communis* | 30072.m000927 | Phytozome |
| Malpighiales | Euphorbiaceace | *Ricinus communis* | 30170.m013846 | Phytozome |
| Malpighiales | Euphorbiaceace | *Ricinus communis* | 30170.m014389 | Phytozome |
| Malpighiales | Linaceae | *Linum usitatissimum* | Lus10005282 | Phytozome |
| Malpighiales | Linaceae | *Linum usitatissimum* | Lus10012250 | Phytozome |
| Malpighiales | Linaceae | *Linum usitatissimum* | Lus10013960 | Phytozome |
| Malpighiales | Linaceae | *Linum usitatissimum* | Lus10016026 | Phytozome |

**S1 Table.** (Continued)

| Order | Family | Species | Protein | Source |
| --- | --- | --- | --- | --- |
| Malpighiales | Linaceae | *Linum usitatissimum* | Lus10016569 | Phytozome |
| Malpighiales | Linaceae | *Linum usitatissimum* | Lus10018011 | Phytozome |
| Malpighiales | Linaceae | *Linum usitatissimum* | Lus10023561 | Phytozome |
| Malpighiales | Linaceae | *Linum usitatissimum* | Lus10024808 | Phytozome |
| Malpighiales | Linaceae | *Linum usitatissimum* | Lus10028271 | Phytozome |
| Malpighiales | Linaceae | *Linum usitatissimum* | Lus10028457 | Phytozome |
| Malpighiales | Linaceae | *Linum usitatissimum* | Lus10038480 | Phytozome |
| Malpighiales | Linaceae | *Linum usitatissimum* | Lus10040219 | Phytozome |
| Malpighiales | Linaceae | *Linum usitatissimum* | Lus10040447 | Phytozome |
| Malpighiales | Linaceae | *Linum usitatissimum* | Lus10040840 | Phytozome |
| Malpighiales | Linaceae | *Linum usitatissimum* | Lus10041909 | Phytozome |
| Malpighiales | Linaceae | *Linum usitatissimum* | Lus10042007 | Phytozome |
| Malpighiales | Malpighiaceae | *Galphimia gracilis* | XPBC-0006234-2040691 | 1KP |
| Malpighiales | Malpighiaceae | *Galphimia gracilis* | XPBC-0016725-2096316 | 1KP |
| Malpighiales | Malpighiaceae | *Galphimia gracilis* | XPBC-2093013-0048205-2060160-0033286-0098187-2098558-2000365 | 1KP |
| Malpighilales | Ochnaceae | *Ochna serrulata* | CKDK-0021117-2001815 | 1KP |
| Malpighilales | Ochnaceae | *Ochna serrulata* | CKDK-0037746-2091567 | 1KP |
| Malpighilales | Ochnaceae | *Ochna serrulata* | CKDK-0131960v2024453 | 1KP |
| Malpighilales | Ochnaceae | *Ochna serrulata* | CKDK-2010965-0132816 | 1KP |
| Malpighilales | Passifloraceae | *Malesherbia fasciculata* | COAQ-0078077-2007739 | 1KP |
| Malpighilales | Passifloraceae | *Malesherbia fasciculata* | COAQ-0081651-2053558 | 1KP |
| Malpighilales | Passifloraceae | *Malesherbia fasciculata* | COAQ-2009978-0078966 | 1KP |
| Malpighiales | Passifloraceae | *Passiflora edulis* | EZZT-0013641-2015138 | 1KP |
| Malpighiales | Passifloraceae | *Passiflora edulis* | EZZT-0014008-2008387 | 1KP |
| Malpighiales | Passifloraceae | *Passiflora edulis* | EZZT-0059882-2012212-0003818 | 1KP |
| Malpighiales | Passifloraceae | *Passiflora edulis* | EZZT-2006583-0054530 | 1KP |

**S1 Table.** (Continued)

| Order | Family | Species | Protein | Source |
| --- | --- | --- | --- | --- |
| Malpighiales | Salicaceae | *Populus trichocarpa* | Potri.001G237900.1 | Phytozome |
| Malpighiales | Salicaceae | *Populus trichocarpa* | Potri.002G008800.1 | Phytozome |
| Malpighiales | Salicaceae | *Populus trichocarpa* | Potri.002G124100.1 | Phytozome |
| Malpighiales | Salicaceae | *Populus trichocarpa* | Potri.004G051600.1 | Phytozome |
| Malpighiales | Salicaceae | *Populus trichocarpa* | Potri.005G101800.1 | Phytozome |
| Malpighiales | Salicaceae | *Populus trichocarpa* | Potri.005G114700.1 | Phytozome |
| Malpighiales | Salicaceae | *Populus trichocarpa* | Potri.005G252800.1 | Phytozome |
| Malpighiales | Salicaceae | *Populus trichocarpa* | Potri.007G012100.1 | Phytozome |
| Malpighiales | Salicaceae | *Populus trichocarpa* | Potri.008G065400.1 | Phytozome |
| Malpighiales | Salicaceae | *Populus trichocarpa* | Potri.009G029200.1 | Phytozome |
| Malpighiales | Salicaceae | *Populus trichocarpa* | Potri.010G111400.1 | Phytozome |
| Malpighiales | Salicaceae | *Populus trichocarpa* | Potri.010G192100.1 | Phytozome |
| Malpighiales | Salicaceae | *Populus trichocarpa* | Potri.011G061400.1 | Phytozome |
| Malpighiales | Salicaceae | *Populus trichocarpa* | Potri.012G047700.1 | Phytozome |
| Malpighiales | Salicaceae | *Populus trichocarpa* | Potri.013G066900.1 | Phytozome |
| Malpighiales | Salicaceae | *Populus trichocarpa* | Potri.014G025300.1 | Phytozome |
| Malpighiales | Salicaceae | *Populus trichocarpa* | Potri.015G039100.1 | Phytozome |
| Malpighiales | Salicaceae | *Populus trichocarpa* | Potri.019G040800.1 | Phytozome |
| Malpighiales | Salicaceae | *Salix sachalinensis* | TDTF-2007978 | 1KP |
| Malpighiales | Salicaceae | *Salix sachalinensis* | TDTF-2023477 | 1KP |
| Malpighiales | Salicaceae | *Salix sachalinensis* | TDTF-2078644 | 1KP |
| Malpighiales | Violaceae | *Viola canadensis* | NJLF-0012768-2001053 | 1KP |
| Malpighiales | Violaceae | *Viola canadensis* | NJLF-0094375-2012308 | 1KP |
| Malpighiales | Violaceae | *Viola canadensis* | NJLF-2010134-0094227 | 1KP |
| Malpighiales | Violaceae | *Viola tricolor* | LPGY-2013195-0018237 | 1KP |
| Malpighiales | Violaceae | *Viola tricolor* | LPGY-2020346-0238988 | 1KP |
| Malpighiales | Violaceae | *Viola tricolor* | LPGY-2027772-0218614 | 1KP |
| Malpighiales | Violaceae | *Viola tricolor* | LPGY-2028855-0124583-0204975 | 1KP |

**S1 Table.** (Continued)

| Order | Family | Species | Protein | Source |
| --- | --- | --- | --- | --- |
| Malvales | Cistaceae | *Cistus inflatus* | PKMO-0067294-2020424-0184451 | 1KP |
| Malvales | Cistaceae | *Cistus inflatus* | PKMO-0182657-2023101 | 1KP |
| Malvales | Cistaceae | *Cistus inflatus* | PKMO-2022779-0064441 | 1KP |
| Malvales | Malvaceae | *Gossypium raimondii* | Gorai.001G108900.1 | Phytozome |
| Malvales | Malvaceae | *Gossypium raimondii* | Gorai.001G199200.1 | Phytozome |
| Malvales | Malvaceae | *Gossypium raimondii* | Gorai.002G134800.1 | Phytozome |
| Malvales | Malvaceae | *Gossypium raimondii* | Gorai.002G178400.1 | Phytozome |
| Malvales | Malvaceae | *Gossypium raimondii* | Gorai.003G002200.1 | Phytozome |
| Malvales | Malvaceae | *Gossypium raimondii* | Gorai.003G138900.1 | Phytozome |
| Malvales | Malvaceae | *Gossypium raimondii* | Gorai.004G038300.1 | Phytozome |
| Malvales | Malvaceae | *Gossypium raimondii* | Gorai.005G101400.1 | Phytozome |
| Malvales | Malvaceae | *Gossypium raimondii* | Gorai.007G346300.1 | Phytozome |
| Malvales | Malvaceae | *Gossypium raimondii* | Gorai.007G372000.1 | Phytozome |
| Malvales | Malvaceae | *Gossypium raimondii* | Gorai.008G063200.1 | Phytozome |
| Malvales | Malvaceae | *Gossypium raimondii* | Gorai.008G289800.1 | Phytozome |
| Malvales | Malvaceae | *Gossypium raimondii* | Gorai.009G103500.1 | Phytozome |
| Malvales | Malvaceae | *Gossypium raimondii* | Gorai.009G165700.1 | Phytozome |
| Malvales | Malvaceae | *Gossypium raimondii* | Gorai.009G214200.1 | Phytozome |
| Malvales | Malvaceae | *Gossypium raimondii* | Gorai.011G031300.1 | Phytozome |
| Malvales | Malvaceae | *Gossypium raimondii* | Gorai.011G098600.1 | Phytozome |
| Malvales | Malvaceae | *Gossypium raimondii* | Gorai.011G283000.1 | Phytozome |
| Malvales | Malvaceae | *Gossypium raimondii* | Gorai.013G196400.1 | Phytozome |
| Malvales | Malvaceae | *Gossypium raimondii* | Gorai.013G264200.1 | Phytozome |
| Malvales | Malvaceae | *Theobroma cacao* | Thecc1EG000062t2 | Phytozome |
| Malvales | Malvaceae | *Theobroma cacao* | Thecc1EG000215t1 | Phytozome |
| Malvales | Malvaceae | *Theobroma cacao* | Thecc1EG010888t2 | Phytozome |
| Malvales | Malvaceae | *Theobroma cacao* | Thecc1EG012755t2 | Phytozome |

**S1 Table.** (Continued)

| Order | Family | Species | Protein | Source |
| --- | --- | --- | --- | --- |
| Malvales | Malvaceae | *Theobroma cacao* | Thecc1EG025805t1 | Phytozome |
| Malvales | Malvaceae | *Theobroma cacao* | Thecc1EG029776t1 | Phytozome |
| Malvales | Malvaceae | *Theobroma cacao* | Thecc1EG033793t1 | Phytozome |
| Malvales | Malvaceae | *Theobroma cacao* | Thecc1EG034409t1 | Phytozome |
| Malvales | Malvaceae | *Theobroma cacao* | Thecc1EG041866t1 | Phytozome |
| Malvales | Malvaceae | *Theobroma cacao* | Thecc1EG041963t1 | Phytozome |
| Malvales | Malvaceae | *Theobroma cacao* | Thecc1EG042593t1 | Phytozome |
| Malvales | Thymelaeaceae | *Edgeworthia papyrifera* | AWJM-0017546-2012079 | 1KP |
| Malvales | Thymelaeaceae | *Edgeworthia papyrifera* | AWJM-2071403 | 1KP |
| Malvales | Thymelaeaceae | *Edgeworthia papyrifera* | AWJM-2071576-0025816 | 1KP |
| Malvales | Thymelaeaceae | *Wikstroemia indica* | QJXB-0014092-2009577 | 1KP |
| Malvales | Thymelaeaceae | *Wikstroemia indica* | QJXB-0018836-2014142 | 1KP |
| Malvales | Thymelaeaceae | *Wikstroemia indica* | QJXB-0089340-2059490 | 1KP |
| Malvales | Thymelaeaceae | *Wikstroemia indica* | QJXB-0090522-2060238 | 1KP |
| Myrtales | Melastomataceae | *Medinilla magnifica* | WWQZ-0006001 | 1KP |
| Myrtales | Melastomataceae | *Medinilla magnifica* | WWQZ-0009140-2057246 | 1KP |
| Myrtales | Melastomataceae | *Medinilla magnifica* | WWQZ-2007207-0087911-0164920 | 1KP |
| Myrtales | Melastomataceae | *Medinilla magnifica* | WWQZ-2013256 | 1KP |
| Myrtales | Melastomataceae | *Medinilla magnifica* | WWQZ-2115288 | 1KP |
| Myrtales | Lythraceae | *Punica granatum* | QEBC-0019639-2050407 | 1KP |
| Myrtales | Lythraceae | *Punica granatum* | QEBC-2050422-0018783 | 1KP |
| Myrtales | Lythraceae | *Punica granatum* | YMUO-0019635-2054909 | 1KP |
| Myrtales | Lythraceae | *Punica granatum* | YMUO-0093602-2053548 | 1KP |
| Myrtales | Myrtaceae | *Eucalyptus grandis* | Eucgr.A02149.1 | Phytozome |
| Myrtales | Myrtaceae | *Eucalyptus grandis* | Eucgr.B00945.1 | Phytozome |
| Myrtales | Myrtaceae | *Eucalyptus grandis* | Eucgr.B02379.1 | Phytozome |
| Myrtales | Myrtaceae | *Eucalyptus grandis* | Eucgr.B02435.1 | Phytozome |
| Myrtales | Myrtaceae | *Eucalyptus grandis* | Eucgr.D00577.1 | Phytozome |
| Myrtales | Myrtaceae | *Eucalyptus grandis* | Eucgr.F02320.1 | Phytozome |
| Myrtales | Myrtaceae | *Eucalyptus grandis* | Eucgr.G03036.1 | Phytozome |
| Myrtales | Myrtaceae | *Eucalyptus grandis* | Eucgr.I02222.1 | Phytozome |

**S1 Table.** (Continued)

| Order | Family | Species | Protein | Source |
| --- | --- | --- | --- | --- |
| Myrtales | Myrtaceae | *Eucalyptus grandis* | Eucgr.J02429.1 | Phytozome |
| Myrtales | Onagraceae | *Epilobium* sp. | FEDW-0019446-2020595-0074251-0051752 | 1KP |
| Myrtales | Onagraceae | *Epilobium* sp. | FEDW-0037002-2090588 | 1KP |
| Nymphaeales | Nymphaeaceae | *Nuphar advena* | WTKZ-0031478-0095242-2011689-0051223 | 1KP |
| Oxalidales | Oxalidaceae | *Oxalis* sp. | JHCN-2014604-0111788 | 1KP |
| Oxalidales | Oxalidaceae | *Oxalis* sp. | JHCN-2020771-0002220 | 1KP |
| Pandanales | Cyclanthaceae | *Ludovia* sp. | VVVV-0065849-2042119 | 1KP |
| Piperales | Piperaceae | *Peperomia fraseri* | XSZI-2011419 | 1KP |
| Piperales | Piperaceae | *Peperomia fraseri* | XSZI-2049852 | 1KP |
| Piperales | Piperaceae | *Peperomia fraseri* | XSZI-2051147 | 1KP |
| Poales | Bromeliaceae | *Ananas comosus* | Aco001509.1 | Phytozome |
| Poales | Bromeliaceae | *Ananas comosus* | Aco001683.1 | Phytozome |
| Poales | Bromeliaceae | *Ananas comosus* | Aco007021.1 | Phytozome |
| Poales | Bromeliaceae | *Ananas comosus* | Aco011844.1 | Phytozome |
| Poales | Bromeliaceae | *Ananas comosus* | Aco013026.1 | Phytozome |
| Poales | Bromeliaceae | *Ananas comosus* | Aco015382.1 | Phytozome |
| Poales | Bromeliaceae | *Ananas comosus* | Aco015463.1 | Phytozome |
| Poales | Bromeliaceae | *Ananas comosus* | Aco017897.1 | Phytozome |
| Poales | Bromeliaceae | *Ananas comosus* | Aco022929.1 | Phytozome |
| Poales | Bromeliaceae | *Ananas comosus* | Aco029104.1 | Phytozome |
| Poales | Bromeliaceae | *Brocchinia reducta* | BYPY-0027058-2009804 | 1KP |
| Poales | Bromeliaceae | *Brocchinia reducta* | BYPY-0093037-2011797 | 1KP |
| Poales | Bromeliaceae | *Brocchinia reducta* | BYPY-0094645-2076520 | 1KP |
| Poales | Bromeliaceae | *Brocchinia reducta* | BYPY-2004579 | 1KP |
| Poales | Joinvilleaceae | *Joinvillea ascendens* | WXNT-0070608-2003959 | 1KP |
| Poales | Cyperaceae | *Lepidosperma gibsonii* | WBIB-0021393-2010311 | 1KP |
| Poales | Cyperaceae | *Lepidosperma gibsonii* | WBIB-0087389-2009321 | 1KP |
| Poales | Cyperaceae | *Lepidosperma gibsonii* | WBIB-2063693-0024795 | 1KP |
| Poales | Poaceae | *Oryza sativa* | LOC_Os01g47710.1 | Phytozome |
| Poales | Poaceae | *Oryza sativa* | LOC_Os01g60270.2 | Phytozome |
| Poales | Poaceae | *Oryza sativa* | LOC_Os01g62310.1 | Phytozome |
| Poales | Poaceae | *Oryza sativa* | LOC_Os01g63510.1 | Phytozome |

**S1 Table.** (Continued)

| Order | Family | Species | Protein | Source |
| --- | --- | --- | --- | --- |
| Poales | Poaceae | *Oryza sativa* | LOC_Os03g20910.2 | Phytozome |
| Poales | Poaceae | *Oryza sativa* | LOC_Os04g55590.1 | Phytozome |
| Poales | Poaceae | *Oryza sativa* | LOC_Os04g56780.1 | Phytozome |
| Poales | Poaceae | *Oryza sativa* | LOC_Os05g02730.1 | Phytozome |
| Poales | Poaceae | *Oryza sativa* | LOC_Os05g48990.1 | Phytozome |
| Poales | Poaceae | *Oryza sativa* | LOC_Os07g34880.1 | Phytozome |
| Poales | Poaceae | *Oryza sativa* | LOC_Os07g48560.1 | Phytozome |
| Poales | Poaceae | *Oryza sativa* | LOC_Os08g14400.1 | Phytozome |
| Poales | Poaceae | *Oryza sativa* | LOC_Os11g01130.2 | Phytozome |
| Poales | Poaceae | *Oryza sativa* | LOC_Os12g01120.1 | Phytozome |
| Poales | Poaceae | *Panicum virgatum* | Pavir.Ba00249.1 | Phytozome |
| Poales | Poaceae | *Panicum virgatum* | Pavir.Ca00701.1 | Phytozome |
| Poales | Poaceae | *Panicum virgatum* | Pavir.Ca00938.1 | Phytozome |
| Poales | Poaceae | *Panicum virgatum* | Pavir.Ea03245.1 | Phytozome |
| Poales | Poaceae | *Panicum virgatum* | Pavir.Ea03339.1 | Phytozome |
| Poales | Poaceae | *Panicum virgatum* | Pavir.Ea03790.1 | Phytozome |
| Poales | Poaceae | *Panicum virgatum* | Pavir.Eb02702.1 | Phytozome |
| Poales | Poaceae | *Panicum virgatum* | Pavir.Eb03552.1 | Phytozome |
| Poales | Poaceae | *Panicum virgatum* | Pavir.Eb03715.1 | Phytozome |
| Poales | Poaceae | *Panicum virgatum* | Pavir.Fb01029.1 | Phytozome |
| Poales | Poaceae | *Panicum virgatum* | Pavir.Ga00150.1 | Phytozome |
| Poales | Poaceae | *Panicum virgatum* | Pavir.Ga00299.1 | Phytozome |
| Poales | Poaceae | *Panicum virgatum* | Pavir.Gb00125.1 | Phytozome |
| Poales | Poaceae | *Panicum virgatum* | Pavir.Ia03448.1 | Phytozome |
| Poales | Poaceae | *Panicum virgatum* | Pavir.Ib01521.1 | Phytozome |
| Poales | Poaceae | *Panicum virgatum* | Pavir.J05483.1 | Phytozome |
| Poales | Poaceae | *Panicum virgatum* | Pavir.J15457.1 | Phytozome |
| Poales | Poaceae | *Panicum virgatum* | Pavir.J22602.1 | Phytozome |
| Poales | Poaceae | *Panicum virgatum* | Pavir.J23422.1 | Phytozome |
| Poales | Poaceae | *Panicum virgatum* | Pavir.J24082.1 | Phytozome |
| Poales | Poaceae | *Panicum virgatum* | Pavir.J33010.1 | Phytozome |
| Poales | Poaceae | *Panicum virgatum* | Pavir.J34036.1 | Phytozome |
| Poales | Poaceae | *Panicum virgatum* | Pavir.J34412.1 | Phytozome |
| Poales | Poaceae | *Panicum virgatum* | Pavir.J36471.1 | Phytozome |
| Poales | Restionaceae | *Chondropetalum tectorum* | BSTR-0018580-0039612-2032544-2011492 | 1KP |
| Poales | Typhaceae | *Typha latifolia* | BRUD-0002843-2062742 | 1KP |
| Poales | Typhaceae | *Typha latifolia* | BRUD-0008472-2060273 | 1KP |

**S1 Table.** (Continued)

| Order | Family | Species | Protein | Source |
| --- | --- | --- | --- | --- |
| Primulales | Primulaceae | *Ardisia revoluta* | DAAD-0014615-2040098 | 1KP |
| Primulales | Primulaceae | *Ardisia revoluta* | DAAD-0054667-2036919 | 1KP |
| Proteales | Nelumbonaceae | *Nelumbo nucifera* | NNU_000148-RA | CoGe |
| Proteales | Nelumbonaceae | *Nelumbo nucifera* | NNU_002079-RA | CoGe |
| Proteales | Nelumbonaceae | *Nelumbo nucifera* | NNU_005564-RA | CoGe |
| Proteales | Nelumbonaceae | *Nelumbo nucifera* | NNU_007474-RA | CoGe |
| Proteales | Nelumbonaceae | *Nelumbo nucifera* | NNU_009436-RA | CoGe |
| Proteales | Nelumbonaceae | *Nelumbo nucifera* | NNU_009452-RA | CoGe |
| Proteales | Nelumbonaceae | *Nelumbo nucifera* | NNU_009711-RA | CoGe |
| Proteales | Nelumbonaceae | *Nelumbo nucifera* | NNU_011213-RA | CoGe |
| Proteales | Nelumbonaceae | *Nelumbo nucifera* | NNU_011594-RA | CoGe |
| Proteales | Nelumbonaceae | *Nelumbo nucifera* | NNU_013352-RA | CoGe |
| Proteales | Nelumbonaceae | *Nelumbo nucifera* | NNU_013689-RA | CoGe |
| Proteales | Nelumbonaceae | *Nelumbo nucifera* | NNU_014892-RA | CoGe |
| Proteales | Nelumbonaceae | *Nelumbo nucifera* | NNU_015981-RA | CoGe |
| Proteales | Nelumbonaceae | *Nelumbo nucifera* | NNU_018779-RA | CoGe |
| Proteales | Nelumbonaceae | *Nelumbo nucifera* | NNU_021629-RA | CoGe |
| Proteales | Platanaceae | *Platanus occidentalis* | VQFW-0023594-2005649 | 1KP |
| Proteales | Platanaceae | *Platanus occidentalis* | VQFW-0025371-2012801 | 1KP |
| Proteales | Platanaceae | *Platanus occidentalis* | VQFW-0062646-2062140 | 1KP |
| Proteales | Proteaceae | *Hakea prostrata* | OBOJ-0017091-2000708-0094327 | 1KP |
| Proteales | Proteaceae | *Hakea prostrata* | OBOJ-0018069-2007405 | 1KP |
| Proteales | Proteaceae | *Hakea prostrata* | OBOJ-0112698-2000707-0094352 | 1KP |
| Proteales | Proteaceae | *Hakea prostrata* | REOF-0020342-2073018 | 1KP |
| Proteales | Proteaceae | *Hakea prostrata* | REOF-0020805-2101972 | 1KP |
| Proteales | Proteaceae | *Hakea prostrata* | REOF-0113151-2008873-0103717 | 1KP |
| Proteales | Proteaceae | *Hakea prostrata* | REOF-0143687-2101575 | 1KP |
| Proteales | Proteaceae | *Hakea prostrata* | SIIK-0030209-2000193 | 1KP |
| Proteales | Proteaceae | *Hakea prostrata* | SIIK-0103847-2007829-0110203 | 1KP |
| Proteales | Proteaceae | *Hakea prostrata* | SIIK-0110194-2082100 | 1KP |
| Proteales | Proteaceae | *Hakea prostrata* | SIIK-0110513 | 1KP |
| Proteales | Proteaceae | *Hakea prostrata* | SIIK-2081174 | 1KP |
| Proteales | Sabiaceae | *Meliosma cuneifolia* | AALA-0010172-2012269 | 1KP |
| Proteales | Sabiaceae | *Meliosma cuneifolia* | AALA-2010508-0107166 | 1KP |

**S1 Table.** (Continued)

| Order | Family | Species | Protein | Source |
| --- | --- | --- | --- | --- |
| Proteales | Sabiaceae | *Meliosma cuneifolia* | AALA-2012270-0091586 | 1KP |
| Proteales | Sabiaceae | *Meliosma cuneifolia* | AALA-2059109-0108586-2010169 | 1KP |
| Ranunculales | Berberidaceae | *Podophyllum peltatum* | WFBF-0045809-2040117 | 1KP |
| Ranunculales | Berberidaceae | *Podophyllum peltatum* | WFBF-0048199-2042753 | 1KP |
| Ranunculales | Eupteleaceae | *Euptelea pleiosperma* | QTJY-0012057-2017539-2033883-2005550 | 1KP |
| Ranunculales | Eupteleaceae | *Euptelea pleiosperma* | QTJY-0014482-2053776 | 1KP |
| Ranunculales | Eupteleaceae | *Euptelea pleiosperma* | QTJY-0019746-2014547 | 1KP |
| Ranunculales | Eupteleaceae | *Euptelea pleiosperma* | QTJY-0052316-2005549 | 1KP |
| Ranunculales | Eupteleaceae | *Euptelea pleiosperma* | QTJY-0079730-2014546-0082573 | 1KP |
| Ranunculales | Ranunculaceae | *Aquilegia coerulea* | Aquca-004-00311.1 | Phytozome |
| Ranunculales | Ranunculaceae | *Aquilegia coerulea* | Aquca-004-00664.1 | Phytozome |
| Ranunculales | Ranunculaceae | *Aquilegia coerulea* | Aquca-007-00901.1 | Phytozome |
| Ranunculales | Ranunculaceae | *Aquilegia coerulea* | Aquca-010-00626.1 | Phytozome |
| Ranunculales | Ranunculaceae | *Aquilegia coerulea* | Aquca-014-00949.1 | Phytozome |
| Ranunculales | Ranunculaceae | *Aquilegia coerulea* | Aquca-015-00333.1 | Phytozome |
| Ranunculales | Ranunculaceae | *Aquilegia coerulea* | Aquca-027-00327.1 | Phytozome |
| Ranunculales | Ranunculaceae | *Aquilegia coerulea* | Aquca-036-00007.1 | Phytozome |
| Ranunculales | Ranunculaceae | *Aquilegia coerulea* | Aquca-046-00011.1 | Phytozome |
| Ranunculales | Ranunculaceae | *Aquilegia coerulea* | Aquca-091-00003.1 | Phytozome |
| Ranunculales | Ranunculaceae | *Cimicifuga racemosa* | CYVA-2069069 | 1KP |
| Ranunculales | Ranunculaceae | *Cimicifuga racemosa* | CYVA-2073570 | 1KP |
| Ranunculales | Ranunculaceae | *Cimicifuga racemosa* | CYVA-2073886 | 1KP |
| Ranunculales | Ranunculaceae | *Cimicifuga racemosa* | CYVA-2074200 | 1KP |
| Ranunculales | Ranunculaceae | *Hydrastis canadensis* | VGHH-0058294-2050062-0075435 | 1KP |
| Ranunculales | Ranunculaceae | *Hydrastis canadensis* | VGHH-0064729-2048195-0040109 | 1KP |
| Ranunculales | Papaveraceae | *Eschscholzia californica* | ERXG-0006394-2017962 | 1KP |
| Ranunculales | Papaveraceae | *Eschscholzia californica* | ERXG-0008725-2064605-0067163 | 1KP |
| Ranunculales | Papaveraceae | *Eschscholzia californica* | NJKC-0008378-2007920 | 1KP |
| Ranunculales | Papaveraceae | *Eschscholzia californica* | NJKC-0010480-2013864 | 1KP |

**S1 Table.** (Continued)

| Order | Family | Species | Protein | Source |
| --- | --- | --- | --- | --- |
| Ranunculales | Papaveraceae | *Eschscholzia californica* | NJKC-0024840-2057950 | 1KP |
| Ranunculales | Papaveraceae | *Eschscholzia californica* | RKGT-0076093-2058088 | 1KP |
| Ranunculales | Papaveraceae | *Eschscholzia californica* | RKGT-2003813-0070931 | 1KP |
| Ranunculales | Papaveraceae | *Eschscholzia californica* | TUHA-0008602-2019319 | 1KP |
| Ranunculales | Papaveraceae | *Eschscholzia californica* | UNPT-0067416-2055913 | 1KP |
| Ranunculales | Papaveraceae | *Eschscholzia californica* | UNPT-0070247-2035952 | 1KP |
| Rosales | Moraceae | *Morus nigra* | XVJB-0010886-2052515 | 1KP |
| Rosales | Moraceae | *Morus nigra* | XVJB-2001572-0001312-2001573 | 1KP |
| Rosales | Moraceae | *Morus nigra* | XVJB-2053948-0083428 | 1KP |
| Rosales | Rosaceae | *Fragaria vesca* | mrna09136.1-v1.0-hybrid | Phytozome |
| Rosales | Rosaceae | *Fragaria vesca* | mrna09389.1-v1.0-hybrid | Phytozome |
| Rosales | Rosaceae | *Fragaria vesca* | mrna13035.1-v1.0-hybrid | Phytozome |
| Rosales | Rosaceae | *Fragaria vesca* | mrna14025.1-v1.0-hybrid | Phytozome |
| Rosales | Rosaceae | *Fragaria vesca* | mrna14133.1-v1.0-hybrid | Phytozome |
| Rosales | Rosaceae | *Fragaria vesca* | mrna14621.1-v1.0-hybrid | Phytozome |
| Rosales | Rosaceae | *Fragaria vesca* | mrna20491.1-v1.0-hybrid | Phytozome |
| Rosales | Rosaceae | *Fragaria vesca* | mrna20925.1-v1.0-hybrid | Phytozome |
| Rosales | Rosaceae | *Fragaria vesca* | mrna28935.1-v1.0-hybrid | Phytozome |
| Rosales | Rosaceae | *Fragaria vesca* | mrna30337.1-v1.0-hybrid | Phytozome |
| Rosales | Rosaceae | *Fragaria vesca* | mrna30464.1-v1.0-hybrid | Phytozome |
| Rosales | Rosaceae | *Fragaria vesca* | mrna31986.1-v1.0-hybrid | Phytozome |
| Rosales | Rosaceae | *Fragaria vesca* | mrna32456.1-v1.0-hybrid | Phytozome |
| Rosales | Rosaceae | *Malus domestica* | MDP0000136426 | Phytozome |
| Rosales | Rosaceae | *Malus domestica* | MDP0000163159 | Phytozome |
| Rosales | Rosaceae | *Malus domestica* | MDP0000212434 | Phytozome |
| Rosales | Rosaceae | *Malus domestica* | MDP0000213910 | Phytozome |
| Rosales | Rosaceae | *Malus domestica* | MDP0000232004 | Phytozome |
| Rosales | Rosaceae | *Malus domestica* | MDP0000394158 | Phytozome |
| Rosales | Rosaceae | *Malus domestica* | MDP0000445859 | Phytozome |

**S1 Table.** (Continued)

| Order | Family | Species | Protein | Source |
| --- | --- | --- | --- | --- |
| Rosales | Rosaceae | *Malus domestica* | MDP0000600720 | Phytozome |
| Rosales | Rosaceae | *Malus domestica* | MDP0000851699 | Phytozome |
| Rosales | Urticaceae | *Boehmeria nivea* | ACFP-0004197-2004977 | 1KP |
| Rosales | Urticaceae | *Boehmeria nivea* | ACFP-0056883-2031985 | 1KP |
| Santalales | Balanophoraceae | *Balanophora fungosa* | STKY-2007431-0039830-0079836 | 1KP |
| Santalales | Loranthaceae | *Dendropemon caribaeus* | VGVI-0022570-2013205 | 1KP |
| Santalales | Loranthaceae | *Dendropemon caribaeus* | VGVI-0081005-2002451-0021711 | 1KP |
| Santalales | Loranthaceae | *Dendropemon caribaeus* | VGVI-2066756-0063981-0000722 | 1KP |
| Santalales | Olacaceae | *Ximenia americana* | XKPS-2022589-0178496 | 1KP |
| Santalales | Santalaceae | *Exocarpos cupressiformis* | XGFU-0015943-2018782 | 1KP |
| Santalales | Santalaceae | *Exocarpos cupressiformis* | XGFU-0209295-2020534 | 1KP |
| Santalales | Santalaceae | *Phoradendron serotinum* | QKMG-0094841-2006206-0037848-0037650 | 1KP |
| Sapindales | Anacardiaceae | *Rhus radicans* | YUOM-0010433-2003167 | 1KP |
| Sapindales | Anacardiaceae | *Rhus radicans* | YUOM-0045844-2037812 | 1KP |
| Sapindales | Meliaceae | *Azadirachta indica* | UVDC-0009995-0025294-2016439-2002511 | 1KP |
| Sapindales | Meliaceae | *Azadirachta indica* | UVDC-0031341-2007189-0056852 | 1KP |
| Sapindales | Meliaceae | *Azadirachta indica* | UVDC-0055566-2039994-0038644 | 1KP |
| Sapindales | Meliaceae | *Azadirachta indica* | UVDC-0059125-2037644 | 1KP |
| Sapindales | Rutaceae | *Citrus sinensis* | orange1.1g022516m | Phytozome |
| Sapindales | Rutaceae | *Citrus sinensis* | orange1.1g022558m | Phytozome |
| Sapindales | Rutaceae | *Citrus sinensis* | orange1.1g022926m | Phytozome |
| Sapindales | Rutaceae | *Citrus sinensis* | orange1.1g022944m | Phytozome |
| Sapindales | Rutaceae | *Citrus sinensis* | orange1.1g023479m | Phytozome |
| Sapindales | Rutaceae | *Citrus sinensis* | orange1.1g026809m | Phytozome |
| Sapindales | Rutaceae | *Citrus sinensis* | orange1-1g027068m | Phytozome |
| Sapindales | Rutaceae | *Citrus sinensis* | orange1-1g027250m | Phytozome |
| Sapindales | Rutaceae | *Citrus sinensis* | orange1.1g027928m | Phytozome |
| Sapindales | Rutaceae | *Citrus sinensis* | orange1.1g028506m | Phytozome |
| Sapindales | Rutaceae | *Citrus sinensis* | orange1.1g031304m | Phytozome |
| Sapindales | Rutaceae | *Citrus sinensis* | orange1.1g035594m | Phytozome |
| Sapindales | Rutaceae | *Citrus sinensis* | orange1.1g036145m | Phytozome |

**S1 Table.** (Continued)

| Order | Family | Species | Protein | Source |
| --- | --- | --- | --- | --- |
| Sapindales | Rutaceae | *Citrus sinensis* | orange1.1g036802m | Phytozome |
| Sapindales | Rutaceae | *Citrus sinensis* | orange1.1g039144m | Phytozome |
| Sapindales | Rutaceae | *Citrus sinensis* | orange1.1g043602m | Phytozome |
| Sapindales | Rutaceae | *Citrus sinensis* | orange1.1g046372m | Phytozome |
| Sapindales | Rutaceae | *Citrus sinensis* | orange1.1g046813m | Phytozome |
| Sapindales | Rutaceae | *Citrus sinensis* | orange1.1g047211m | Phytozome |
| Sapindales | Sapindaceae | *Acer negundo* | VFFP-0001350-2008450 | 1KP |
| Sapindales | Sapindaceae | *Acer negundo* | VFFP-0055846-2042543-0053042 | 1KP |
| Sapindales | Sapindaceae | *Acer negundo* | VFFP-0079522-2000573 | 1KP |
| Sapindales | Sapindaceae | *Acer negundo* | VFFP-2011714-0070214 | 1KP |
| Sapindales | Sapindaceae | *Aesculus pavia* | HBHB-0009857-0327151 | 1KP |
| Sapindales | Sapindaceae | *Aesculus pavia* | HBHB-0323017-2043899-0325683 | 1KP |
| Sapindales | Sapindaceae | *Aesculus pavia* | HBHB-2001999-0180948-0027399 | 1KP |
| Sapindales | Sapindaceae | *Aesculus pavia* | HBHB-2002638-0297902-0315952-2002639 | 1KP |
| Sapindales | Sapindaceae | *Aesculus pavia* | HBHB-2043247 | 1KP |
| Saxifragales | Cercidiphyllaceae | *Cercidiphyllum japonicum* | NUZN-0007994-2057753 | 1KP |
| Saxifragales | Crassulaceae | *Rhodiola rosea* | ZJUL-2061215 | 1KP |
| Saxifragales | Crassulaceae | *Rhodiola rosea* | ZJUL-2061737 | 1KP |
| Saxifragales | Daphniphyllaceae | *Daphniphyllum macropodum* | FYTP-0063526-2044127 | 1KP |
| Saxifragales | Daphniphyllaceae | *Daphniphyllum macropodum* | FYTP-2008231-0030200-0058939 | 1KP |
| Saxifragales | Daphniphyllaceae | *Daphniphyllum macropodum* | FYTP-2043778-0062988 | 1KP |
| Saxifragales | Daphniphyllaceae | *Daphniphyllum macropodum* | FYTP-0015435-2004066 | 1KP |
| Saxifragales | Paeoniaceae | *Paeonia lactiflora* | HTIP-0018449-2054728 | 1KP |
| Saxifragales | Paeoniaceae | *Paeonia lactiflora* | HTIP-0084280-2053767 | 1KP |
| Saxifragales | Saxifragaceae | *Heuchera sanguinea* | ERIA-0029909-2073329 | 1KP |
| Solanales | Convolvulaceae | *Ipomoea purpurea* | VXKB-0016619 | 1KP |
| Solanales | Convolvulaceae | *Ipomoea purpurea* | VXKB-0018495 | 1KP |
| Solanales | Convolvulaceae | *Ipomoea purpurea* | VXKB-0020629-2061991 | 1KP |
| Solanales | Convolvulaceae | *Ipomoea purpurea* | VXKB-2013175 | 1KP |
| Solanales | Solanaceae | *Solanum lycopersicum* | Solyc02g077390.1 | Phytozome |
| Solanales | Solanaceae | *Solanum lycopersicum* | Solyc02g082670.2 | Phytozome |

**S1 Table.** (Continued)

| Order | Family | Species | Protein | Source |
| --- | --- | --- | --- | --- |
| Solanales | Solanaceae | *Solanum lycopersicum* | Solyc02g083950.2 | Phytozome |
| Solanales | Solanaceae | *Solanum lycopersicum* | Solyc03g096300.2 | Phytozome |
| Solanales | Solanaceae | *Solanum lycopersicum* | Solyc03g118770.2 | Phytozome |
| Solanales | Solanaceae | *Solanum lycopersicum* | Solyc04g078650.2 | Phytozome |
| Solanales | Solanaceae | *Solanum lycopersicum* | Solyc06g072890.1 | Phytozome |
| Solanales | Solanaceae | *Solanum lycopersicum* | Solyc06g076000.1 | Phytozome |
| Solanales | Solanaceae | *Solanum lycopersicum* | Solyc11g072770.1 | Phytozome |
| Solanales | Solanaceae | *Solanum lycopersicum* | Solyc11g072790.1 | Phytozome |
| Trochondendronales | Trochodendronaceae | *Trochodendron araliodes* | SWOH-0015788 | 1KP |
| Trochondendronales | Trochodendronaceae | *Trochodendron araliodes* | SWOH-0077530-2033978-2010380-0057466-0079112 | 1KP |
| Vitales | Vitaceae | *Vitis vinifera* | GSVIVT01008424001 | Phytozome |
| Vitales | Vitaceae | *Vitis vinifera* | GSVIVT01009453001 | Phytozome |
| Vitales | Vitaceae | *Vitis vinifera* | GSVIVT01011738001 | Phytozome |
| Vitales | Vitaceae | *Vitis vinifera* | GSVIVT01013388001 | Phytozome |
| Vitales | Vitaceae | *Vitis vinifera* | GSVIVT01016360001 | Phytozome |
| Vitales | Vitaceae | *Vitis vinifera* | GSVIVT01018787001 | Phytozome |
| Vitales | Vitaceae | *Vitis vinifera* | GSVIVT01021144001 | Phytozome |
| Vitales | Vitaceae | *Vitis vinifera* | GSVIVT01025075001 | Phytozome |
| Vitales | Vitaceae | *Vitis vinifera* | GSVIVT01026636001 | Phytozome |
| Vitales | Vitaceae | *Vitis vinifera* | GSVIVT01026638001 | Phytozome |
| Vitales | Vitaceae | *Vitis vinifera* | GSVIVT01029942001 | Phytozome |
| Zingiberales | Lowiaceae | *Orchidantha maxillarioides* | LSKK-0026636-2013491 | 1KP |
| Zingiberales | Lowiaceae | *Orchidantha maxillarioides* | LSKK-0029366 | 1KP |
| Zingiberales | Lowiaceae | *Orchidantha maxillarioides* | LSKK-0110314-2084096 | 1KP |
| Zingiberales | Lowiaceae | *Orchidantha maxillarioides* | LSKK-2000938 | 1KP |
| Zingiberales | Lowiaceae | *Orchidantha maxillarioides* | LSKK-2004092 | 1KP |
| Zingiberales | Lowiaceae | *Orchidantha maxillarioides* | LSKK-2004312-0026123 | 1KP |
| Zingiberales | Marantaceae | *Maranta leuconeura* | JNUB-0018134-2115743 | 1KP |
| Zingiberales | Marantaceae | *Maranta leuconeura* | JNUB-0040526-2111492 | 1KP |

**S1 Table.** (Continued)

| Order | Family | Species | Protein | Source |
| --- | --- | --- | --- | --- |
| Zingiberales | Marantaceae | *Maranta leuconeura* | JNUB-2006470-0097362-0024615 | 1KP |
| Zingiberales | Musaceae | *Musa acuminata* | GSMUA_Achr10T05090_001 | Phytozome |
| Zingiberales | Musaceae | *Musa acuminata* | GSMUA_Achr10T07260_001 | Phytozome |
| Zingiberales | Musaceae | *Musa acuminata* | GSMUA_Achr10T12820_001 | Phytozome |
| Zingiberales | Musaceae | *Musa acuminata* | GSMUA_Achr10T26570_001 | Phytozome |
| Zingiberales | Musaceae | *Musa acuminata* | GSMUA_Achr1T17980_001 | Phytozome |
| Zingiberales | Musaceae | *Musa acuminata* | GSMUA_Achr2T16080_001 | Phytozome |
| Zingiberales | Musaceae | *Musa acuminata* | GSMUA_Achr3T09460_001 | Phytozome |
| Zingiberales | Musaceae | *Musa acuminata* | GSMUA_Achr3T14840_001 | Phytozome |
| Zingiberales | Musaceae | *Musa acuminata* | GSMUA_Achr4T25530_001 | Phytozome |
| Zingiberales | Musaceae | *Musa acuminata* | GSMUA_Achr4T29470_001 | Phytozome |
| Zingiberales | Musaceae | *Musa acuminata* | GSMUA_Achr4T30610_001 | Phytozome |
| Zingiberales | Musaceae | *Musa acuminata* | GSMUA_Achr5T11080_001 | Phytozome |
| Zingiberales | Musaceae | *Musa acuminata* | GSMUA_Achr5T27730_001 | Phytozome |
| Zingiberales | Musaceae | *Musa acuminata* | GSMUA_Achr5T29220_001 | Phytozome |
| Zingiberales | Musaceae | *Musa acuminata* | GSMUA_Achr6T15910_001 | Phytozome |
| Zingiberales | Musaceae | *Musa acuminata* | GSMUA_Achr7T04050_001 | Phytozome |
| Zingiberales | Musaceae | *Musa acuminata* | GSMUA_Achr7T07430_001 | Phytozome |
| Zingiberales | Musaceae | *Musa acuminata* | GSMUA_Achr7T14860_001 | Phytozome |
| Zingiberales | Musaceae | *Musa acuminata* | GSMUA_Achr7T18230_001 | Phytozome |
| Zingiberales | Musaceae | *Musa acuminata* | GSMUA_Achr8T01590_001 | Phytozome |
| Zingiberales | Musaceae | *Musa acuminata* | GSMUA_Achr8T02040_001 | Phytozome |
| Zingiberales | Musaceae | *Musa acuminata* | GSMUA_Achr8T15620_001 | Phytozome |
| Zingiberales | Musaceae | *Musa acuminata* | GSMUA_Achr8T27170_001 | Phytozome |
| Zingiberales | Musaceae | *Musa acuminata* | GSMUA_Achr9T04090_001 | Phytozome |
| Zingiberales | Musaceae | *Musa acuminata* | GSMUA_Achr9T05010_001 | Phytozome |

**S1 Table.** (Continued)

| Order | Family | Species | Protein | Source |
| --- | --- | --- | --- | --- |
| Zingiberales | Musaceae | *Musa acuminata* | GSMUA_Achr9T06660_001 | Phytozome |
| Zingiberales | Musaceae | *Musa acuminata* | GSMUA_AchrUn_randomT18610_001 | Phytozome |
| Zingiberales | Zingiberaceae | *Curcuma longa* | OYLU-0013882-2009204 | 1KP |
| Zingiberales | Zingiberaceae | *Curcuma longa* | OYLU-0129486-2094624 | 1KP |
| Zingiberales | Zingiberaceae | *Curcuma longa* | OYLU-2091435-0122842 | 1KP |
| Zingiberales | Zingiberaceae | *Zingiber officinale* | BDJQ-2015836 | 1KP |
| Zingiberales | Zingiberaceae | *Zingiber officinale* | BDJQ-2018033 | 1KP |
| Zingiberales | Zingiberaceae | *Zingiber officinale* | BDJQ-2019725 | 1KP |
| Zygophyllales | Krameriaceae | *Krameria lanceolata* | ZHMB-0106684-2080957 | 1KP |
