# Supplementary material for: Large-scale phylogenomic analysis suggests three ancient superclades of the WUSCHEL-RELATED HOMEOBOX transcription factor family in plants
Source: PLoS One. 2019 Oct 11;14(10):e0223521. doi: 10.1371/journal.pone.0223521 (PMC6788696; doi:10.1371/journal.pone.0223521)
Supplement: S2 Table — (DOCX) [file pone.0223521.s009.docx]

**S2 Table. Arabidopsis *ARF* and *B-ARR* genes used as queries for BLAST search.**

| Gene | Locus |
| --- | --- |
| *ARF1* | AT1G59750 |
| *ARF2* | AT5G62000 |
| *ARF3* | AT2G33860 |
| *ARF4* | AT5G60450 |
| *ARF5* | AT1G19850 |
| *ARF6* | AT1G30330 |
| *ARF7* | AT5G20730 |
| *ARF8* | AT5G37020 |
| *ARF9* | AT5G23980 |
| *ARF10* | AT2G28350 |
| *ARF11* | AT2G46530 |
| *ARF12* | AT1G34310 |
| *ARF13* | AT1G34170 |
| *ARF14* | AT1G35540 |
| *ARF15* | AT1G35520 |
| *ARF16* | AT4G30080 |
| *ARF17* | AT1G77850 |
| *ARF18* | AT3G61830 |
| *ARF19* | AT1G19220 |
| *ARF20* | AT1G35240 |
| *ARF21* | AT1G34410 |
| *ARF22* | AT1G34390 |
| *ARF23* | AT1G43950 |
| *ARR1* | AT3G16857 |
| *ARR2* | AT4G16110 |
| *ARR10* | AT4G31920 |
| *ARR11* | AT1G67710 |
| *ARR12* | AT2G25180 |
| *ARR13* | AT2G27070 |
| *ARR14* | AT2G01760 |
| *ARR18* | AT5G58080 |
| *ARR19* | AT1G49190 |
| *ARR20* | AT3G62670 |
| *ARR21* | AT5G07210 |
