# Supplementary material for: Large-scale phylogenomic analysis suggests three ancient superclades of the WUSCHEL-RELATED HOMEOBOX transcription factor family in plants
Source: PLoS One. 2019 Oct 11;14(10):e0223521. doi: 10.1371/journal.pone.0223521 (PMC6788696; doi:10.1371/journal.pone.0223521)
Supplement: S4 Table — (DOCX) [file pone.0223521.s011.docx]

**S4 Table. Predicted Hormone Response Elements in promoters of plant *WOX* genes.**

| Species | Locus | AuxRE | B-ARR-6-BA |
| --- | --- | --- | --- |
| *Ostreococcus lunimarinus* | eugene.1300010124 | 5 | 4 |
| *Micromonas pusilla CCMP1545* | MicpuC2.estExt_fgenesh1_pg.C_50077 | 2 | 8 |
|  | MicpuC2.estExt_fgenesh1_pg.C_90244 | 5 | 1 |
| *Micromonas sp. RCC299* | EuGene.0600010372 | 6 | 4 |
| *Marchantia polymorpha* | Mapoly0014s0060 | 4 | 24 |
| *Physcomitrella patens* | Pp3c9_13640 | 5 | 14 |
|  | Pp3c15_20000 | 2 | 19 |
|  | Pp3c26_8569 | 0 | 23 |
| *Selaginella moellendorffii* | 4561 | 4 | 15 |
|  | 39729 | 0 | 11 |
|  | 84611 | 6 | 18 |
|  | 417553 | 1 | 29 |
| *Amborella trichopoda* | evm_27.model.AmTr_v1.0_scaffold00010.77 | 0 | 13 |
|  | evm_27.model.AmTr_v1.0_scaffold00012.85 | 6 | 14 |
|  | evm_27.model.AmTr_v1.0_scaffold00021.149 | 4 | 13 |
|  | evm_27.model.AmTr_v1.0_scaffold00023.118 | 0 | 16 |
|  | evm_27.model.AmTr_v1.0_scaffold00044.164 | 0 | 22 |
|  | evm_27.model.AmTr_v1.0_scaffold00051.5 | 0 | 24 |
|  | evm_27.model.AmTr_v1.0_scaffold00119.79 | 0 | 15 |
|  | evm_27.model.AmTr_v1.0_scaffold00405.1 | 1 | 13 |
| *Aquilegia coerulea* | Aqcoe1G464700 | 0 | 14 |
|  | Aqcoe2G057900 | 1 | 26 |
|  | Aqcoe2G168200 | 5 | 15 |
|  | Aqcoe5G119500 | 2 | 11 |
|  | Aqcoe6G134100 | 0 | 11 |
|  | Aqcoe6G173100 | 9 | 13 |
| *Arabidopsis thaliana* | WUS | 1 | 23 |
|  | WOX1 | 2 | 16 |
|  | WOX2 | 0 | 19 |
|  | WOX3 | 2 | 8 |
|  | WOX4 | 1 | 20 |
|  | WOX5 | 4 | 16 |
|  | WOX6 | 0 | 16 |
|  | WOX7 | 0 | 7 |
|  | WOX8 | 1 | 22 |
|  | WOX9 | 3 | 14 |
|  | WOX10 | 1 | 17 |
|  | WOX11 | 7 | 10 |
|  | WOX12 | 0 | 18 |
|  | WOX13 | 5 | 10 |
|  | WOX14 | 2 | 24 |
